# Development of New Drugs to Treat Tuberculosis Based on the Dinitrobenzamide Scaffold

**DOI:** 10.3390/ph17050559

**Published:** 2024-04-27

**Authors:** Tiago Delgado, João P. Pais, David Pires, Filipe G. A. Estrada, Rita C. Guedes, Elsa Anes, Luis Constantino

**Affiliations:** 1 Research Institute for Medicines and Pharmaceutical Sciences (iMed.UL), Av. Prof. Gama Pinto, 1649-003 Lisbon, Portugal; 2 Faculdade de Farmácia, Universidade de Lisboa, Av. Prof. Gama Pinto, 1649-003 Lisbon, Portugal

**Keywords:** tuberculosis, DprE1, DNB, TB, nitrobenzamides

## Abstract

Tuberculosis (TB) continues to be a major global health challenge and a leading cause of death from infectious diseases. Inspired by the results from a previous work by our group on antimycobacterial *N*-alkylnitrobenzamides, which are structurally related to the nitrobenzamide family of decaprenylphosphoryl-β-d-ribose oxidase (DprE1) inhibitors, the present study explored a broad array of substituted benzamides. We particularly focused on previously unexplored 3,5-dinitrobenzamide derivatives. Starting with 3,5-dinitrobenzoic acid, we synthesized a diverse library of amides, incorporating both linear and cyclic amine moieties and also assessed the impact of terminal aromatic groups connected through ether, ester, or amide bonds on the bioactivity of the compounds. The synthesis primarily utilized nucleophilic addition/elimination, S_N_2, and Mitsunobu reactions. The activity was impacted mainly by two structural features, the addition of an aromatic moiety as a terminal group and the type of linker. The most interesting compounds (**c2**, **d1**, and **d2**, MIC = 0.031 μg/mL) exhibited activities against *Mycobacterium Tuberculosis* (Mtb) H37Rv comparable to isoniazid. Complementary computational studies helped elucidate potential interactions with DprE1, enhancing our understanding of the molecular basis of their action. Our findings suggest that the most active compounds provide a promising foundation for the continued development of new antimycobacterial agents.

## 1. Introduction

Tuberculosis has afflicted mankind for thousands of years, and to this day, it remains a leading cause of death from an infectious agent, killing around 1.3 million people every year [1]. TB can be spread with only a small number of bacteria, usually through airborne transmission, for example, when people who are sick with the active disease cough [1,2]. A significant problem in the treatment of TB is its prolonged duration and the number of drugs taken concomitantly, since this leads to a lack of drug compliance by the patients and makes monitorization difficult. For individuals that develop the disease, the current standard treatment of drug-susceptible TB consists of a 6-month drug regimen with the use of four different drugs [1]. When drug resistant variants are factored in, the therapy becomes even more challenging since it requires even longer and more aggressive drug regiments [3,4]. The COVID-19 pandemic made things even worse, and made the “End TB Strategy” goals seem even further away from being achieved, showing that there is an urgent need for more effective drugs against this disease [1].

Mtb has a complex cell wall structure that confers a high degree of impermeability to most drugs due to its hydrophobic character [5]. However, because of its vital role in Mtb survival and the absence of analogous structures in humans, targeting the pathways involved in its synthesis has shown promising results in combating Mtb infections [6,7].

One of the promising targets associated with the cell wall biosynthesis is DprE1, a FAD-dependent oxidoreductase that occupies the periplasm and is essential for the formation of decaprenyl-phospho-d-arabinofuranose (DPA), the only source of arabinose residues available for the synthesis of the arabinogalactan layer of the cell wall, and thus essential for the integrity of the cell wall [6]. 

Several families of compounds can inhibit this enzyme, but they can be mainly divided into two groups: covalent and non-covalent inhibitors. For the covalent inhibitors, an aromatic nitro group is essential for its mechanism of action. The nitro group is reduced by the FAD cofactor of DprE1, forming a nitroso moiety. Then, the sulfur from a nearby cysteine residue, cysteine 387 (Cys387), attacks the nitroso group, forming a covalent bond between the nitro-aromatic group and the enzyme, permanently inhibiting it [8,9].

**Figure 1:**
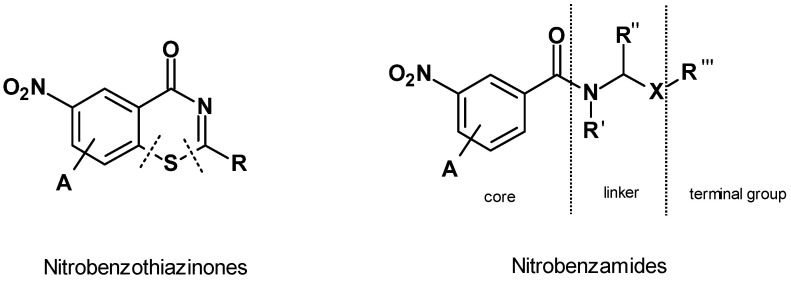
General structure of nitrobenzothiazinones and nitrobenzamides. In this work, we focused modifications on the linker and terminal group of dinitrobenzamides (A = 5-NO_2_); X = N, CH, or O.

The first family of compounds identified as covalent DprE1 inhibitors were the nitrobenzothiazinones (BTZ) [10]. Shortly after, in an independent study, nitrobenzamides were also discovered as covalent inhibitors of DprE1 [11]. This family of compounds can be seen as a simplification of the BTZ structure as taking the benzothiazinone scaffold and opening the heterocycle ring by removing the sulfur atom (Figure 1). 

Nitrobenzamides, as represented in Figure 1, can be broadly divided into three sections: a nitroaromatic benzamide core, containing the nitro group essential for the formation of the covalent bond; a linker, typically comprising a short alkyl structure, a cyclic structure or a combination of both; and a terminal group, commonly an aromatic or aliphatic ring structure. A is generally a second nitro group but can be replaced by another strong electron withdrawing group like a trifluoromethyl [11,12,13,14,15]. DNB1 was one of the first compounds of the nitrobenzamide family to be discovered, and still remains as a reference compound of this family of inhibitors until now [11].

Previous work in our lab uncovered that the nitroaromatic substitution of benzoic acid esters was important for their activity [16]. Following this, we explored a large number of thioesters [17] and amide [18] isosteres of the compounds. A literature search showed that our simple *N*-alkylamides were structurally related to the dinitrobenzamide family of antimycobacterials, where DNB1 is included and two of them were previously reported by Munagala et al. as showing antimycobacterial activity [12].

Following those results, we decided to study a wide range of substituted amides and determine their activity, focusing on unexplored structures related to the nitrobenzamides. We started by synthesizing a library comprised of 3,5-dinitrobenzamides, varying the type of linker and terminal group present and determined their antitubercular activity. Next, we performed computational docking studies and analyzed the position of our compounds within the binding pocket of DprE1 with the objective of trying to further understand how to improve this family of compounds. Three of the nitrobenzamides synthesized were able to have activities comparable to isoniazid and DNB1, and thus are promising leads for the future development of this class of inhibitor. The results are reported here.

## 2. Results

### 2.1. Compound Library

**Scheme 1:**
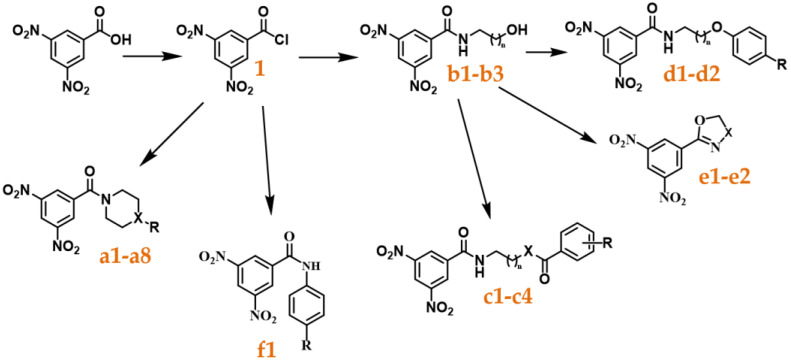
General pathway from the 3,5-dinitrobenzoic acid to the target families of compounds and representation of families **a**–**f**. Each family is represented by a Markush structure. R, X, and n are specified in Table 1. For specific information on the synthetic pathways, please refer to the Supplementary Materials.

With the objective of exploring the effect of new modifications to the linker and the terminal group, a library comprised of six different families of 3,5-dinitrobenzamides (**a**–**f**) was obtained (Scheme 1).

The synthesis of the compounds started from 3,5-dinitrobenzoic acid, which was refluxed in thionyl chloride (SOCl_2_) to originate 3,5-dinitrobenzoyl chloride (**1**). After removing the excess SOCl_2_ by low pressure evaporation, the compound was used without further purification to minimize its degradation. Compound **1** was then coupled with different amines to synthesize compounds **a1**–**a6**, **b1**–**b3**, and **f1** through a nucleophilic addition/elimination reaction, with moderate to good yields (31 to 88%). Compounds **c1** and **c2** were also obtained in a similar manner, but with an inversion of the proportions of the amine and compound **1** being used. (3,5-Dinitrophenyl)-piperazin-1-yl-methanone (**a2**) was used directly for the formation of compound **a8** by the addition of two methyl groups.

**Scheme 2:**
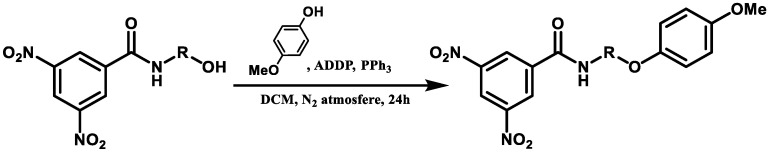
General step for the Mitsunobu reaction represented for the compounds with linear linkers.

Synthesis of compounds **a7**, **d1**, and **d2** was achieved by coupling a 4-methoxyphenol to the terminal hydroxyl group of compounds **a3**, **b2**, and **b3** by the Mitsunobu reaction [19] (reaction outlined in Scheme 2), with low to moderate yields (29 to 43%).

**Scheme 3:**
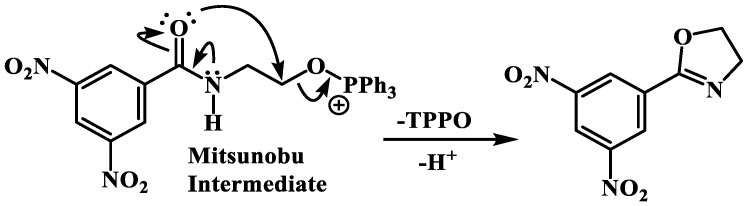
Possible mechanism for the formation of compound **e1** in the Mitsunobu reaction.

In the case of the reaction using **b1** as the starting material, compound **e1** was produced instead of the expected product. During the reaction, there is the formation of an intermediate [19] of the starting material coupled with triphenylphosphine (PPh_3_). In our case, this intermediate likely undergoes an intramolecular reaction (Scheme 3) similar to the one described by Marco Brandstätter et al. [20], since triphenylphosphine oxide (TPPO) is a good leaving group. This reaction had a good yield (70%). Using compound **b2** as a starting material originated compound **e2** as a secondary product and compound **d1** as the main product. The mechanism of **e2** formation is likely similar to that of **e1**, however, in this case, **d1** was also formed and was the major product. Both compounds were able to be isolated by column chromatography.

**Scheme 4:**
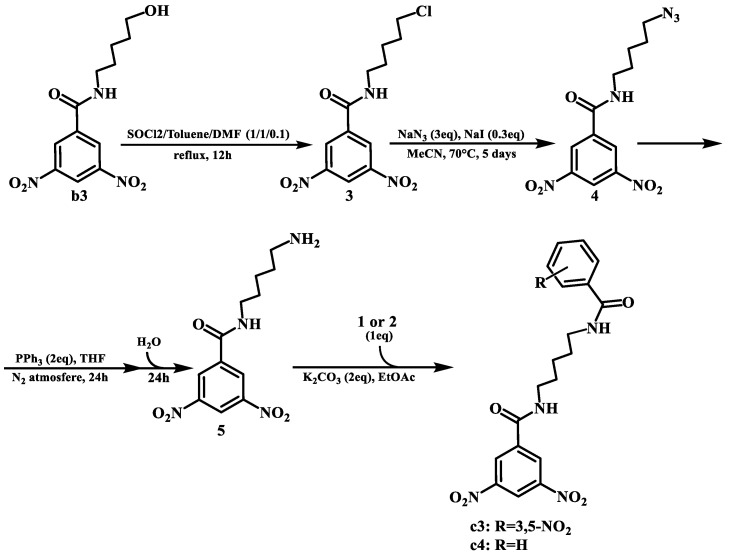
Synthesis of compounds **c3** and **c4**.

Synthesis of *N*,*N*’-(pentane-1,5-diyl)bis(3,5-dinitrobenzamide) (**c3**) and *N*-(5-benzamidopentyl)-3,5-dinitrobenzamide) (**c4**) required four sequential reactions, as outlined in Scheme 4. Compound **b3** was refluxed in a mixture of thionyl chloride and toluene to replace the hydroxyl group by a chloride and originate *N*-(5-chloropentyl)-3,5-dinitrobenzamide (**3**) using DMF as a catalyst. After purification by column chromatography, the compound was obtained with excellent yields (97%). The second step was an S_N_2 reaction to replace the 5-chloro group with an azide to obtain *N*-(5-azidopentyl)-3,5-dinitrobenzamide (**4**) with excellent yields (97%). The third step was the transformation of the azide into an amine using PPh_3_ via the Staudinger reaction [21]. This compound was used directly in the final step, which consisted of a similar reaction to that of the conjugation of the amines with the acyl chlorides, using either compound **1** or benzoyl chloride (**2**). After purification by column chromatography, the compounds were obtained with low/moderate yields (14 to 55%).

### 2.2. Antitubercular Activity

The compounds obtained as well as their corresponding antitubercular activity, determined as minimal inhibitory concentration (MIC) and minimal bactericidal concentration (MBC), are presented in Table 1.

**Table 1:** Library of compounds under study and their corresponding predicted Log*P* values as well as their antitubercular activity against *M. tuberculosis* expressed as MIC and MBC values, the mode value of triplicate experiments tested against the H37Rv strain of Mtb. Refer to Scheme 1 for the Markush structure of each family (**a**–**f**). R, X, and n are as represented below. A list of all compounds and their structure is also presented in the Supplementary Materials. DNB1 shares the Markush structure with the compounds of family **d**; INH: isoniazid.

Compound	R	X	n	Log*P*	MIC (μg/mL)	MIC (μM)	MBC (μM)
**a1**	H	CH	-	1.54	>16	>57.30	>57.30
**a2**	-	NH	-	1.33	ND	ND	ND
**a3**	OH	CH	-	0.77	>16	>54.19	>54.19
**a4**	-	O	-	0.63	>16	>56.90	>56.90
**a5**	Bn	N	-	1.90	2	5.40	10.80
**a6**	Bn	CH	-	2.95	0.5	1.35	5.41
**a7**	OPh-(4-OMe)	CH	-	2.50	2	4.98	9.97
**a8**	Me_2_	N	-	−0.31	>16	>51.73	>51.73
**b1**	OH	-	1	0.24	16	62.70	-
**b2**	OH	-	2	0.50	4	14.86	59.43
**b3**	OH	-	4	1.13	2	6.73	13.46
**c1**	3,5-NO_2_	O	1	1.22	>16	>35.61	>35.61
**c2**	3,5-NO_2_	O	4	2.15	0.063	0.13	0.51
**c3**	3,5-NO_2_	N	4	1.78	0.25	0.51	-
**c4**	H	N	4	2.45	2	5.00	-
**d1**	OMe	-	2	2.38	0.063	0.17	0.33
**d2**	OMe	-	4	2.93	0.063	0.16	0.31
**e1**	-	-CH_2_-	-	0.96	2	8.43	16.87
**e2**	-	-CH_2_CH_2_-	-	1.30	16	63.69	>63.70
**f1**	OMe	-	-	2.00	>16	>50.43	>50.43
DNB1	OMe	-	1	2.04	0.031	0.09	0.09
INH					0.063	0.22	0.43

ND (not determined).

**Figure 2:**
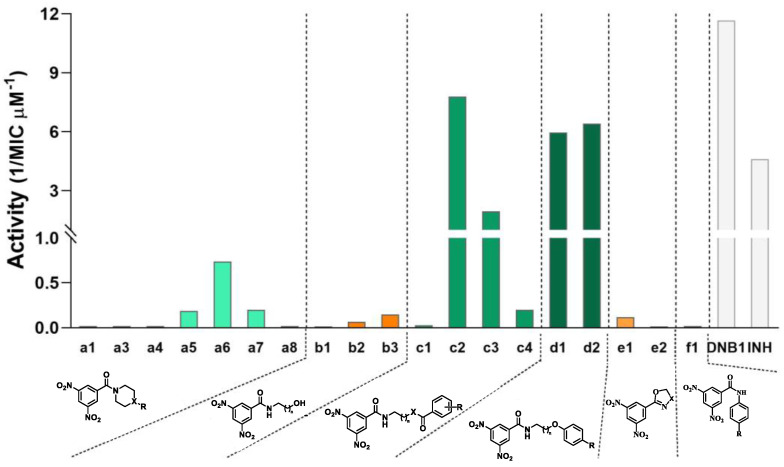
Graphical representation of the antitubercular activity of the compounds under study, grouped by family (**a**–**f**). Activity is represented as 1/MIC, in µM^−1^. Green bars (families **c** and **d** and compounds **a5**, **a6**, and **a7**) denote compounds with an aromatic ring as a terminal group, orange bars (families **b** and **e** and compounds **a1**, **a3**, **a4**, and **a8**) represent compounds lacking a terminal aromatic ring. Compound **f** has a terminal aromatic group but no linker. Grey bars represent control compounds: DNB1 and isoniazid.

In order to better visualize the effect of the structure on the activity, we also represented the antitubercular activities (as 1/MIC) of the different compounds grouped by family (Figure 2). This representation enables a clear observation of the most active compounds, and since 1/MIC is represented, the higher the bar, the more active the compound.

The results show that despite the structural variability, there are two structural features that may impact activity: the addition of an aromatic moiety as a terminal group and the shape and type of the linker. The addition of an aromatic moiety as a terminal group tends to significantly increase the activities of the compounds (Figure 2, orange vs. green bars). One exception is compound **c1**, where that addition led to a compound with lower activity than compound **b1**. The more active compounds also presented a flexible linker in addition to the aromatic group at the end (**c** and **d** families). The more active members of family **a** (**a5**, **a6**, and **a7**) also had a terminal aromatic group but had a less flexible linker and presented lower activities than the compounds from the **c** and **d** families (Figure 2). Elimination of the linker also led to an inactive compound (**f1**).

**Figure 3:**
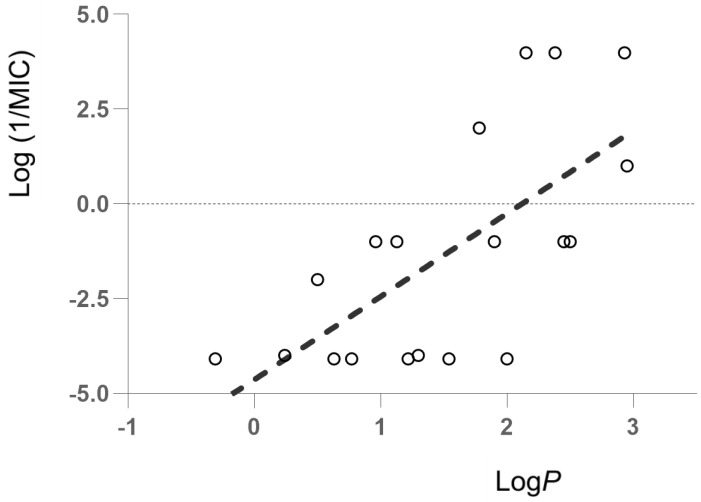
Graphical representation of the correlation between the Log*P* and log(1/MIC) values for the library of compounds. y = 2.1898x − 4.6535; R^2^ = 0.4553.

Within the library, there was some variation in the lipophilicity of the compounds (from −0.31 to 2.98), however, the most active compounds had Log*P* values above 2. Figure 3 illustrates the relationship between Log*P* and antimycobacterial activity for the synthesized compounds.

### 2.3. Computational Studies

A computational docking study was undertaken to investigate the binding interactions of our compounds within the DprE1 binding site using the 4FDN PDB structure [22]. During the structure preparation, a water molecule close to the FAD cofactor was preserved due to its pivotal interactions with both the FAD cofactor and the protein. Docking was performed with the GOLD software [23], from which the highest-scoring poses were selected for analysis.

**Figure 4:**
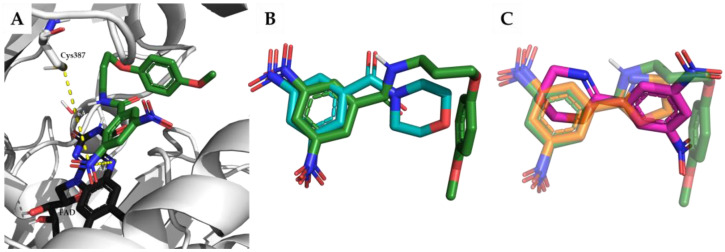
Representation of the best binding poses obtained by the docking computational study between a PDB crystal structure (PDB: 4FDN) and (**A**) the compound **d1** (green) and the distances between the nitro group and both the cysteine residue (Cys387) and the FAD cofactor (black); (**B**) overlay of compound **a4** (cyan) and compound **d1** (green); (**C**) overlay of compounds **e1** (orange) and **e2** (pink) with compound **d1** (green).

The spatial orientation (poses) of the most active compounds demonstrated proximity to both Cys387 and the FAD cofactor. The inhibitors’ mechanism of action, requiring the nitro group’s activation by FADH_2_, followed by covalent bonding with Cys387’s sulfur, necessitates that the compound enters the active site in a way that positions its nitro group near these critical regions. Thus, the placement of the nitro groups of the most active compounds near these zones was expected (exemplified by compound **d1** in Figure 4A). However, some less active compounds were also found in similar positions, as can be seen in Figure 4B. 

Curiously, half of the compounds exhibiting low activities (>2 μg/mL) were oriented inversely within the pocket compared to compound **d1**. An example of this is compound **e2** (illustrated in Figure 4C), where its 5,6-dihydro-4*H*-1,3-oxazine ring is positioned similarly to the nitroaromatic core of compound **d1**, albeit with the opposite overall orientation. This stands in contrast to compound **e1**, which despite its structural resemblance to **e2**, assumes a position within the pocket that is more aligned with the orientation of compound **d1**. This observation highlights the nuanced role of molecular orientation in the binding efficacy and activity levels of these compounds.

To conduct a more comprehensive analysis of these findings, we calculated the distances between the nitro groups and two key sites: Cys387 (ND_Cys387_, which is the distance from the nitrogen atom of the closest nitro group to the sulfur of Cys387) and the FAD cofactor (ND_FAD_, the distance from the nitrogen atom of the nearest nitro group to the nitrogen atom in the central portion of the FAD’s isoalloxazine ring). These critical distances, along with the docking scores, are detailed in Table 2. 

**Figure 5:**
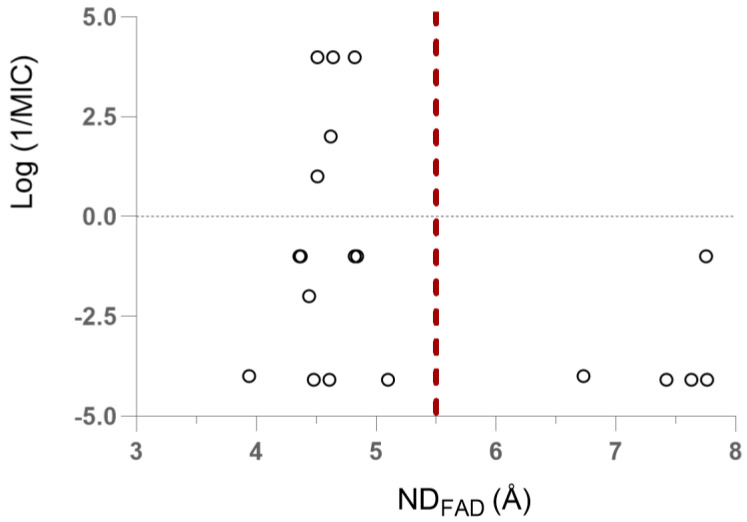
Relation between the activity of the compounds and the distance of the nitrogen atom of the nitro group to the nitrogen in the center portion of the isoalloxazine ring of the FAD cofactor. Distances above 5.5 Å (at right side of the dotted line) appear to lead to compounds with low activity.

Figure 5 demonstrates the correlation between the distance to FAD and the antitubercular activity. This visualization indicates that there is no clear separation between the active and non-active compounds on these metrics. However, an observation from the data suggests that ND_FAD_ values greater than 5.5 Å are commonly found in compounds characterized by lower activity levels. This insight might imply a threshold effect where distances beyond a certain point adversely affect the compound’s antitubercular potency.

**Figure 6:**
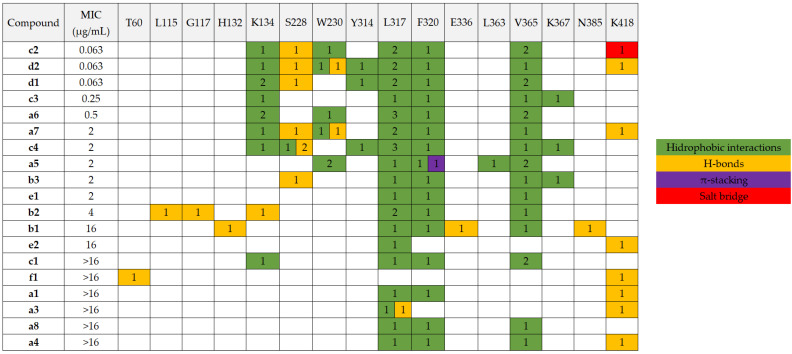
Map of interactions between the best poses from the non-covalent docking of the compounds and DprE1.

Additionally, the type of interactions formed between each compound and the amino acid residues within the DprE1 pocket were identified using Protein–Ligand Interaction Profiler (PLIP) [24] (Figure 6).

Residues K134, S228, and W230 were identified as critical interaction points for the most active compounds, whereas L317, F320, and V365 played a significant role for all investigated compounds.

The absence of a correlation between docking score and the activity in the non-covalent docking, combined with the inhibition mechanism involving a covalent bond formation between the inhibitor and protein, prompted the application of covalent docking for our compounds. In this covalent docking scenario, a correlation between the docking scores and compound activity was noted (y = 0.2614x − 8.6348; R^2^ = 0.4452), with the most active compounds displaying higher docking scores. Furthermore, docking scores below 30 were found to correspond to compounds exhibiting lower activity, indicating a potential threshold for predicting compound efficacy. These docking scores are presented in Table 2.

Considering the correlations observed between the bioactivity, quantified as log(1/MIC), and both the lipophilicity (Log*P*) and covalent docking scores, we employed a multivariate linear regression with least squares optimization to model these relationships and derived the equation: Bioactivity = 0.5958 × Log*P* + 0.07253 × Docking Score − 3.265. The resultant model exhibited a strong predictive capability, as evidenced by a correlation coefficient of 0.9061 (R = 0.9061, R^2^ = 0.8211) between the predictors and the biological activity.

## 3. Discussion

In the first family of compounds (**a**), the linker consists of one heterocyclic structure containing a nitrogen atom and X can be CH, N, or O. When X is CH or N, a terminal R can be present, with either an H, OH, Me_2_, benzyl, or OPh-(4-OMe) as substituents. By analyzing the activities, we can see that there are two main sets of compounds in the family: one with moderate activities against Mtb (0.5 to 2 μg/mL) that have a terminal aromatic ring as a terminal group, and a second set of compounds that presents smaller groups in the terminal position and that fail to have significant activity against Mtb (>16 μg/mL). This reveals that the aromaticity and/or lipophilicity that the addition of the aromatic moieties impart to the compounds is very beneficial to their activity.

In family **b**, whose compounds have linear alkyl chains of variable size as linkers (two, three, or five carbons) but no terminal group, there is an increase in activity as the linkers become bigger (and the lipophilicity increases).

Family **c** consists of compounds where linear linkers with two or five carbons are connected to the terminal aromatic rings in two ways: ester or amide bonds. Between compounds **c1** and **c2** (esters) the only variation is the linker size (two and five carbons, respectively), with both compounds having a (3,5-dinitrobenzoyl)oxy as a terminal group. This, however, had a big impact on the activity of the compounds, with the first compound not showing any meaningful activity and the second one showing activity comparable to INH and DNB1. We have no clear rationale to explain the difference in activity between **c1** and **c2**. This difference could be attributed in part to the lipophilicity of the compounds. Compound **c1**, with its two charged nitro groups on each end of the molecule and a short linker, may face some difficulty in passing through the lipophilic cell envelope of the mycobacteria. Compound **a8** may likely encounter a similar problem due to the presence of a positively charged quaternary amine in the terminal group’s zone. Compounds **c2** and **c3** allow for a comparison of the influence of the connection between the linker and the terminal group on the activity, with the ester bond being preferred over the amide bond. Finally, between the two diamides (**c3** and **c4**), the best was the one with two nitroaromatic cores in the molecule (**c3**), despite **c4** being the most lipophilic of this family of compounds. However, this is not surprising considering that **c3** is symmetrical, and both extremities could covalently bond to DprE1, while only one could do it in the **c4** molecule.

The addition of the terminal aromatic ring to compounds **b2** and **b3** by an ether bond to obtain **d1** and **d2**, respectively, led to a significant increase in the activity of the compounds. The difference in size of the linker did not influence the activity, with both compounds showing equal antitubercular activities that were comparable to INH and DNB1. 

Except for compound **c1**, both the **c** and **d** families showed a similar trend to family **a**, where the addition of terminal aromatic moieties to the compounds was beneficial to their activity, reinforcing the importance of this section of the molecule. This is also in accordance with the literature, which describes a preference for the substitution of a phenyl group over an alkyl group in the terminal group zone [12].

The simple addition of an aromatic ring is not sufficient, however, to obtain good MIC values, with the linker also playing an important part in the activity of the compounds, since connecting an aromatic ring directly to the amide of the nitrobenzamide core (compound **f1**) resulted in an inactive compound. The cyclic linkers (family **a**) also seemed to have lower activities than the linear linkers (families **c** and **d**), likely because of the added steric hindrance and/or the lower flexibility.

These observations are partially consistent with the literature, with the absence of the linker being described as detrimental to the activity [12], and the use of cyclic linkers or substituents higher than methyl in the first carbon of the linker near the amide, also being detrimental to the activity [11,12]. However, although some of the literature has reported that the increase in the linker size leads to a decrease in potency [12], our compounds demonstrate that it can at least maintain the potency in the series created, since DNB1, **d1**, and **d2** presented comparable activities.

Although there are other factors that also play a role in the activity, lipophilicity is a property with a major impact on antitubercular activity. By calculating the lipophilicity of the compounds and plotting log P against their activity, we can see that the more active compounds are, in general, more lipophilic than the less active ones (Figure 3). This is not surprising, given the characteristics of the cell wall of Mtb, which is effective at preventing the entry of the more polar compounds [25,26]. Thus, this is an important aspect to bear in mind when further developing this class of inhibitors.

In the docking analysis with compounds **c2**, **d1**, and **d2**, the optimal poses of these lead candidates are situated near FAD and cysteine, two critical sites for the inhibition mechanism of DprE1 by nitrobenzamides. Specifically, their proximity to FAD, essential for the initial step in this mechanism, suggests that the benzamides studied here likely employ a similar inhibition mechanism as other nitrobenzamides documented in the scientific literature. This analysis may also shed light on the differing activities observed between compounds **e1** and **e2**. Unlike compounds **b1** and **b2**, the ‘shorter’ compound (implied to be **e1**) showed higher activity. This could be due to the positioning of **e1** in the protein’s pocket, which aligns with that of the active compounds. In contrast, **e2** was oriented in reverse, with its 5,6-dihydro-4H-1,3-oxazine ring in a position akin to the nitroaromatic core of the active compounds. However, this observation should be interpreted with caution since some inactive compounds also share similar positioning to the active compounds. Thus, positioning alone may not be a reliable criterion for future development within this class of inhibitors. Nevertheless, plotting the distance from the nitro group to FAD against activity indicates that distances greater than 5.5 Å tend to be associated with compounds exhibiting lower activity. Additionally, the results from the covalent docking, particularly scores below 30, suggest lower activity. Moreover, a multivariate linear regression correlating the activity with both the Log*P* values and the covalent docking score showed a good correlation coefficient (r = 0.9061). This regression model could be useful to predict the activity of future compounds based on their Log*P* values and covalent docking scores before experimental testing. These findings could be instrumental in guiding future synthetic modifications.

Additionally, looking at the list of interactions that the best poses of the compounds make with the receptor, some key interactions that are important for the activity of this class of compounds were revealed. In particular, the interaction with K134 appeared in all of the active compounds and some of the moderately active compounds, while it failed to appear in the compounds with low activity, and thus may be an important interaction with the receptor. Interactions with S228 and W230 also appeared more frequently in the more active compounds. Indeed, K134 and W230 have been reported as important residues that establish interactions with some of the DprE1 inhibitors, and S228, together with K134 and some other residues, form a hydrophilic cavity in the enzyme that is important to the affinity of the inhibitors [27]. Thus, for the future development of these compounds, those that are able to make these interactions may have better activities. However, other interactions have also been reported and may be important for the activity [27], and thus may also be worth considering, especially if the characteristics (e.g., size) of the compounds start to considerably deviate from the ones studied here.

## 4. Materials and Methods

### 4.1. Materials

Balanced salt solution, phosphate-buffered saline (PBS), Dulbecco’s modified Eagle’s medium (DMEM), and L-glutamine were purchased from Invitrogen. Sodium dodecyl sulfate (SDS), Triton X-100, 3,5-dinitrobenzoic acid, benzoic acid, piperidine, 4-benzylpiperidine, 4-hydroxypiperidine, piperazine, 4-benzylpiperazine, morpholine, 2-aminoethan-1-ol, 3-aminopropan-1-ol, 5-aminopentan-1-ol, 4-methoxyphenol, *p*-anisidine, triphenylphosphine, 1,1’-(azodicarbonyl)dipiperidine (ADDP), iodomethane, potassium carbonate, sodium iodide, sodium azide, thionyl chloride, and trypan blue were purchased from Merck, KGaA (Darmstadt, Germany). All solvents were used without purification as acquired from commercial sources. Middlebrook 7H9, 7H10 agar, and OADC (oleic acid, albumin, dextrose, catalase) supplement were purchased from Becton Dickinson (Franklin Lakes, NJ, USA). Glycerol and tyloxapol were purchased from Merck, KGaA (Darmstadt, Germany). Nunc Microwell tissue culture plates were purchased from Thermo Fisher Scientific (Waltham, MA, USA). All amides presented were synthesized according to the procedures described in this paper. Compounds were prepared in stock solutions of 40 mg/mL in dimethyl sulfoxide (DMSO—AppliChem Panreac, Darmstadt, Germany). Isoniazid (Merck, KGaA, Darmstadt, Germany) is a first line antibiotic against tuberculosis and was used as a positive control for *M. tuberculosis* killing. 

### 4.2. Bacterial Strains and Cell Lines

*M. tuberculosis* H37Rv ATCC 27294 (American Type Culture Collection, Manassas, VA, USA) was used for MIC and MBC evaluation. These bacteria were cultivated in Middlebrook’s 7H9 medium supplemented with 10% OADC (oleic acid, albumin, dextrose, catalase) enrichment, 0.02% glycerol, and 0.05% tyloxapol. Bacteria were grown on non-treated polystyrene square culture flasks (VWR International) incubated at 37 °C until the exponential growth phase was achieved. All assays using *M. tuberculosis* were performed in the Biosafety Level 3 laboratory at the Faculty of Pharmacy of the University of Lisbon (Lisbon, Portugal), following the respective national and European biosecurity standards, based on applicable EU Directives.

### 4.3. Synthesis

#### 4.3.1. Acyl Chloride Synthesis

A solution of 3,5-dinitrobenzoic acid or benzoic acid in thionyl chloride (2 mL per mmol of acid) was refluxed for 12 h, leading to the formation of the respective acyl chloride. The excess thionyl chloride was removed by low pressure evaporation. The product was used without further purification. 

#### 4.3.2. General Protocol to Amide Synthesis

A solution of the appropriate acyl chloride (1 eq.) in ethyl acetate was added dropwise to a solution of corresponding amine (2 eq.) and K_2_CO_3_ (2 eq.) in ethyl acetate. When the reaction was complete (as assessed by TLC using hexane:ethyl acetate, 3:7 to 0:1 as the eluent) the reaction mixture was filtered, and the filtrate washed successively with 10 mL of distilled water and 15 mL of brine. The ethyl acetate solution was subsequently dried and the solvent evaporated. The residue was purified by column chromatography (silica gel 60) using hexane:ethyl acetate, 1:1 to 2:8 as the eluent.

#### 4.3.3. General Protocol to Mitsunobu Reaction [19]

A solution of the appropriate product (1 eq.), the respective phenol (1 eq.), and ADDP (2.5 eq.) in dichloromethane was placed under an atmosphere of nitrogen and for 10–15 min purged with nitrogen. Then, to this mixture, a solution of PPh_3_ (2.5 eq.) in dichloromethane was added dropwise, over a 2-minute period, and the reaction was stirred at room temperature for around 24 h, all while keeping the reaction under a nitrogen atmosphere. After this, the DCM was evaporated, the mixture was dissolved in EtOAc, and washed successively with 10 mL of distilled water and 10 mL of brine. The ethyl acetate solution was subsequently dried and the solvent evaporated. The residue was purified by column chromatography (silica gel 60) using hexane:ethyl acetate, 8:2 to 1:1 as the eluent.

#### 4.3.4. General Protocol to the Diamide Formation

For the synthesis of these compounds, four different steps were required. 

The first one was replacing the hydroxyl group with a chloro. For this, a solution of **b3** in a mixture of thionyl chloride, toluene, and DMF (1/1/0.1, in a total volume of 3 mL per mmol of reactant) was refluxed for 12 h, leading to the formation of the desired product. The excess solvent mixture and thionyl chloride were removed by low pressure evaporation. Then, the mixture was dissolved in EtOAc and washed successively with 10 mL of distilled water and 10 mL of brine. The ethyl acetate solution was subsequently dried, and the solvent evaporated. The residue was purified by column chromatography (silica gel 60) using hexane:ethyl acetate, 8:2 to 1:1 as the eluent.

The second step was an S_N_2 reaction to replace the chlorine with an azide. For this, a solution of the previous product, NaN_3_ (3 eq.), and NaI (0.3 eq.) in MeCN were heated at 70 °C and stirred for 5 days. The MeCN was then removed by low pressure evaporation and the mixture was dissolved in EtOAc and washed successively with 10 mL of distilled water and 10 mL of brine. The ethyl acetate solution was subsequently dried, and the solvent evaporated. The residue was purified by column chromatography (silica gel 60) using hexane:ethyl acetate, 8:2 to 1:1 as the eluent.

The third step was the Staudinger reaction [21] to transform the azide into an amine. For this, the previous product and PPh_3_ (2 eq.) were dissolved in THF under an N_2_ atmosphere and stirred for 24 h. Then, a small quantity of distilled water was added to the mixture, and the reaction was stirred for another 24 h. The solvent was then evaporated, and the product used without further purification.

The fourth and final step consisted of repeating the general protocol for amide synthesis with the amine product, utilizing both 3,5-dinitrobenzoyl chloride and benzoyl chloride to yield **c3** and **c4**, respectively.

All final compounds were characterized by ^13^C NMR, ^1^H NMR, and MS. The purity of the compounds was further tested by TLC. Synthesis specifications, yields, and structural data for the compounds are available in the Supplementary Materials.

### 4.4. Compound Characterization

^1^H NMR and ^13^C NMR spectra were recorded on a Bruker Avance 400 spectrometer in the indicated solvent; chemical shifts are reported in parts per million (ppm), relative to tetramethylsilane (TMS). The spectra were referenced to the solvent peak, and coupling constants (J) are quoted in hertz (Hz). The mass spectra were recorded on an Acquity^TM^ triple quadrupole spectrometer (ESI); compounds were analyzed in a solution of MeCN, and *m*/*z* values are reported in Daltons.

### 4.5. Determination of the Minimum Inhibitory Concentration (MIC) and Minimum Bactericidal Concentrations (MBC)

MICs were determined by an adaptation of the broth microdilution method in 96-well plates [28,29]. Briefly, *M. tuberculosis* bacterial cultures in the exponential growth phase were collected by centrifugation, washed with PBS, and re-suspended in fresh culture medium (Middlebrook’s 7H9). Clumps of bacteria were removed by ultrasonic treatment of the bacteria suspension in an ultrasonic water bath for 5 min, followed by low-speed centrifugation (500× *g*) for 2 min. Single cell suspension was verified by microscopy. The microplates containing a bacterial suspension corresponding to approximately 10^5^ colony-forming units per mL, evaluated through optical density measurements, were incubated with the selected concentrations of the compounds.

Every other day, the optical density of the wells was measured with a Tecan M200 spectrophotometer, following 30 s of orbital agitation. These values were used to produce the growth curves. At the 10th day of incubation, the MIC was determined, corresponding to the concentration with no visible turbidity. Optical density measurements were taken until the 15th day of incubation. The MBC values were determined using a methodology established by us [28]. Briefly, following MIC determination, 5 µL of each suspension was recovered from the MIC test microplates and spotted onto 7H10 agar plates supplemented with OADC. The MBC was determined following 3 weeks of incubation, corresponding to the concentration of compound that produced no spots (bacterial growth) on the solid medium. Bacteria treated with DMSO solvent at the same proportions as present during the compound tests were used as a control. Isoniazid was used as a positive control for bacteria killing and assay validation following EUCAST guidelines (MIC = [0.03, 0.12] μg/mL). All MIC and MBC results presented comprise the mode value of a minimum of triplicate experiments.

### 4.6. LogP Determination

The predicted Log*P* values were calculated using SwissADME (http://www.swissadme.ch/, accessed on 15 January 2024 [30]) and correspond to the average of the values from iLOGP, XLOGP3, WLOGP, and MLOGP.

### 4.7. Molecular Docking

The receptor structure was retrieved from RCSB Protein Data Bank entry 4FDN (PDB: 4FDN), which corresponds to the crystal structure of *M. tuberculosis* DprE1 in complex with the covalent inhibitor CT325. Initially, all water molecules and ligands, except for water molecule 1035, were removed. The protein’s structure was then prepared using the Molecular Operating Environment (MOE2020.01) software. Within this environment, missing loops were built using the “Build Loop” tool, structural errors corrected, and charges and protonation states assigned via the “Protonate 3D” tool, applying standard conditions (pH = 7 and T = 300 K). The refined structure was saved in mol2 format.

The 3D models of the compounds under study were prepared with MOE. The SMILES strings of the compounds were obtained and then input to MOE’s database and processed sequentially through the “Wash”, “Partial Charges”, and “Energy Minimization” steps using default parameters before being saved as mol2 files.

Molecular docking simulations were conducted using GOLD software (Version 2022.1.0) [23] with the default values and using the Astex Statistical Potential (ASP) scoring function. A search space with a radius of 20 Å centered on the ligand from PDB entry 4FDN was defined, and each ligand underwent 100 docking runs to ensure robust sampling and reproducibility of the results. Poses were ranked according to the scoring functions by selecting the lowest-energy conformation for each compound. The coordinates of the top pose for each compound were used to calculate the distance from the nitrogen atom of their nitro groups to two critical receptor atoms: the sulfur atom in Cys387 and the nitrogen atom in the central part of the isoalloxazine ring of the FAD cofactor within the binding pocket. Covalent docking was also conducted using the ASP scoring function, for which compounds were modified by removing an oxygen atom from one of the nitro groups, and the nitrogen of this modified nitro group and the sulfur of Cys387 were designated for covalent bond formation. 

The Protein–Ligand Interaction Profiler (PLIP) (https://plip-tool.biotec.tu-dresden.de/plip-web/plip/index, accessed on 20 January 2024) was used to identify the interactions between the DprE1 structure and the best poses from the non-covalent docking of each compound, providing comprehensive insights into the binding mechanisms [24].

## 5. Conclusions

The synthesis of a library of dinitrobenzamides allowed us to enhance the understanding of the role of the linker and the terminal group in the antitubercular activity of the compounds. The results here discussed show that high antitubercular activities are obtained when an aromatic terminal group is present, with linear linkers being more favorable than cyclic linkers. They also show the importance of the linker in the activity since its omission led to an inactive compound. Another characteristic that has a significant effect in the activity is the lipophilicity of the compounds. Computational studies indicate that the most active compounds are placed in the expected area according to the mechanism of inhibition. Nevertheless, the methodology lacks the discriminatory capability to fully distinguish between active and non-active compounds. However, looking at ND_FAD_, the covalent docking score and the interactions established could allow us to filter out some of the inactive compounds. The regression model could also be useful to predict the activity of future compounds based on their covalent docking score and Log*P*. Among the synthesized compounds, those belonging to the **d** family and compound **c1** have demonstrated significant potential, exhibiting activities capable of rivalling that of isoniazid and also retaining the activity level observed in other inhibitors within this class, like DNB1. Thus, these compounds stand as promising candidates to be lead molecules in the future development of nitrobenzamides. Additionally, insights into the required lipophilicity and docking “benchmarks” for prospective compounds have been revealed, serving as valuable guidelines to effectively prioritize synthesis efforts, and the synthetic pathways utilized can be easily adapted in future synthetic endeavors.

## Figures and Tables

**Table 2:** Values of the distances obtained from the docking analysis of the best binding poses of the compounds in the study and their correspondent scores and MIC (µg/mL) values.

Compound	ND_Cys387_ (Å)	ND_FAD_ (Å)	Score (Non-Covalent Docking)	Score (Covalent Docking)	MIC (μg/mL)
**a1**	4.42	7.63	30.97	20.08	>16
**a3**	4.49	7.76	32.39	26.93	>16
**a4**	7.16	5.10	31.17	28.62	>16
**a5**	6.16	7.75	36.37	23.61	2
**a6**	6.32	4.51	33.10	25.76	0.5
**a7**	6.25	4.84	33.06	28.86	2
**a8**	5.70	4.48	35.60	24.21	>16
**b1**	5.91	3.94	35.18	30.54	16
**b2**	6.30	4.44	35.43	28.03	4
**b3**	6.41	4.36	37.52	31.88	2
**c1**	5.57	4.61	23.69	17.11	>16
**c2**	7.62	4.82	26.78	38.33	0.063
**c3**	5.96	4.62	29.09	37.62	0.25
**c4**	5.75	4.82	43.45	37.28	2
**d1**	6.35	4.64	42.43	33.77	0.063
**d2**	6.33	4.51	42.44	37.25	0.063
**e1**	6.22	4.37	33.42	27.67	2
**e2**	4.36	6.73	32.40	28.33	16
**f1**	5.80	7.42	36.10	7.78	>16

## Data Availability

Data available within the article.

## Supplementary Materials

The following supporting information can be downloaded at: https://www.mdpi.com/article/10.3390/ph17050559/s1, Synthesis details and structural characterization.
