# Supplementary material for: Development of New Drugs to Treat Tuberculosis Based on the Dinitrobenzamide Scaffold
Source: Pharmaceuticals (Basel). 2024 Apr 27;17(5):559. doi: 10.3390/ph17050559 (PMC11124350; doi:10.3390/ph17050559)
Supplement: Supplementary file 1 [file pharmaceuticals-17-00559-s001.zip › pharmaceuticals-2937534-supplementary.pdf]

Supplementary materials

Development of New Drugs to Treat Tuberculosis Based on the Dinitrobenzamide Scaffold

Delgado *et al.*

S1. Synthesis details and structural characterization:

S1.1. Acyl chloride synthesis:

General procedure: A solution of the carboxylic acid in thionyl chloride (2 mL per mmol of acid) was refluxed for 12 h, leading to the formation of the respective acyl chloride. The excess thionyl chloride was removed by low pressure evaporation. The product was used without further purification.

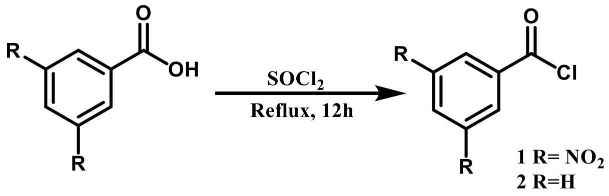

Figure S1.1: General synthetic scheme for the acyl chloride synthesis

Synthesis of 3,5-dinitrobenzoyl chloride (**1**). Following the described general procedure for the acyl chloride synthesis, 500 mg (2.36mmol) of 3,5-dinitrobenzoic acid in 4.5 mL of thionyl chloride were refluxed for 12 h. This yielded a white-yellowish solid that was used without further treatment.

Synthesis of benzoyl chloride (**2**). Following the described general procedure for the acyl chloride synthesis, 61 mg of benzoic acid (0.5mmol) in 2.5 mL of thionyl chloride were refluxed for 12 h. This yielded a white-yellowish solid that was used without further treatment.

S1.2. Amide synthesis:

General procedure: A solution of the appropriate acyl chloride (1 eq.) in ethyl acetate was added dropwise to a solution of corresponding amine (2 eq.) and K_2CO_3 (2 eq.) in ethyl acetate. When the reaction was complete (as assessed by TLC using hexane:ethyl acetate, 3:7 to 0:1 as eluent) the reaction mixture was filtered, and the filtrate washed successively with 10 mL of distilled water and with 15 mL of brine. The ethyl acetate solution was subsequently dried, and the solvent evaporated. The residue was purified by column chromatography (silica gel 60) using hexane: ethyl acetate, 1:1 to 2:8 as eluent.

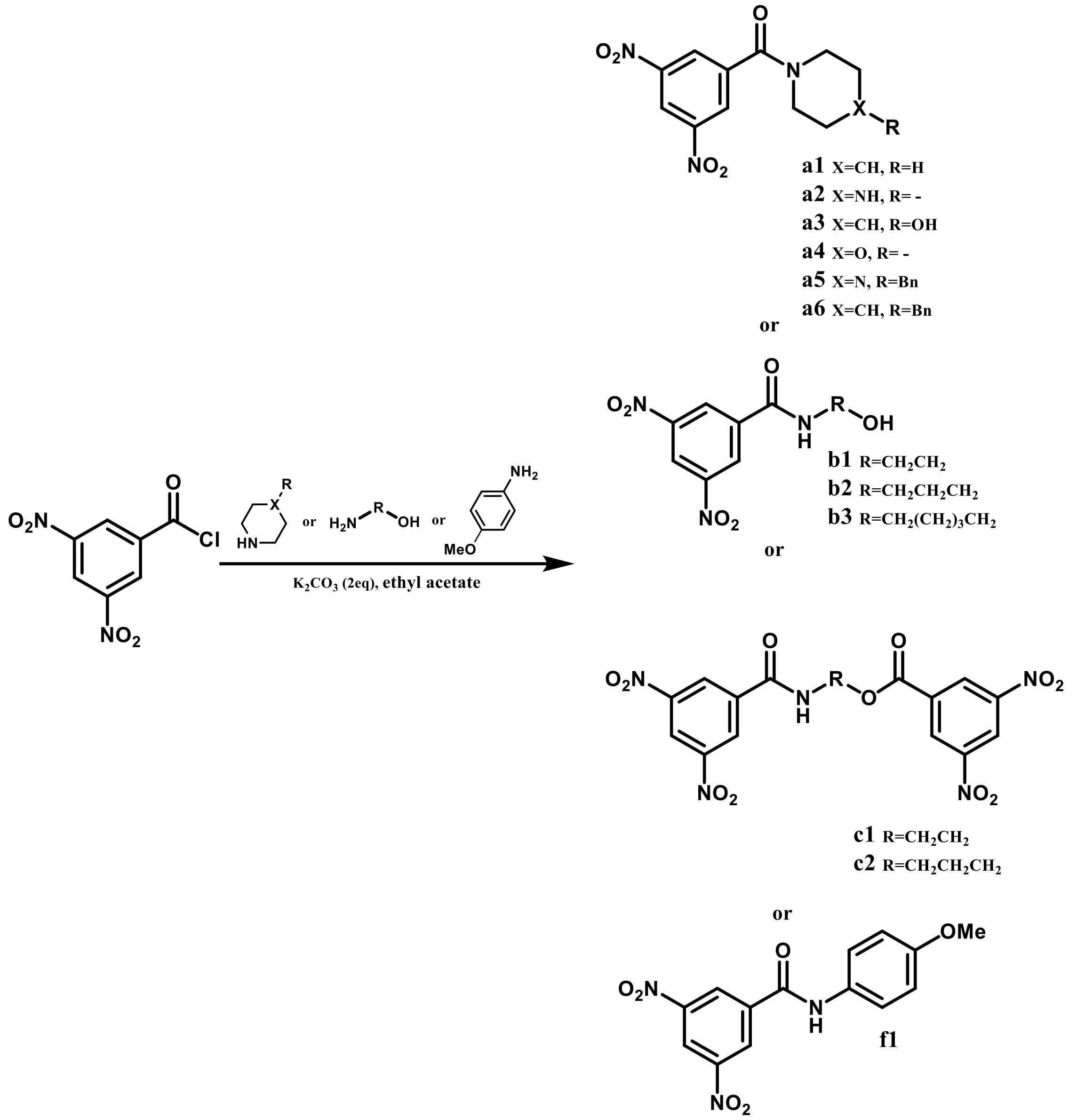

Figure S1.2: General synthetic scheme for the amide synthesis

Synthesis of (3,5-dinitrophenyl)-piperidin-1-yl-methanone (**a1**). Following the described general procedure for the amide synthesis, 254mg (1.1mmol) of compound **1** was dissolved in ethyl acetate (5 mL) and was added to a solution in ethyl acetate (5 mL) of 187 mg (2.2mmol) of piperidine and 304mg (2.2mmol) of K_2CO_3. The compound was a white/yellowish solid. Yield=57%. ^{1}H NMR (400 MHz, $CDCl_3$) δ 9.08 (t, J = 2.1 Hz, 1H), 8.58 (d, J = 2.2 Hz, 2H), 3.76 (s, 2H), 3.35 (s, 2H), 1.74 (s, 4H), 1.59 (s, 2H). ^{13}C NMR (101 MHz, $CDCl_3$) δ 165.24, 148.59, 139.91, 127.43, 119.57, 49.11, 43.80, 26.65, 25.49, 24.36. m/z calculated for 280.09280, found: 280.11 (M+H+).

Synthesis of (3,5-dinitrophenyl)-piperazin-1-yl-methanone (**a2**). Following the described general procedure for the amide synthesis, 461 mg (2 mmol) of compound **1** was dissolved in ethyl acetate (5 mL) and was added to a solution in ethyl acetate (5 mL) of 345mg (4 mmol) of piperazine and 553 mg (4 mmol) of K_2CO_3. The product was used directly in the synthesis of **a8**.

Synthesis of (3,5-dinitrophenyl)-4-hydroxypiperidin-1-yl-methanone (**a3**). Following the described general procedure for the amide synthesis, 254 mg (1.1mmol) of compound **1** was dissolved in ethyl acetate (5 mL) and was added to a solution in ethyl acetate (5 mL) of 223mg (2.2mmol) of 4-hydroxypiperidine and 254mg (2.2mmol) of K_2CO_3. The compound was a white/yellowish solid. Yield=52%. ^{1}H NMR (400 MHz, $CDCl_3$): δ 9.09 (t, J = 2.2 Hz, 1H), 8.59 (d, J = 2.1 Hz, 2H), 4.08 (m, 2H), 3.62 (s, 2H), 3.29 (d, J = 11.6 Hz, 1H), 2.01 (s, 1H), 1.88 (s, 1H), 1.74 (s, 2H), 1.59 (s, 1H). ^{13}C NMR (101 MHz, $CDCl_3$) δ 165.38, 148.63, 139.50, 127.44, 119.78, 66.24, 44.98, 39.74, 34.06, 33.54. m/z calculated for 296.08771, found: 296.08 (M+H+).

Synthesis of (3,5-dinitrophenyl)(morpholino)methanone (**a4**). Following the described general procedure for the amide synthesis, 161 mg (0.7mmol) of compound **1** was dissolved in ethyl acetate (5 mL) and was added to a solution in ethyl acetate (5 mL) of 122 mg (1.4 mmol) of morpholine and 194 mg (1.4 mmol) of K_2CO_3. The compound was a white solid. Yield=88%. ^{1}H NMR (400 MHz, $CDCl_3$): δ 9.09 (t, J = 2.3 Hz, 1H), 8.60 (d, J = 2.2 Hz, 2H), 3.83 (s, 4H), 3.69 (s, 2H), 3.46 (s, 2H). ^{13}C NMR (101 MHz, $CDCl_3$) δ 165.38, 148.67, 138.76, 127.67, 119.99, 66.71, 48.35, 43.03. m/z calculated for 282.07206, found: 282.04 (M+H+).

Synthesis of (3,5-dinitrophenyl)-4-benzylpiperazin-1-yl-methanone (**a5**). Following the described general procedure for the amide synthesis, 161mg (0.7mmol) of compound **1** was dissolved in ethyl acetate (4 mL) and was added to a solution in ethyl acetate (4 mL) of 159mg (0.9 mmol) of 4-benzylpiperazine and 415mg (3mmol) of K_2CO_3. The compound was a white solid. Yield=88%. ^{1}H NMR (400 MHz, DMSO-d6) δ 8.85 (t, J = 2.2 Hz, 1H), 8.61 (d, J = 2.1 Hz, 2H), 7.38 – 7.19 (m, 6H), 3.66 (t, J = 5.1 Hz, 2H), 3.52 (s, 58 2H), 3.33 (br s, 2H), 2.47 (d, J = 5.1 Hz, 2H), 2.36 (t, J = 5.2 Hz, 2H). ^{13}C NMR (101 MHz, DMSO-d6) δ 164.74, 148.17, 138.87, 137.78, 128.93, 128.26, 127.55, 127.10, 119.15, 61.74, 52.44, 51.77, 47.10, 41.87. m/z calculated for 371.13500, found: 370.85 (M+H+).

Synthesis of (3,5-dinitrophenyl)-4-benzylpiperidin-1-yl-methanone (**a6**). Following the described general procedure for the amide synthesis, 254mg (1.1mmol) of compound **1** was dissolved in ethyl acetate (5 mL) and was added to a solution in ethyl acetate (5 mL) of 386 mg (2.2mmol) of 4-benzylpiperidine and 304mg (2.2mmol) of K_2CO_3. The compound was a white solid. Yield=53%. ^{1}H NMR (400 MHz, $CDCl_3$): δ 9.08 (t, J = 2.1 Hz, 1H), 8.58 (d, J = 2.1 Hz, 2H), 7.30 (t, J = 7.4 Hz, 2H), 7.22 (d, J = 7.2 Hz, 1H), 7.15 (d, 2H), 4.70 (d, J = 13.3 Hz, 1H), 3.57 (d, J = 13.5 Hz, 1H), 3.11 (t, J = 13.2 Hz, 1H), 2.81 (t, J = 12.9 Hz, 1H), 2.61 (t, J = 6.5 Hz, 2H), 1.91-1.79 (m, J = 12.0, 8.0, 4.0 Hz, 2H), 1.69 (d, J = 13.2 Hz, 1H), 1.36 (d, J = 13.1 Hz, 1H), 1.21 (d, J = 13.4 Hz, 1H). ^{13}C NMR (101 MHz, $CDCl_3$) δ 165.25, 148.60, 139.81, 139.56, 129.18, 128.56, 127.45, 126.40, 119.64, 48.40, 43.13, 42.86, 38.22, 32.69, 31.63. m/z calculated for 370.13975, found: 370.31 (M+H+).

Synthesis of 4-(3,5-dinitrobenzoyl)-1,1-dimethylpiperazin-1-ium (**a8**). Following the synthesis of compound **a2**, the unpurified product **a2** and 304mg (2.2 mmol) of K_2CO_3 were dissolved in 5 mL of MeCN. Then 0.64mL (2mmol) of CH_3I were added to the mixture and the reaction was heated up to 60°C. After 2h, the reaction was stopped, the solid was filtered off and the MeCN was evaporated under reduced pressure. Then, the

solid was recrystalized in acetone, yielding the final product **a8**. The compound was an orange solid. Yield=33%. ^{1}H NMR (400 MHz, DMSO-d6) δ 8.91 (t, J = 2.5 Hz, 1H), 8.65 (s, 2H), 4.00 (s, 2H), 3.69 (s, 2H), 3.55 (s, 2H), 3.44 (s, 2H), 3.19 (s, 6H). ^{13}C NMR (101 MHz, DMSO-d6) δ 165.18, 148.32, 137.59, 127.67, 119.60, 59.71, 50.66, 36.10. m/z calculated for 309,11935, found: 309.02 (M+H+).

Synthesis of *N*-(2-hydroxyethyl)-3,5-dinitrobenzamide (**b1**). Following the described general procedure for the amide synthesis, 543.5mg (2.4 mmol) of compound **1** was dissolved in ethyl acetate (7 mL) and was added to a solution in ethyl acetate (7 mL) of 610 mg (10mmol) of 2-aminoethan-1-ol. No K_2CO_3 was added. The compound was a yellow solid. Yield=40%. ^{1}H NMR 1H NMR (400 MHz, $CDCl_3$) δ 8.98 (dt, J = 13.9, 2.1 Hz, 3H), 3.61 (t, J = 5.2 Hz, 2H), 3.48 – 3.36 (m, 2H). ^{13}C NMR (101 MHz, $CDCl_3$): δ 163.71, 148.43, 137.71, 127.55, 120.74, 60.38, 42.75. m/z calculated for 256.05641, found: 256.08 (M+H+).

Synthesis of *N*-(3-hydroxypropyl)-3,5-dinitrobenzamide (**b2**). Following the described general procedure for the amide synthesis, 648mg (2.8 mmol) of compound **1** was dissolved in ethyl acetate (10 mL) and was added to a solution in ethyl acetate (10 mL) of 421 mg (5.6mmol) of 3-aminopropan-1-ol and 774mg (5.6mmol) of K_2CO_3. The compound was a white solid. Yield=50%. ^{1}H NMR (400 MHz, DMSO-d6): δ 9.16 (t; J = 5.4 Hz; 1H; H-8), 9.04 (m; 2H), 8.94 (t; J = 2.2 Hz; 1H), 4.51 (t; J = 5.1 Hz; 1H), 3.48 (q; J = 6.0 Hz; 2H), 3.38 (q, J = 6.7 Hz, 2H), 1.72 (p, J = 6.6 Hz, 2H; H-10). ^{13}C NMR (101 MHz, DMSOd6): δ 162.00, 148.19, 137.11, 127.46, 120.73, 58.47, 37.09, 32.08. m/z calculated for 270.07206, found: 269.99 (M+H+).

Synthesis of *N*-(5-hydroxypentyl)-3,5-dinitrobenzamide (**b3**). Following the described general procedure for the amide synthesis, 600mg (2.6 mmol) of compound **1** was dissolved in ethyl acetate (10 mL) and was added to a solution in ethyl acetate (10 mL) of 537 mg (5.2mmol) of 5-aminopentan-1-ol and 724mg (5.2mmol) of K_2CO_3. The compound was a white/yellowish solid. Yield=78%. ^{1}H NMR (400 MHz, DMSO-d6): δ 9.17 (t; J = 5.8 Hz; 1H), 9.06 (d; J = 2.1 Hz; 2H), 8.95 (t; J = 2.1 Hz; 1H), 4.39 (t; J = 5.1 Hz; 1H), 3.40 (q; J = 6.0 Hz; 2H), 3.32 (t; J = 6.4 Hz; 2H), 1.57 (p; J = 7.2 Hz; 2H), 1.46 (p; J = 6.6 Hz; 2H), 1.35 (tt; J = 8.6, 5.2 Hz; 2H). ^{13}C NMR (101 MHz, DMSO-d6): δ 161.92, 148,22, 137.10, 127.47, 120.76, 60.62, 39,79, 32.23, 28.71, 23.07. m/z calculated for 298.10336, found: 298.01 (M+H+).

Synthesis of 2-(3,5-dinitrobenzamido)ethyl 3,5-dinitrobenzoate (**c1**). Following the described general procedure for the amide synthesis, 208mg (0.9 mmol) of compound **1** was dissolved in ethyl acetate (5 mL) and was added to a solution in ethyl acetate (5 mL) of 20 mg (0.3mmol) of 2-aminoethan-1-ol and 110mg (0.8mmol) of K_2CO_3. The compound was a white solid. Yield=39%. ^{1}H NMR (300 MHz, DMSO-d6): δ 9.07-8.91 (m, 6H), 4.57 (t, J = 5.3 Hz, 2H), 3.80 (q, J=5.4 Hz, 2H).

Synthesis of 5-(3,5-dinitrobenzamido)pentyl 3,5-dinitrobenzoate (**c2**). Following the described general procedure for the amide synthesis, 208mg (0.9 mmol) of compound **1** was dissolved in ethyl acetate (5 mL) and was added to a solution in ethyl acetate (5 mL)

of 40 mg (0.4mmol) of 5-aminopentan-1-ol and 111mg (0.8mmol) of K_2CO_3. The compound was a white solid. Yield=46%. ^{1}H NMR (400 MHz, DMSO-d6) δ 9.20 (t, J = 5.6 Hz, 1H), 9.05-9.00 (m, J = 2.7, 1.2 Hz, 3H), 8.94 (t, J = 1.8 Hz, 1H), 8.88 (d, J = 2.1 Hz, 2H), 4.42 (t, J = 6.5 Hz, 2H), 3.38 (t, J = 6.3 Hz, 2H), 1.83 (p, J = 6.9 Hz, 2H), 1.66 (p, J = 7.2 Hz, 2H), 1.55 – 1.44 (m, 2H). ^{13}C NMR (101 MHz, DMSO-d6) δ 162.67, 162.05, 148.45, 148.26, 137.13, 132.81, 128.87, 127.51, 122.57, 120.82, 66.35, 39.55, 28.44, 27.73, 22.91. m/z calculated for 492.09973, found: 492.06 (M+H+).

Synthesis of product *N*-(4-methoxyphenyl)-3,5-dinitrobenzamide (**f1**). Following the described general procedure for the amide synthesis, compound **1** was dissolved in ethyl acetate and was added to a solution of 4-methoxyaniline and K_2CO_3. The compound was a yellow solid. Yield=31%. ^{1}H NMR (400 MHz, DMSO-d6) δ 10.74 (s, 1H), 9.15 (t, J = 1.9 Hz, 2H), 8.97 (s, 1H), 7.68 (d, J = 7.2 Hz, 2H), 6.96 (d, J = 7.3 Hz, 2H), 3.76 (s, 3H). ^{13}C NMR (101 MHz, DMSO-d6) δ 160.78, 156.16, 148.15, 137.52, 131.30, 127.93, 122.31, 120.99, 113.91, 55.24, 30.73. m/z calculated for 318.07206, found: 318.01 (M+H+).

S1.3. Mitsunobu reaction:

General procedure: A solution of the appropriate product (1 eq.), the respective phenol (1 eq.) and ADDP (2.5 eq.) in dichloromethane was placed under an atmosphere of nitrogen and for 10-15 min is purged with nitrogen. Then, to this mixture, a solution of PPh_3 (2.5 eq.) in dichloromethane was added dropwise, over a 2min period, and the reaction was stirred at room temperature for around 24 hours, all while keeping the reaction under a nitrogen atmosphere. After this, the DCM was evaporated, the mixture was dissolved in EtOAc and washed successively with 10 mL of distilled water and with 10 mL of brine. The ethyl acetate solution was subsequently dried, and the solvent evaporated. The residue was purified by column chromatography (silica gel 60) using hexane: ethyl acetate, 8:2 to 1:1 as eluent.

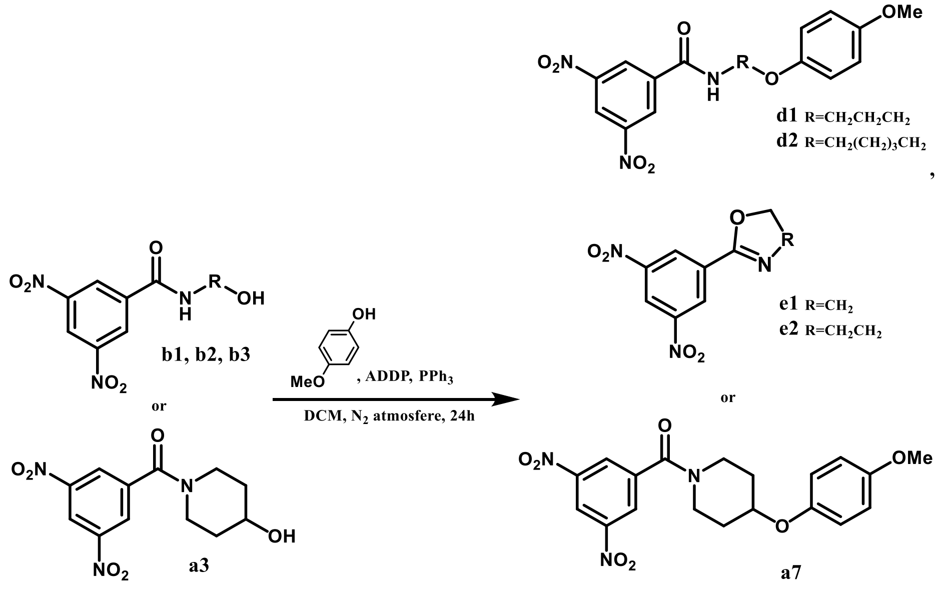

Figure S1.3: General synthetic scheme for the Mitsunobu reaction

Synthesis of (3,5-dinitrophenyl)(4-(4-methoxyphenoxy)piperidin-1-yl)methanone (**a7**). Following the described general procedure for the Mitsunobu reaction, 224mg (0.85mmol) of PPh_3 in 2mL of DCM was added to a solution in 2mL of DCM of 42mg (0.34mmol) of 4-methoxyphenol, 215mg (0.85mmol) of ADDP and 100mg (0.34mmol) of **a3**. The compound was a yellow solid. Yield=29%. ^{1}H NMR (400 MHz, $CDCl_3$) δ 9.09 (t, J = 1.4 Hz, 1H), 8.64 – 8.57 (m, 2H), 6.85 (q, J = 9.2 Hz, 4H), 4.52 (p, J = 4.6 Hz, 1H), 3.98 (s, 1H), 3.84 (s, 1H), 3.77 (d, J = 1.0 Hz, 3H), 3.69 (s, 1H), 3.37 (s, 1H), 2.01 (s, 1H), 1.87 (s, 2H). ^{13}C NMR (101 MHz, $CDCl_3$) δ 162.85, 159.15, 148.72, 138.26, 129.54, 127.27, 121.11, 120.61, 114.55, 67.90, 40.96, 29.55, 29.37, 27.05, 26.13. m/z calculated for 402.12958, found: 402.02 (M+H+).

Synthesis of *N*-(3-(4-methoxyphenoxy)propyl)-3,5-dinitrobenzamide (**d1**). Following the described general procedure for the Mitsunobu reaction, 223mg (0.85mmol) of PPh_3 in 2mL of DCM was added to a solution in 2mL of DCM of 42mg (0.34mmol) of 4-methoxyphenol, 215mg (0.85mmol) of ADDP and 92mg (0.32mmol) of **b2**. In this reaction a secondary product also formed (**e2**), and to separate both compounds a second column was required (eluent 100% toluene to 96% toluene /4% EtOAc). The compound was an orange solid. Yield=42%. ^{1}H NMR (400 MHz, $CDCl_3$) δ 9.09 (t, J = 2.2 Hz, 1H), 8.99 (d, J = 2.1 Hz, 2H), 6.84 – 6.74 (q, J = 6.40, 6.29, 6.29 Hz, 4H), 4.04 (t, J = 5.7 Hz, 2H), 3.71 (s, 3H), 3.64 (t, J = 6.5 Hz, 2H), 2.09 (p, J = 6.1 Hz, 2H). ^{13}C NMR (101 MHz, $CDCl_3$) δ 163.30, 154.42, 152.83, 148.90, 138.32, 127.78, 121.24, 115.66, 115.14, 67.43, 56.09, 38.95, 28.93. m/z calculated for 376.11393, found: 375.97 (M+H+).

Synthesis of *N*-(5-(4-methoxyphenoxy)pentyl)-3,5-dinitrobenzamide (**d2**). Following the described general procedure for the Mitsunobu reaction, 223mg (0.85mmol) of PPh_3 in 2mL of DCM was added to a solution in 2mL of DCM of 42mg (0.34mmol) of 4-methoxyphenol, 215mg (0.85mmol) of ADDP and 100mg (0.34mmol) of **b3**. The compound was an orange/yellowish solid. Yield=43%. ^{1}H NMR (400 MHz, $CDCl_3$) δ 9.14 (s, 1H), 8.94 (t, J = 1.6 Hz, 2H), 6.80 (s, 4H), 6.63 (br s, 1H), 3.93 (t, J = 6.1 Hz, 2H), 3.75 (s, 3H), 3.57 (q, J = 6.6 Hz, 2H), 1.83 (p, J = 6.6 Hz, 2H), 1.75 (q, J = 7.4 Hz, 2H), 1.61 (q, J = 7.8 Hz, 2H). ^{13}C NMR: (101 MHz, $CDCl_3$) δ 162.89, 153.91, 148.72, 138.19, 127.27, 121.12, 115.48, 114.76, 68.33, 40.82, 29.16, 29.01, 23.75. m/z calculated for 404.14523, found: 404.11 (M+H+).

Synthesis of 2-(3,5-dinitrophenyl)-4,5-dihydrooxazole (**e1**). Trying to react **b1** with 4-methoxyphenol using the described general procedure for the Mitsunobu reaction led to the formation of only the intramolecular cyclization product. For this, 224mg (0.85mmol) of PPh_3 in 2mL of DCM was added to a solution in 2mL of DCM of 42mg (0.34mmol) of 4-methoxyphenol, 215mg (0.85mmol) of ADDP and 82mg (0.34mmol) of **b1**. The compound was a white-yellowish solid. Yield=70%. ^{1}H NMR (400 MHz, DMSO-d6): δ 8.88 (m; 1H), 8.75 (m; 2H), 4.49 (t; J = 9.6 Hz; 2H), 4.01 (t; J = 9.6 Hz; 2H). ^{13}C NMR (101 MHz, DMSO-d6): δ 160.19, 148.41, 130.18, 127.41, 120.95, 68.83, 54.86. m/z calculated for 238.04585, found: 238.02 (M+H+).

Synthesis of 2-(3,5-dinitrophenyl)-5,6-dihydro-4H-1,3-oxazine (**e2**). This compound was isolated from the same reaction of the synthesis of **d1**. Yield=15%. ^{1}H NMR (400 MHz,

DMSO-d6): δ 8.89 (t; J = 2.2 Hz; 1H), 8.83 (d; J = 2.2 Hz; 2H), 4.45 (t; J = 5.4 Hz; 2H), 3.59 (t; J = 5.8 Hz; 2H), 1.95 (p; J = 5.6 Hz; 2H). ^{13}C NMR (101 MHz, DMSO-d6): δ 150.99, 148.21, 136.61, 126.14, 119.93, 65.79, 42.23, 21.18. m/z calculated for 252.06150, found: 252.01 (M+H+).

S1.4. Diamide formation:

General procedure of the first step: A solution of appropriate product in a mixture of thionyl chloride, toluene and DMF (1/1/0.1, in a total volume of 3 mL per mmol of reactant), was refluxed for 12 h, leading to the formation of the desired product. The excess solvent mixture and thionyl chloride were removed by low pressure evaporation. Then, the mixture was dissolved in EtOAc and washed successively with 10 mL of distilled water and with 10 mL of brine. The ethyl acetate solution was subsequently dried, and the solvent evaporated. The residue was purified by column chromatography (silica gel 60) using hexane: ethyl acetate, 8:2 to 1:1 as eluent.

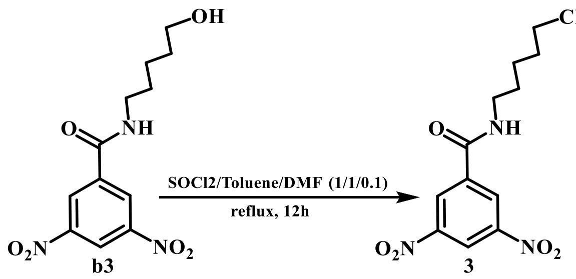

Figure S1.4: General synthetic scheme for the first step of the diamide formation

Synthesis of *N*-(5-chloropentyl)-3,5-dinitrobenzamide (**3**). Following the first step described in the general procedure for the diamide formation, 298mg (1mmol) of **b3** was dissolved in toluene/$SOCl_2$ (1/1 in volume, total volume 2,9mL), and 0,1 mL of DMF was added to the reaction mixture. The compound was a white solid. Yield=97%. ^{1}H NMR (400 MHz, $CDCl_3$) δ 9.16 (t, J = 2.2 Hz, 1H), 8.96 (d, J = 2.1 Hz, 2H), 6.60 (t, J = 5.8 Hz, 1H), 3.56 (dt, J = 9.0, 6.5 Hz, 4H), 1.85 (p, J = 6.7 Hz, 2H), 1.72 (p, J = 7.3 Hz, 2H), 1.58 (tt, J = 9.7, 5.8 Hz, 2H). ^{13}C NMR (101 MHz, $CDCl_3$) δ 162.93, 148.77, 138.13, 127.76, 121.21, 44.91, 40.68, 32.05, 28.81, 24.25.

General procedure of the second step: A solution of the appropriate product, NaN_3 (3 eq.) and NaI (0,3 eq.) in MeCN were heated at 70ºC and stirred for 5 days. The MeCN was then removed by low pressure evaporation and the mixture was dissolved in EtOAc and washed successively with 10 mL of distilled water and with 10 mL of brine. The ethyl acetate solution was subsequently dried, and the solvent evaporated. The residue was purified by column chromatography (silica gel 60) using hexane: ethyl acetate, 8:2 to 1:1 as eluent.

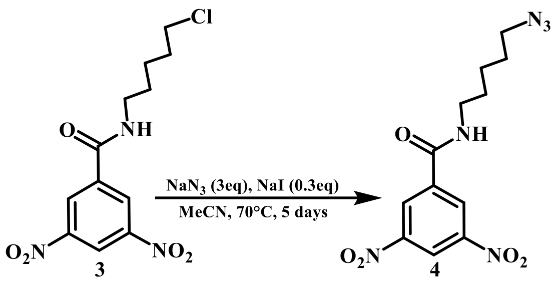

Figure S1.5: General synthetic scheme for the second step of the diamide formation

Synthesis of *N*-(5-azidopentyl)-3,5-dinitrobenzamide (**4**). Following the second step described in the general procedure for the diamide formation, 316mg (0.97mmol) of compound **3**, 45mg (0.3mmol) of NaI and 195mg (3mmol) of NaN_3 were dissolved in 10mL of MeCN. The compound was a yellow oil. Mass of product obtained: 311mg. Yield=97%. ^{1}H NMR (400 MHz, $CDCl_3$) δ 9.18 (t, J = 2.0 Hz, 1H), 8.96 (dd, J = 2.2, 1.0 Hz, 2H), 6.48 (br s, 1H), 3.56 (q, J = 6.8 Hz, 2H), 3.33 (t, J = 6.6 Hz, 2H), 1.70 (dp, J = 18.0, 7.7 Hz, 4H), 1.51 (p, J = 7.7 Hz, 2H).

General procedure of the third step: A solution of the appropriate product and PPh_3 (2 eq.) were dissolved in THF, under a N_2 atmosphere, and was stirred for 24h. Then, a small quantity of distilled water is added to the mixture and the reaction is stirred for another 24h. The solvent is then evaporated, and the product used without further purification.

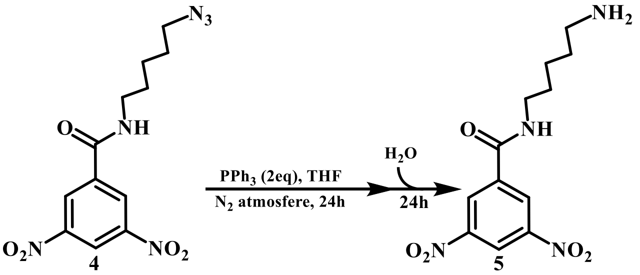

Figure S1.6: General synthetic scheme for the third step of the diamide formation

Synthesis of *N*-(5-aminopentyl)-3,5-dinitrobenzamide (**5**). Following the third step described in the general procedure for the diamide formation, 0.94mmol of compound **4** and 500mg (1.9mmol) of PPh_3 were dissolved in 6mL of THF. After 24h, 0.1mL of distilled water was added to the mixture, and the reaction was stirred for another 24h. After the end of the reaction, the solvent was evaporated and the product used without further purification.

Formation of the final compounds:

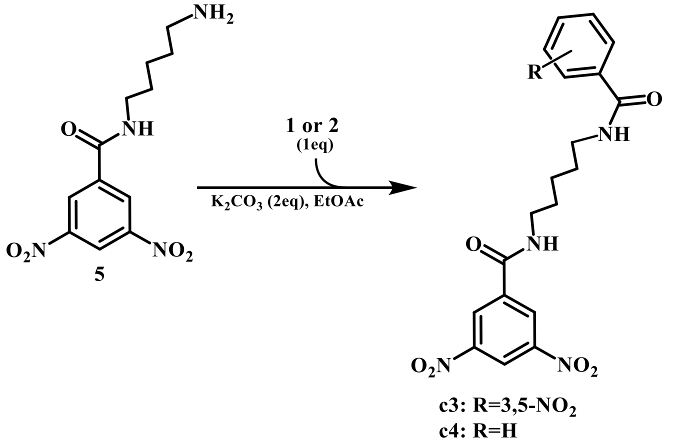

Figure S1.7: General synthetic scheme for the final step of the diamide formation

Synthesis of *N,N'*-(pentane-1,5-diyl)bis(3,5-dinitrobenzamide) (**c3**). Following the described general procedure for the amide synthesis (S1.2.), 217mg (0.94mmol) of compound **1** dissolved in ethyl acetate (10 mL) was added to a solution in ethyl acetate (15 mL) of 278 mg (0.94mmol) of compound **5** and 304mg (2.2mmol) of K_2CO_3. The compound was a white solid. Yield=14%. ^{1}H NMR (400 MHz, DMSO-d6): δ 9.19 (t; J = 5.6 Hz; 2H), 9.03 (d; J = 2.0 Hz; 4H), 8.94 (t; J = 2.1 Hz; 2H), 3.33 (d; J = 5.9 Hz; 4H), 1.62 (p; J = 7.2 Hz; 4H), 1.41 (d; J = 7.2 Hz; 2H). ^{13}C NMR (101 MHz, DMSO-d6) δ 162.67, 162.05, 148.45, 148.26, 137.13, 132.81, 128.87, 127.51, 122.57, 120.82, 66.35, 39.55, 28.44, 27.73, 22.91. m/z calculated for 491.11572, found: 491.01 (M+H+).

Synthesis of *N*-(5-benzamidopentyl)-3,5-dinitrobenzamide) (**c4**). Following the described general procedure for the amide synthesis (S1.1.), 61mg (0.5mmol) of compound **2** dissolved in ethyl acetate (7 mL) was added to a solution in ethyl acetate (10 mL) of 148 mg (0.5mmol) of compound **5** and 138mg (1mmol) of K_2CO_3. The compound was a white solid. Yield=55%. ^{1}H NMR (400 MHz, $CDCl_3$) δ 9.04 (s, 3H), 7.55 (m, 3H), 7.44 (t, J = 7.1 Hz, 1H), 7.32 (t, J = 7.6 Hz, 2H), 6.28 (s, 1H), 3.54 (m, 4H), 1.81 (p, J = 6.6 Hz, 2H), 1.69 (p, J = 6.6 Hz, 2H), 1.50 (p, J = 6.9 Hz, 2H). ^{13}C NMR (101 MHz, DMSO-d6) δ 169.38, 163.45, 150.83, 131.76, 128.73, 127.73, 126.63, 120.79, 40.45, 38.83, 29.67, 27.59, 23.18. m/z calculated for 401.14556, found: 401.17 (M+H+).

S2. Structure of the compounds:

Table S2.1: Structure of the compounds and their respective names

Compound	Compound Structure	Name
**1**	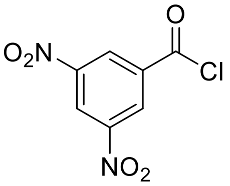	3,5-dinitrobenzoyl chloride
**2**	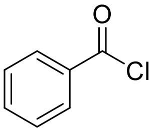	benzoyl chloride
**3**	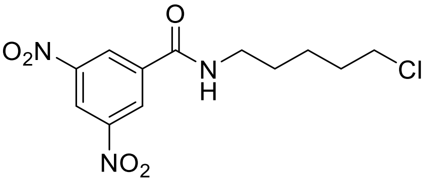	*N*-(5-chloropentyl)-3,5-dinitrobenzamide
**4**	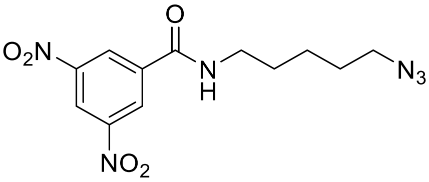	*N*-(5-azidopentyl)-3,5-dinitrobenzamide
**5**	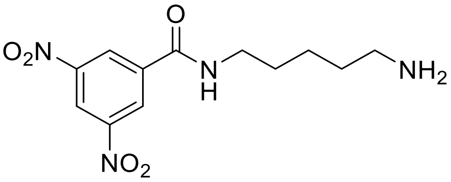	*N*-(5-aminopentyl)-3,5-dinitrobenzamide
**a1**	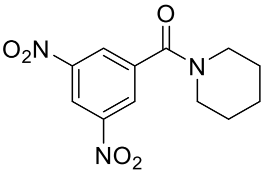	(3,5-dinitrophenyl)-piperidin-1-yl-methanone
**a2**	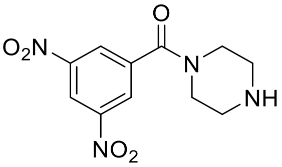	(3,5-dinitrophenyl)-piperazin-1-yl-methanone
**a3**	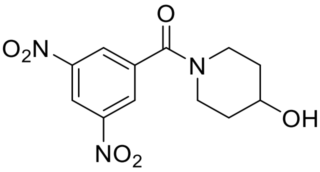	(3,5-dinitrophenyl)-4-hydroxypiperidin-1-yl-methanone
**a4**	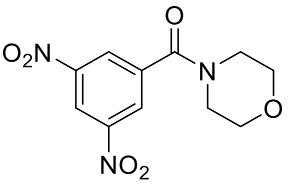	(3,5-dinitrophenyl)(morpholino)-methanone
Continues on the next page		

Compound	Compound Structure	Name
**a5**	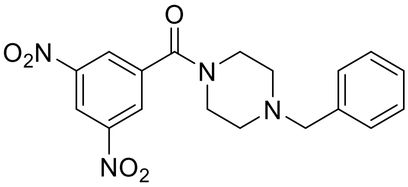	(3,5-dinitrophenyl)-4-benzylpiperazin-1-yl-methanone
**a6**	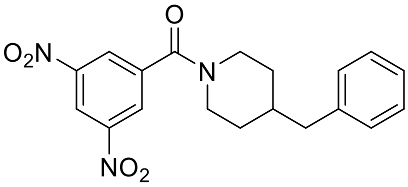	(3,5-dinitrophenyl)-4-benzylpiperidin-1-yl-methanone
**a7**	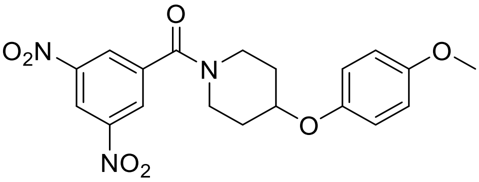	(3,5-dinitrophenyl)(4-(4-methoxyphenoxy)piperidin-1-yl)methanone
**a8**	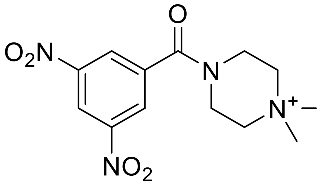	4-(3,5-dinitrobenzoyl)-1,1-dimethylpiperazin-1-ium
**b1**	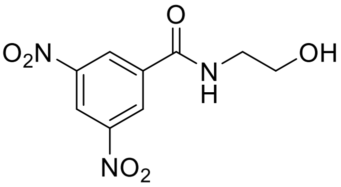	*N*-(2-hydroxyethyl)-3,5-dinitrobenzamide
**b2**	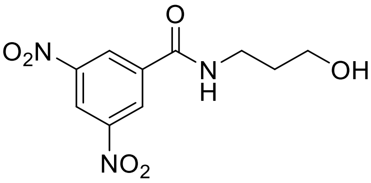	*N*-(3-hydroxypropyl)-3,5-dinitrobenzamide
**b3**	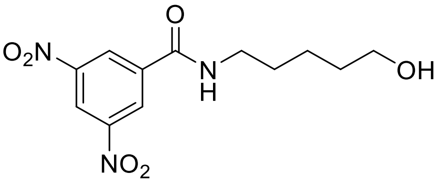	*N*-(5-hydroxypentyl)-3,5-dinitrobenzamide
**c1**	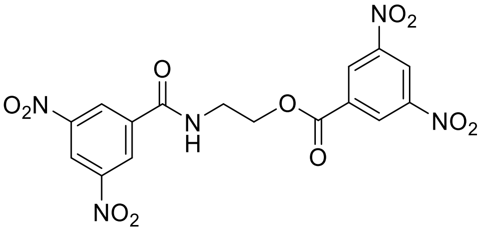	2-(3,5-dinitrobenzamido)ethyl 3,5-dinitrobenzoate
**c2**	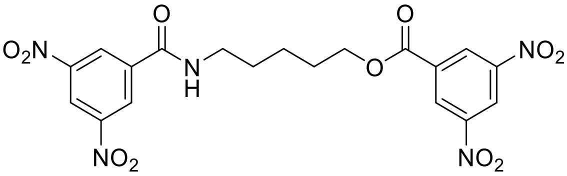	5-(3,5-dinitrobenzamido)pentyl 3,5-dinitrobenzoate

Continues on the next page

Compound	Compound Structure	Name
**c3**	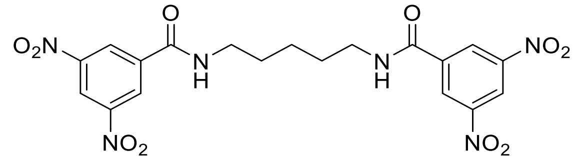	*N,N′*-(pentane-1,5-diyl)bis(3,5-dinitrobenzamide)
**c4**	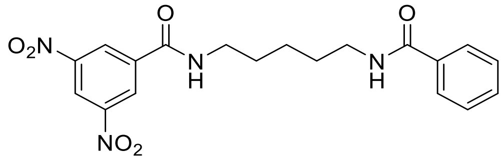	*N*-(5-benzamidopentyl)-3,5-dinitrobenzamide)
**d1**	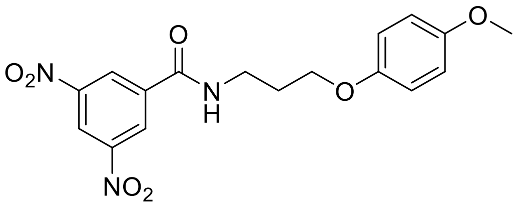	*N*-(3-(4-methoxyphenoxy)propyl)-3,5-dinitrobenzamide
**d2**	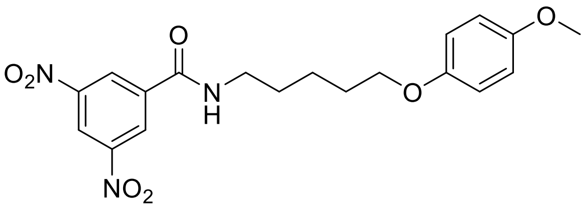	*N*-(5-(4-methoxyphenoxy)pentyl)-3,5-dinitrobenzamide
**e1**	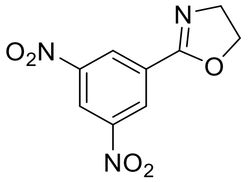	2-(3,5-dinitrophenyl)-4,5-dihydrooxazole
**e2**	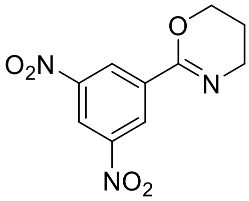	2-(3,5-dinitrophenyl)-5,6-dihydro-4H-1,3-oxazine
**f1**	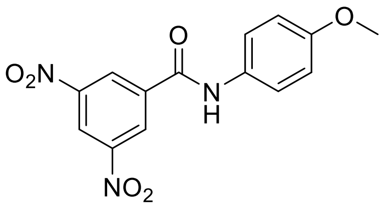	*N*-(4-methoxyphenyl)-3,5-dinitrobenzamide
DNB1	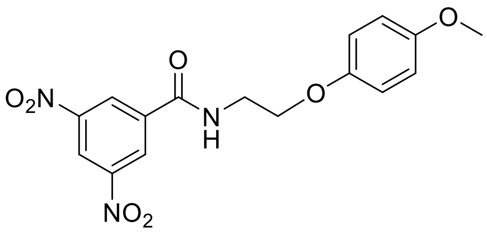	*N*-(2-(4-methoxyphenoxy)ethyl)-3,5-dinitrobenzamide

S3. ^{1}H and ^{13}C NMR spectra:

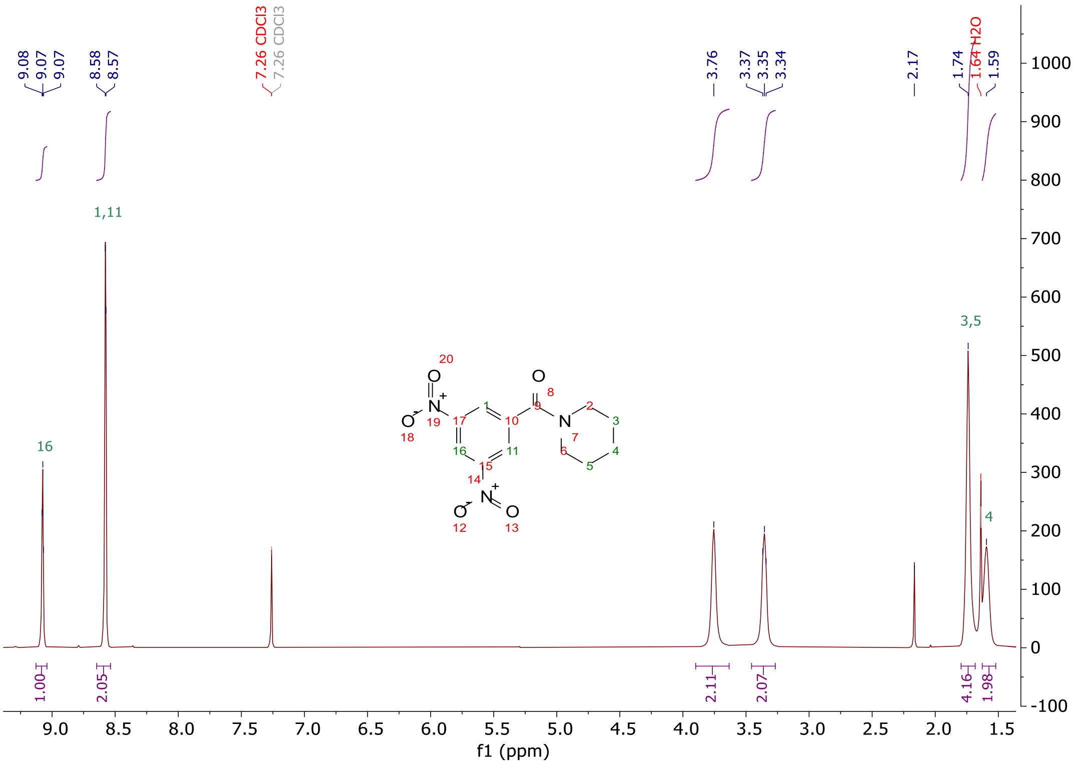

Figure S3.1: ^{1}H NMR (400 MHz, $CDCl_3$) spectrum of **a1**

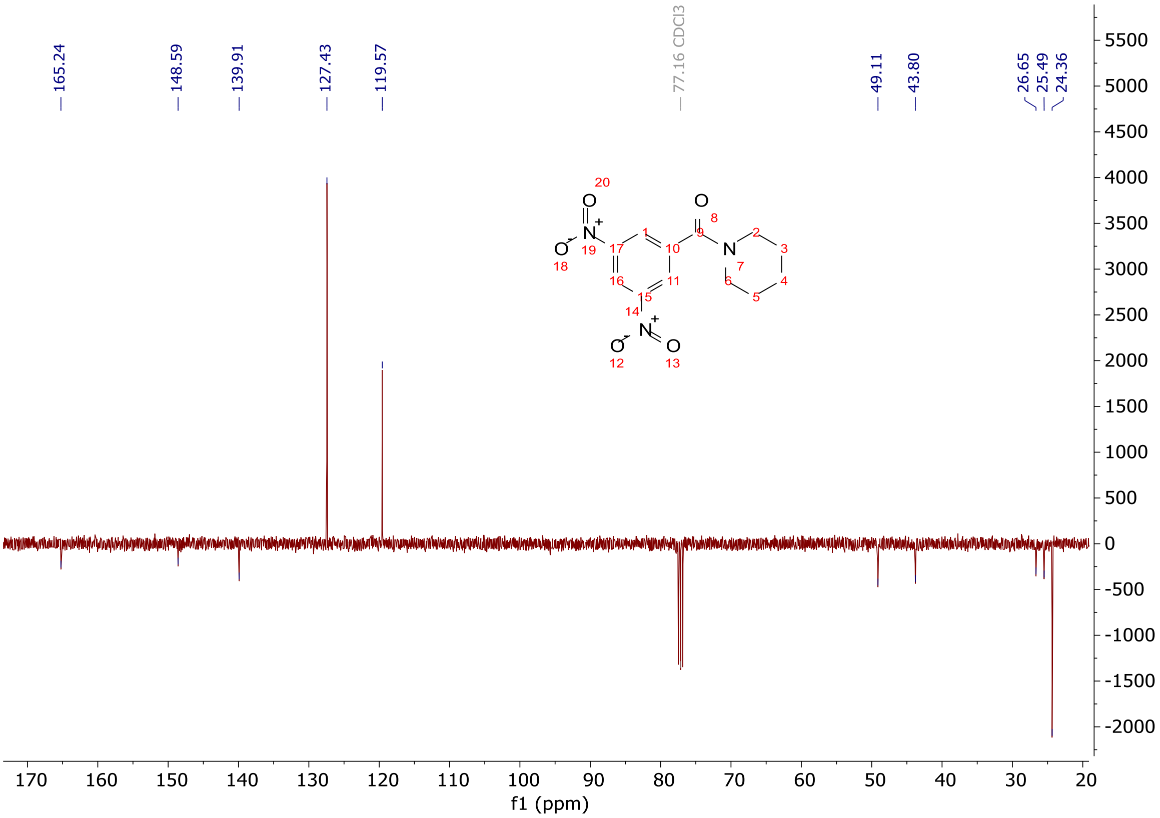

Figure S3.2: ^{13}C-APT NMR (101 MHz, $CDCl_3$) spectrum of **a1**

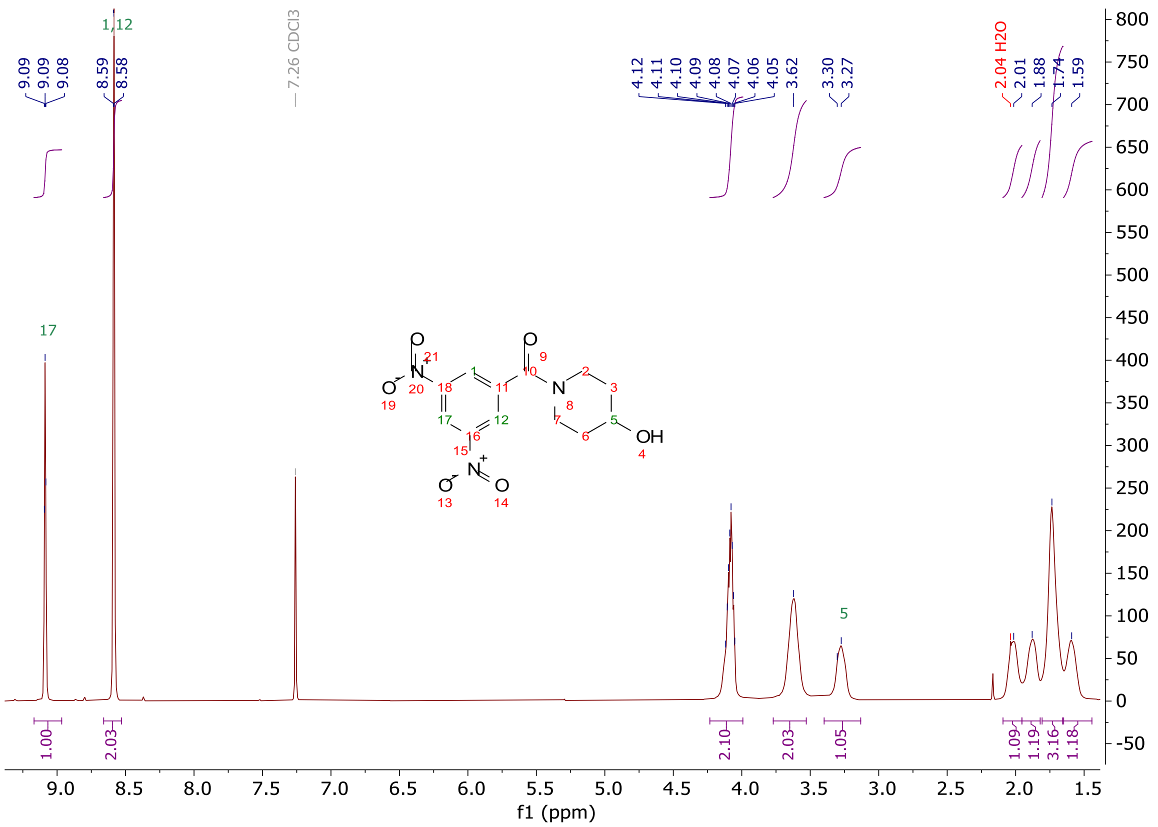

Figure S3.3: ^{1}H NMR (400 MHz, $CDCl_3$) spectrum of **a3**

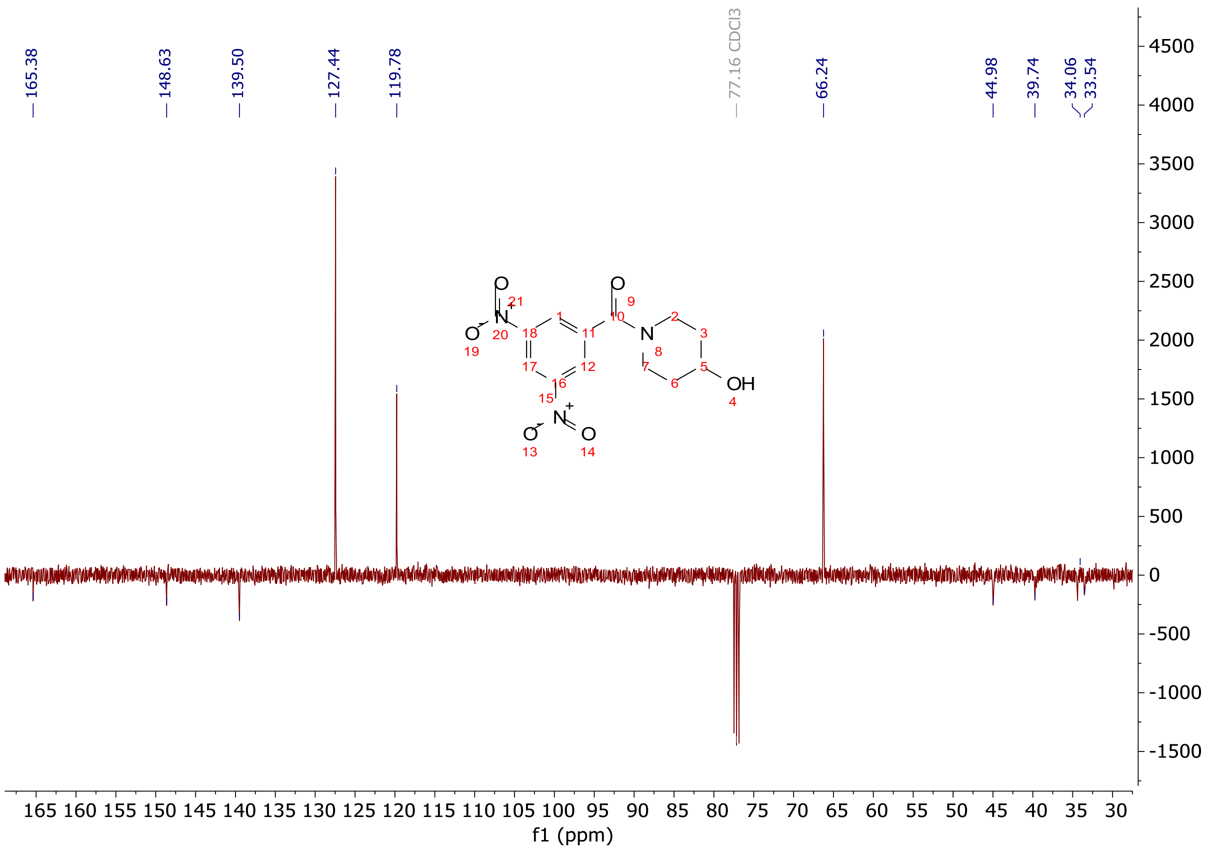

Figure S3.4: ^{13}C-APT NMR (101 MHz, $CDCl_3$) spectrum of **a3**

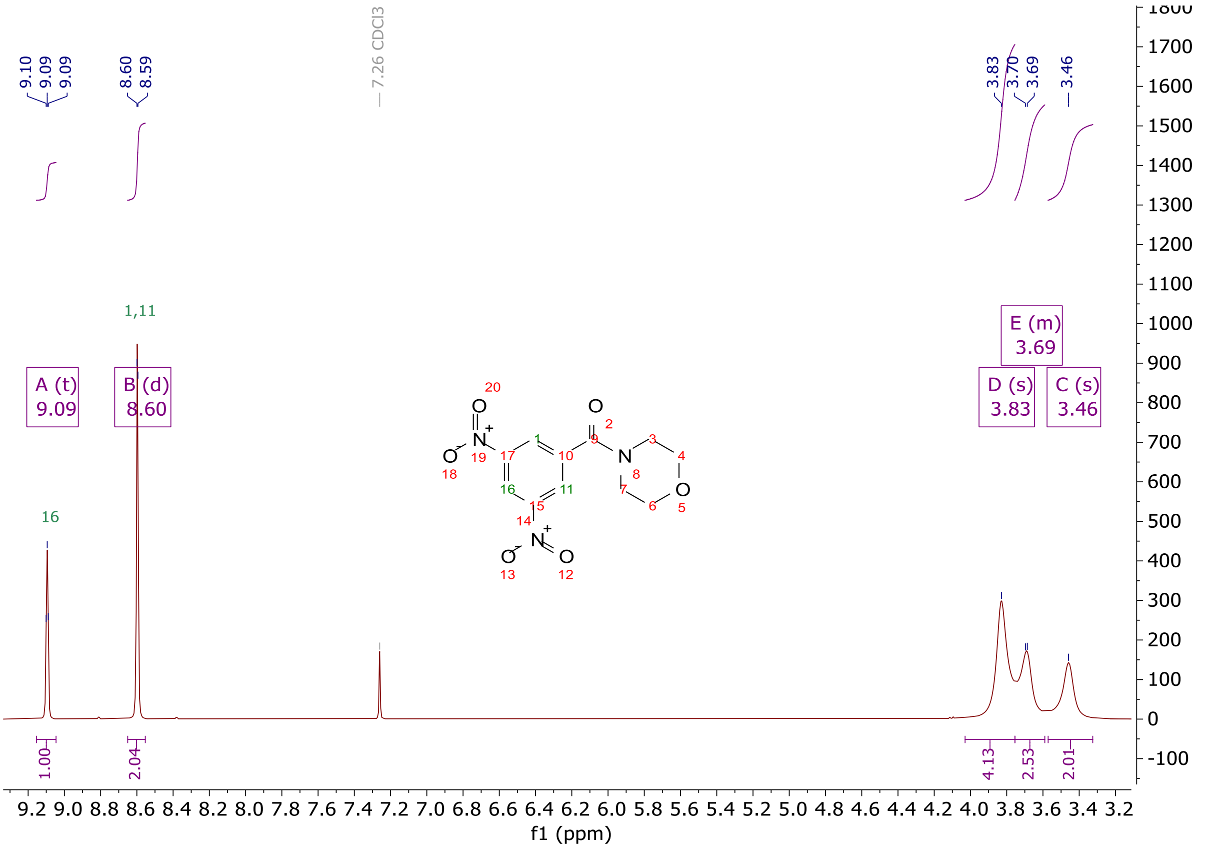

Figure S3.5: ^{1}H NMR (400 MHz, $CDCl_3$) spectrum of **a4**

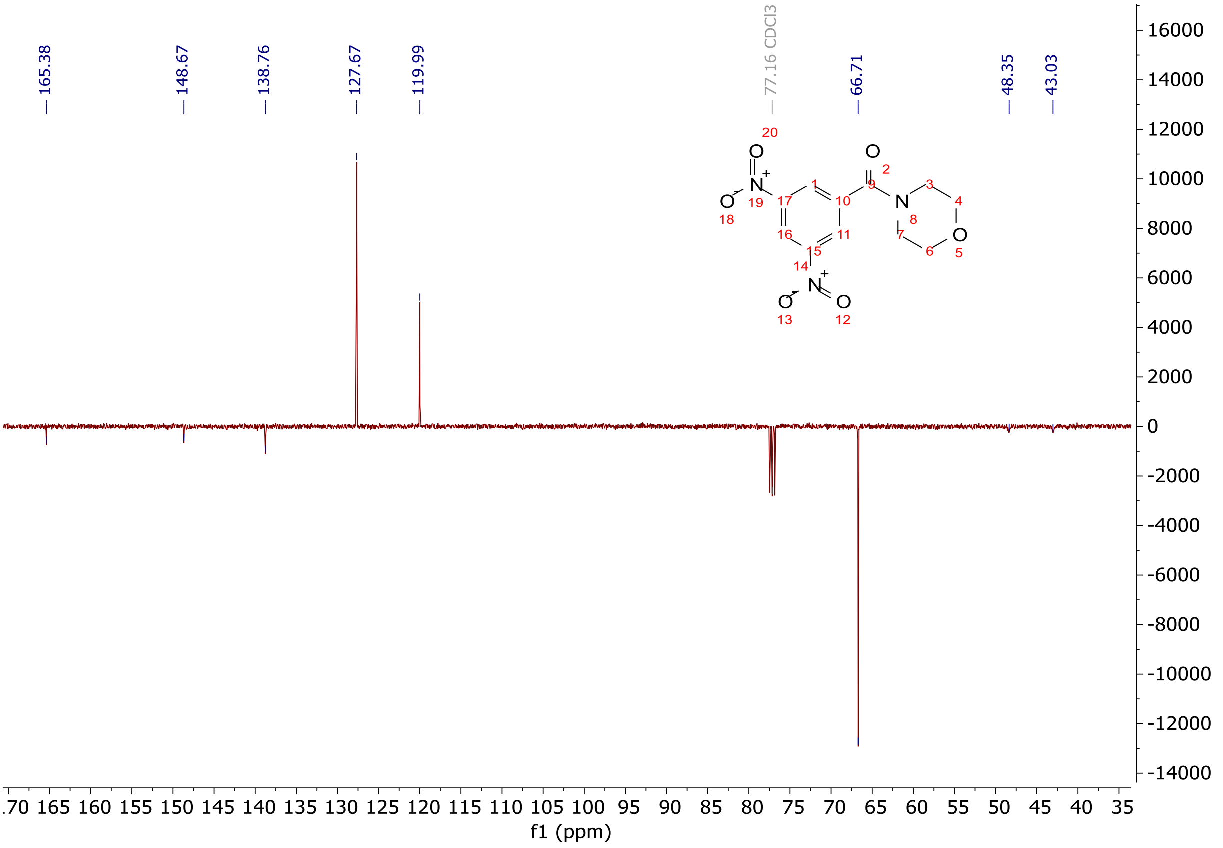

Figure S3.6: ^{13}C-APT NMR (101 MHz, $CDCl_3$) spectrum of **a4**

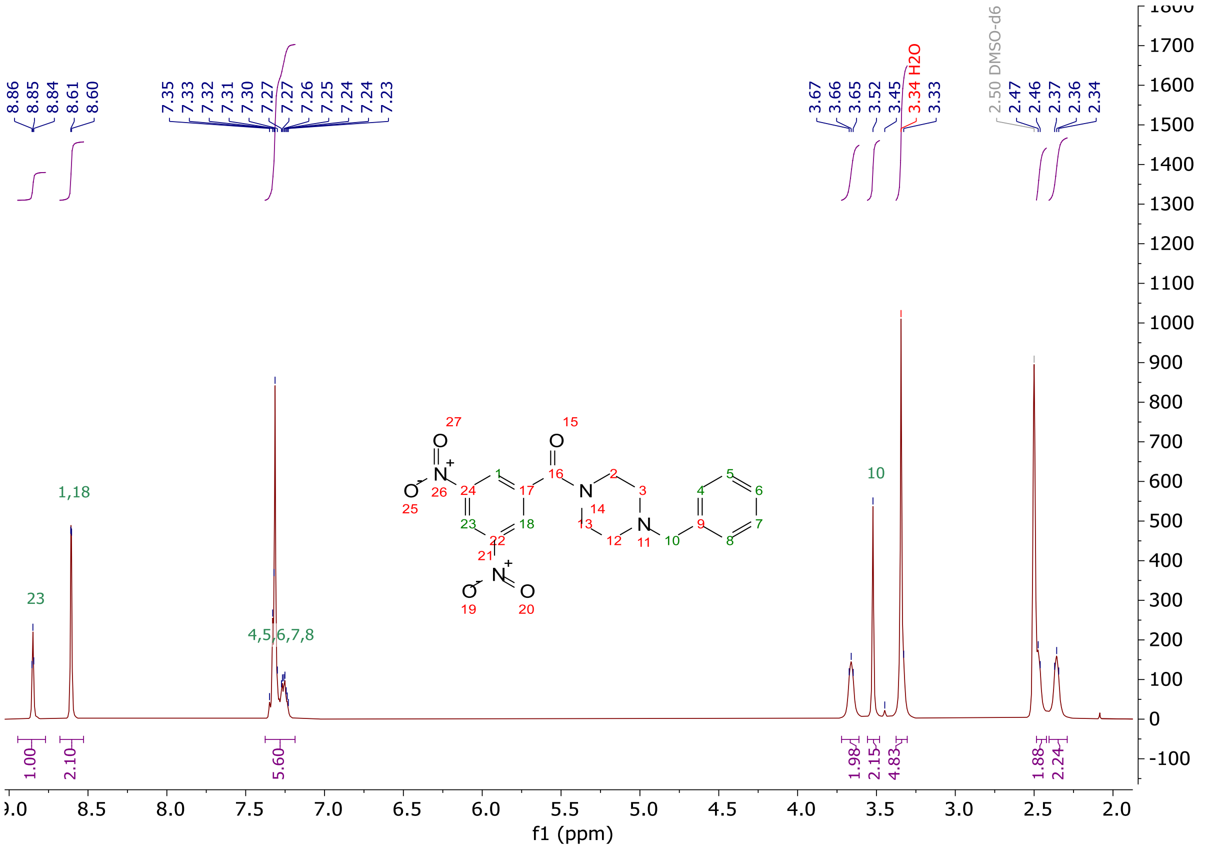

Figure S3.7: ^{1}H NMR (400 MHz, DMSO) spectrum of **a5**

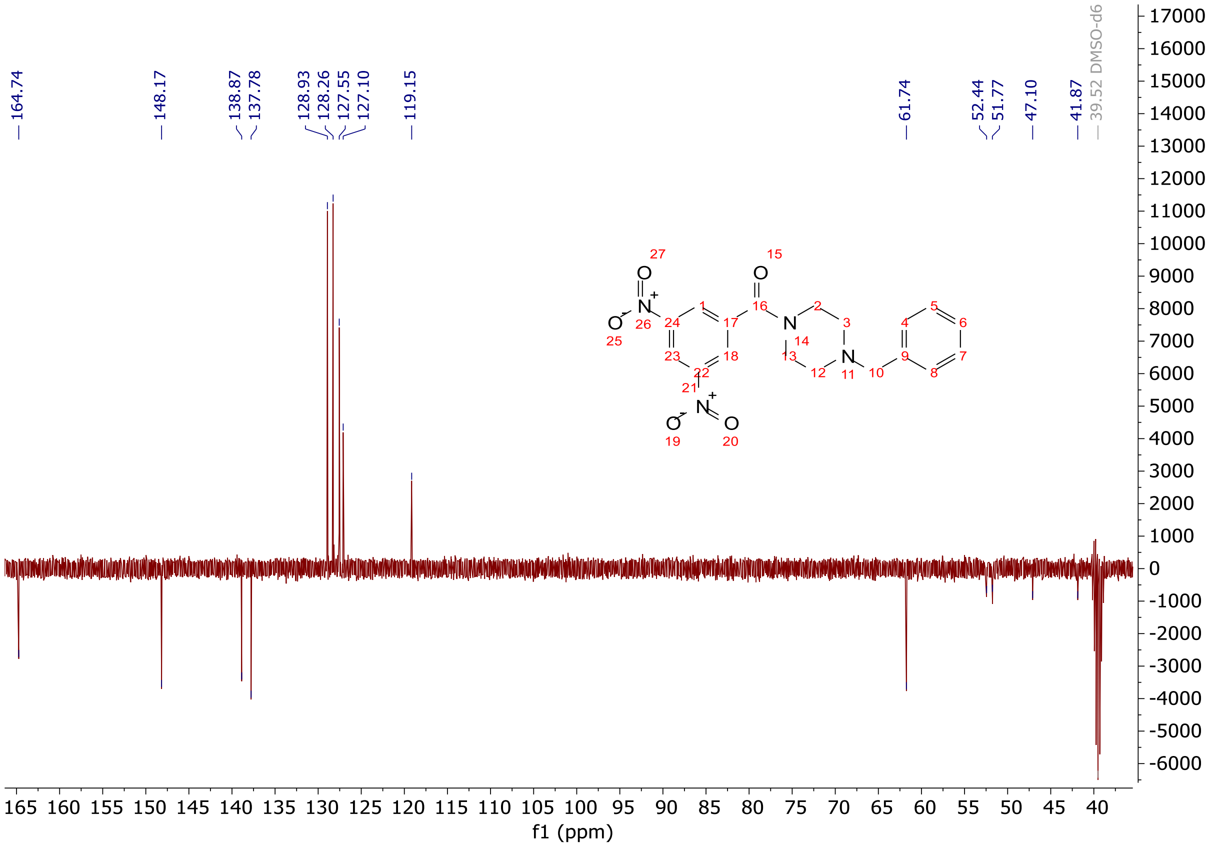

Figure S3.8: ^{13}C-APT NMR (101 MHz, DMSO) spectrum of **a5**

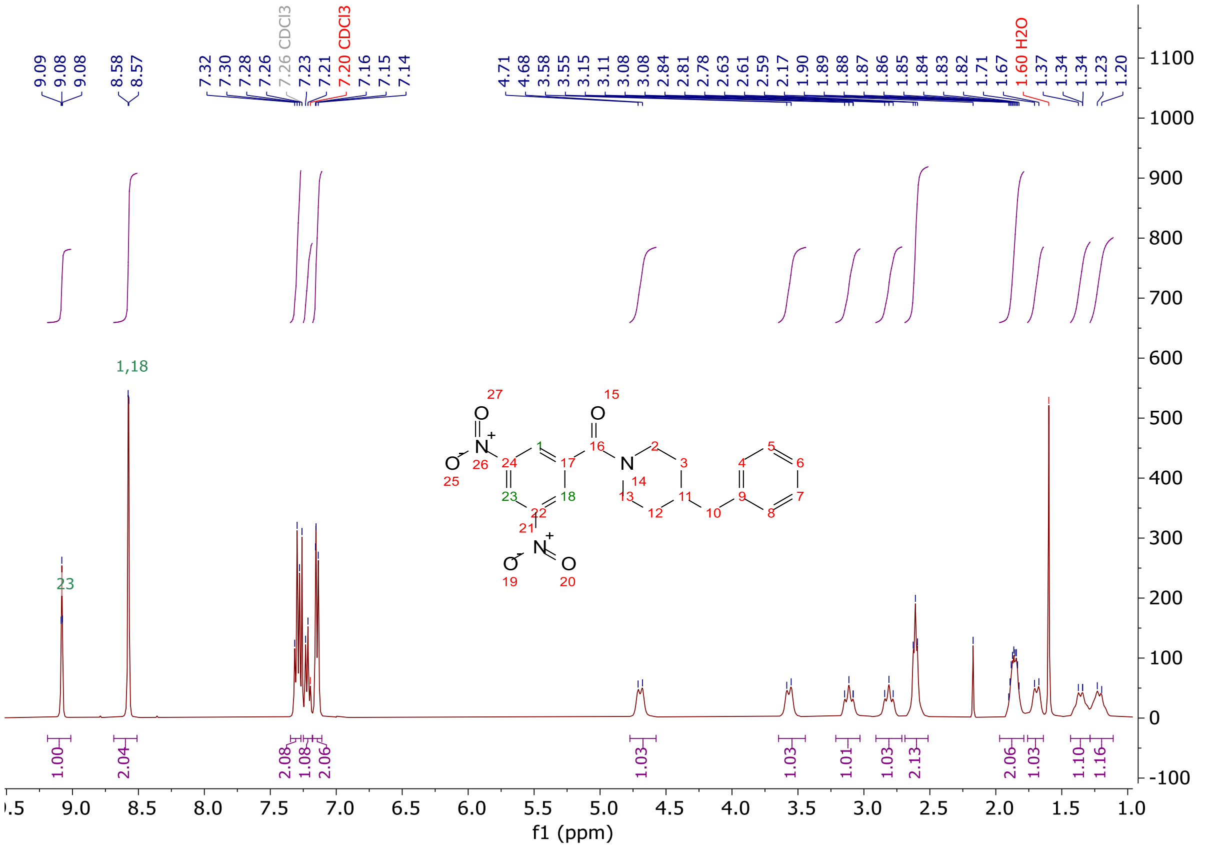

Figure S3.9: ^{1}H NMR (400 MHz, $CDCl_3$) spectrum of **a6**

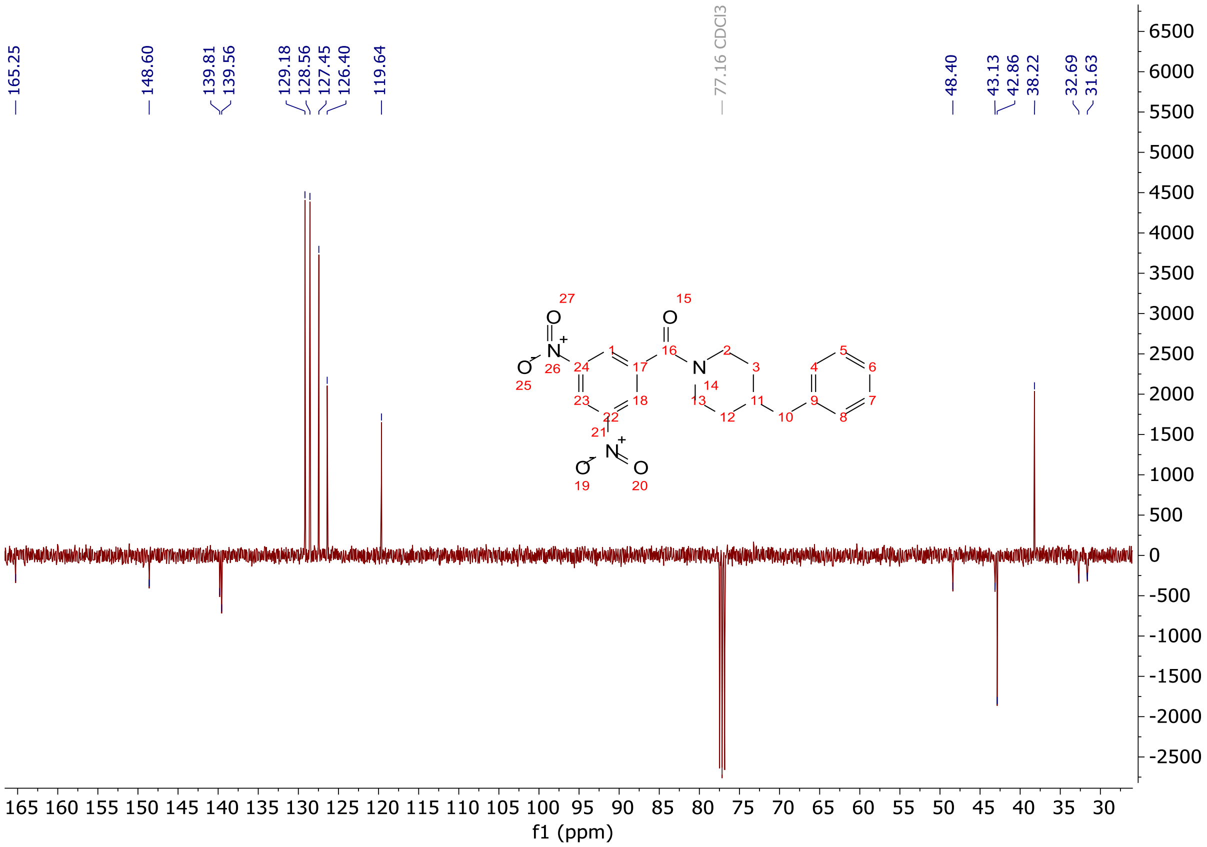

Figure S3.10: ^{13}C-APT NMR (101 MHz, $CDCl_3$) spectrum of **a6**

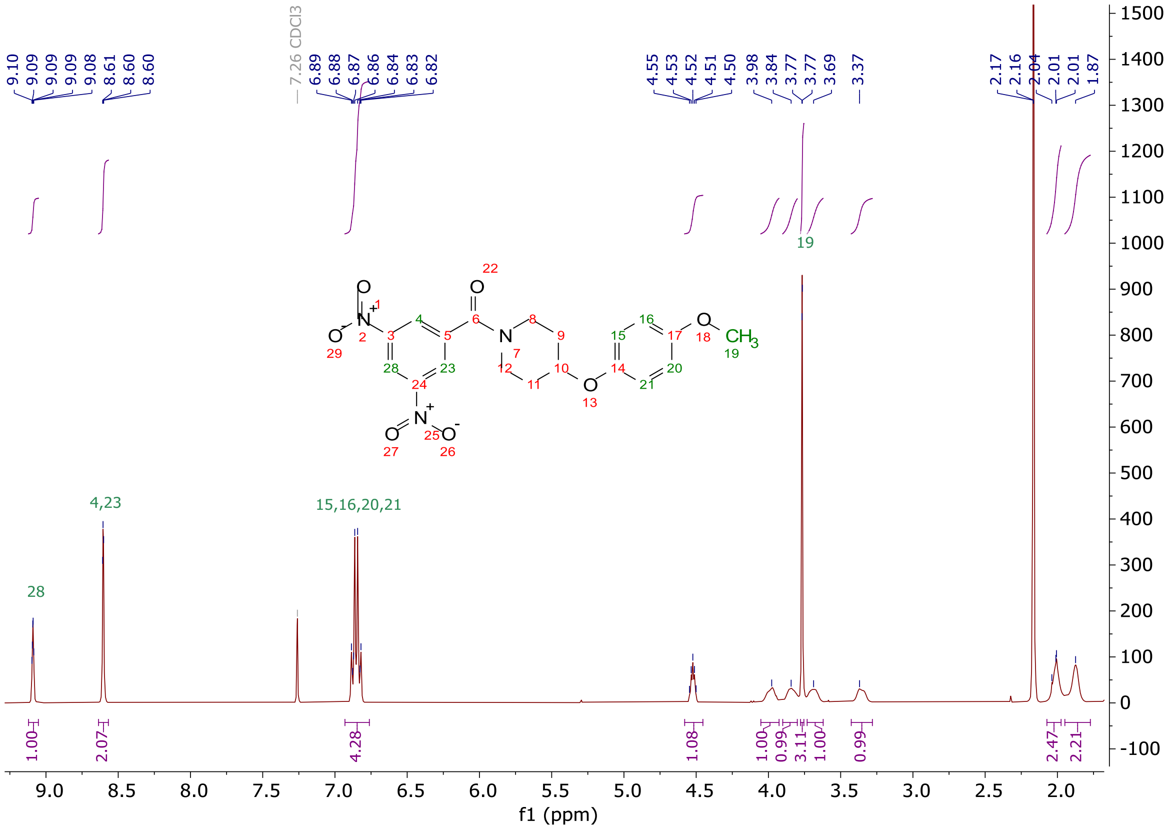

Figure S3.11: ^{1}H NMR (400 MHz, $CDCl_3$) spectrum of **a7**

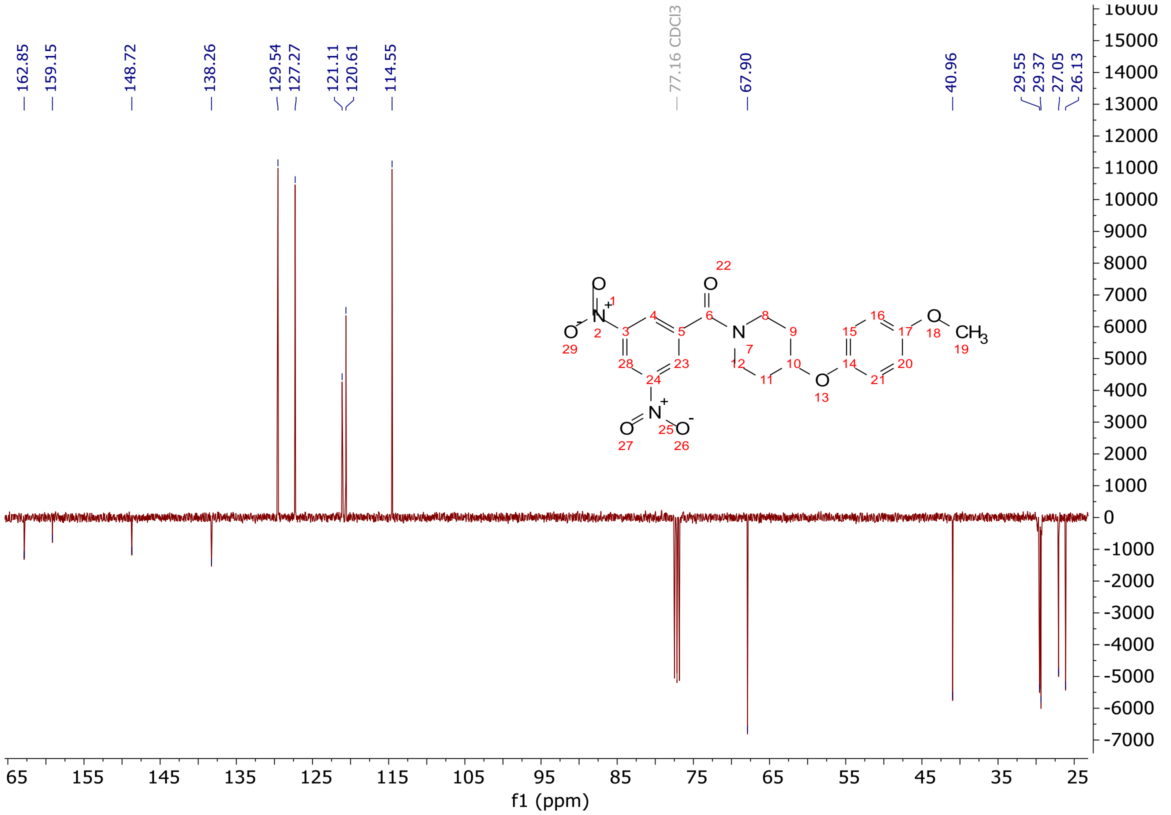

Figure S3.12: ^{13}C-APT NMR (101 MHz, $CDCl_3$) spectrum of **a7**

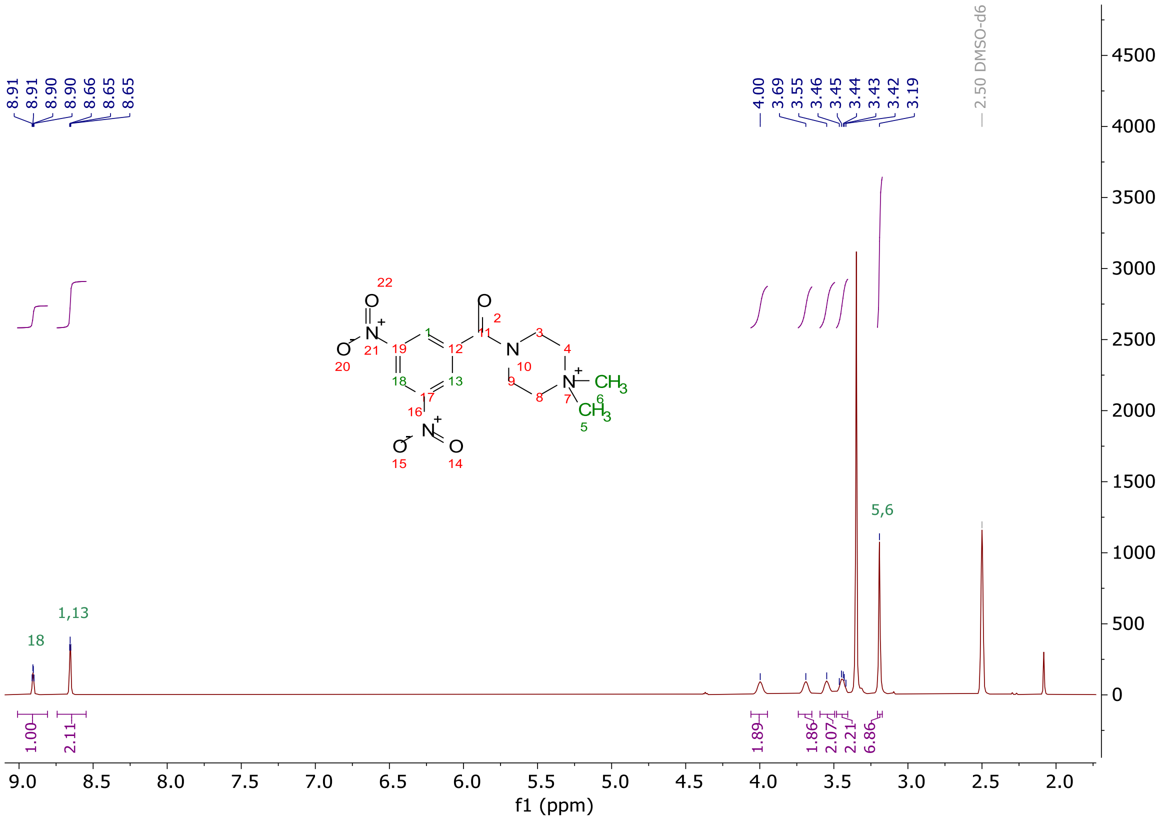

Figure S3.13: ^{1}H NMR (400 MHz, DMSO) spectrum of **a8**

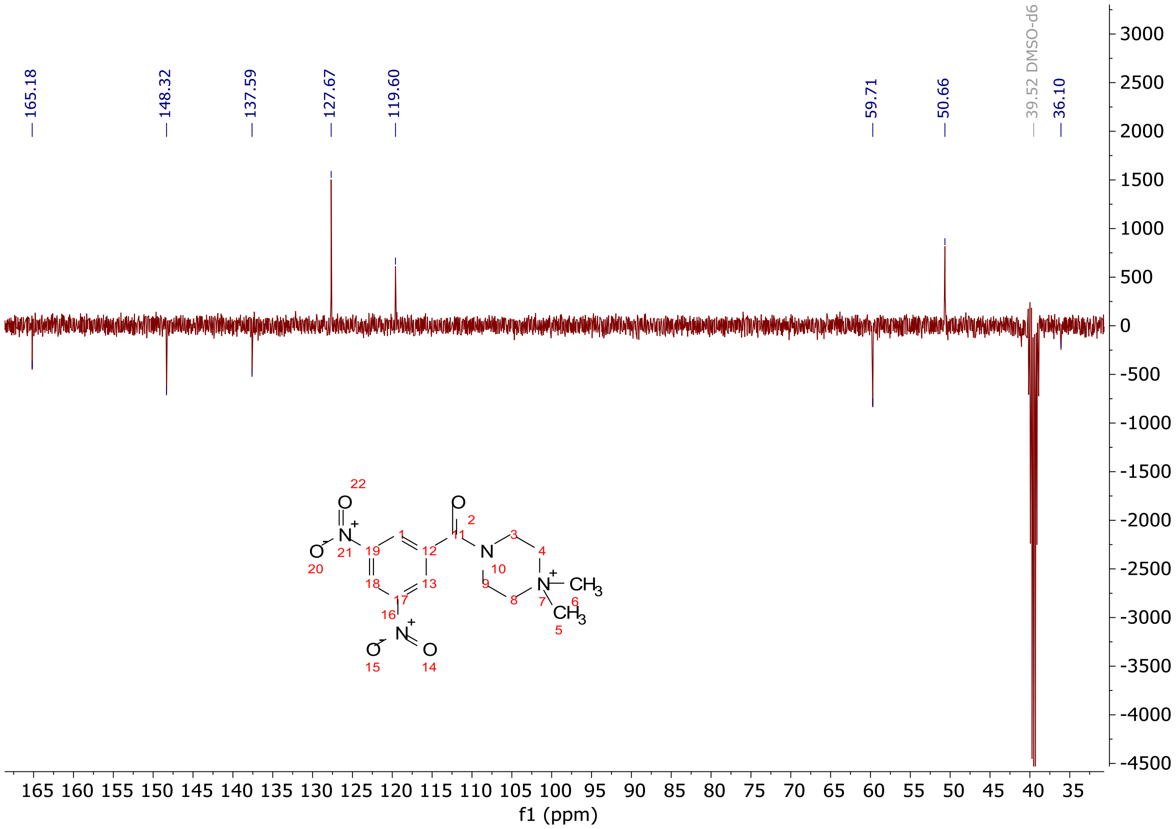

Figure S3.14: ^{13}C-APT NMR (101 MHz, DMSO) spectrum of **a8**

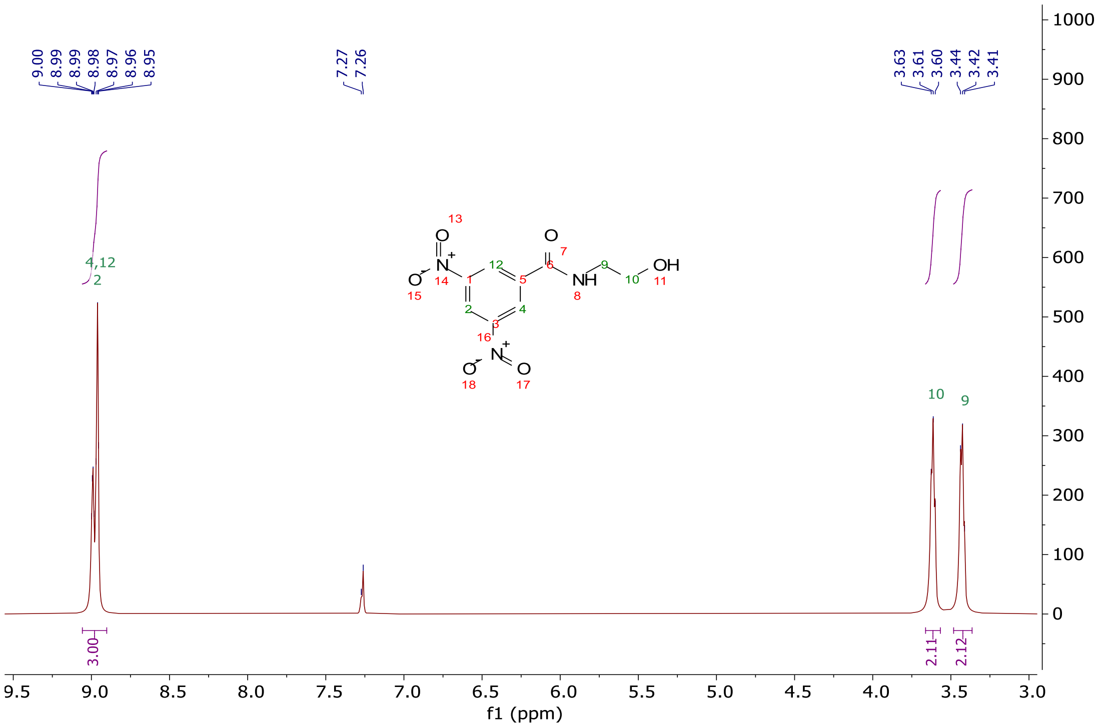

Figure S3.15: ^{1}H NMR (400 MHz, $CDCl_3$) spectrum of **b1**

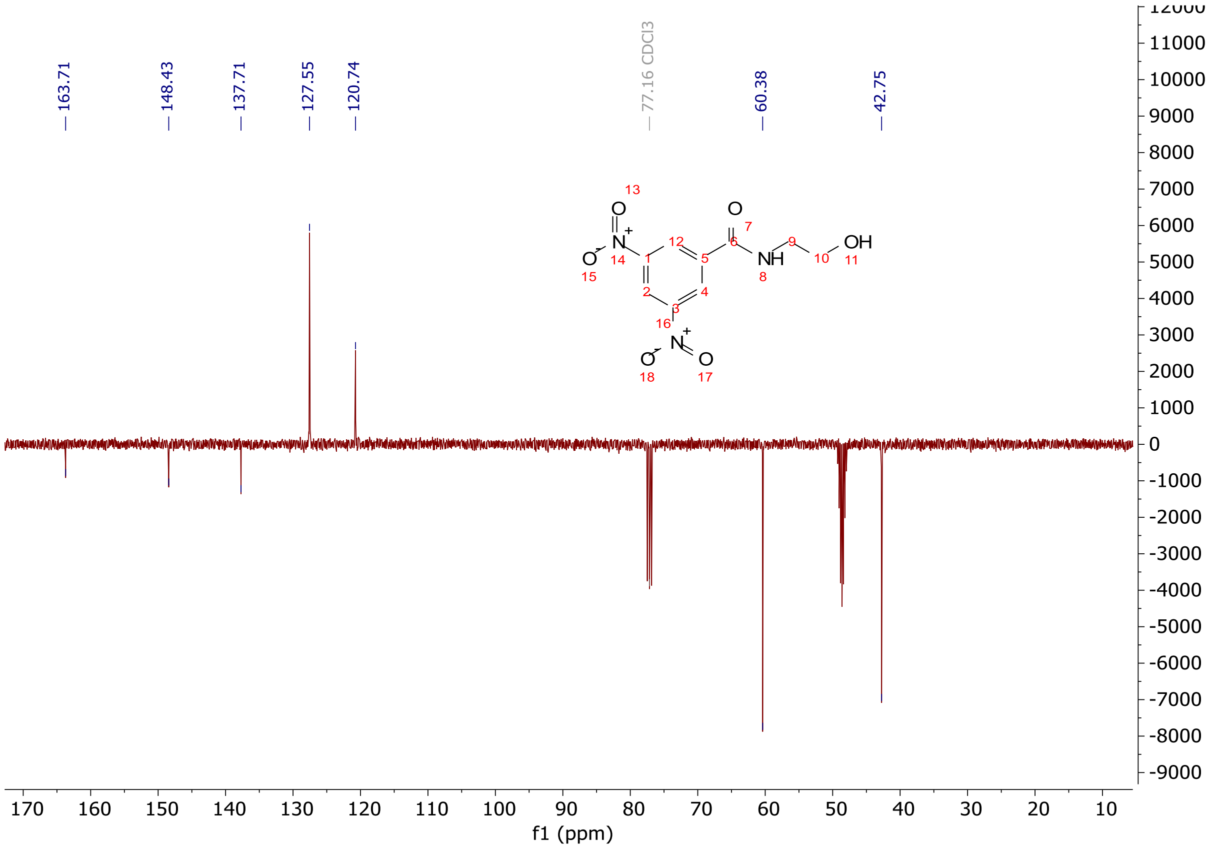

Figure S3.16: ^{13}C-APT NMR (101 MHz, $CDCl_3$) spectrum of **b1**

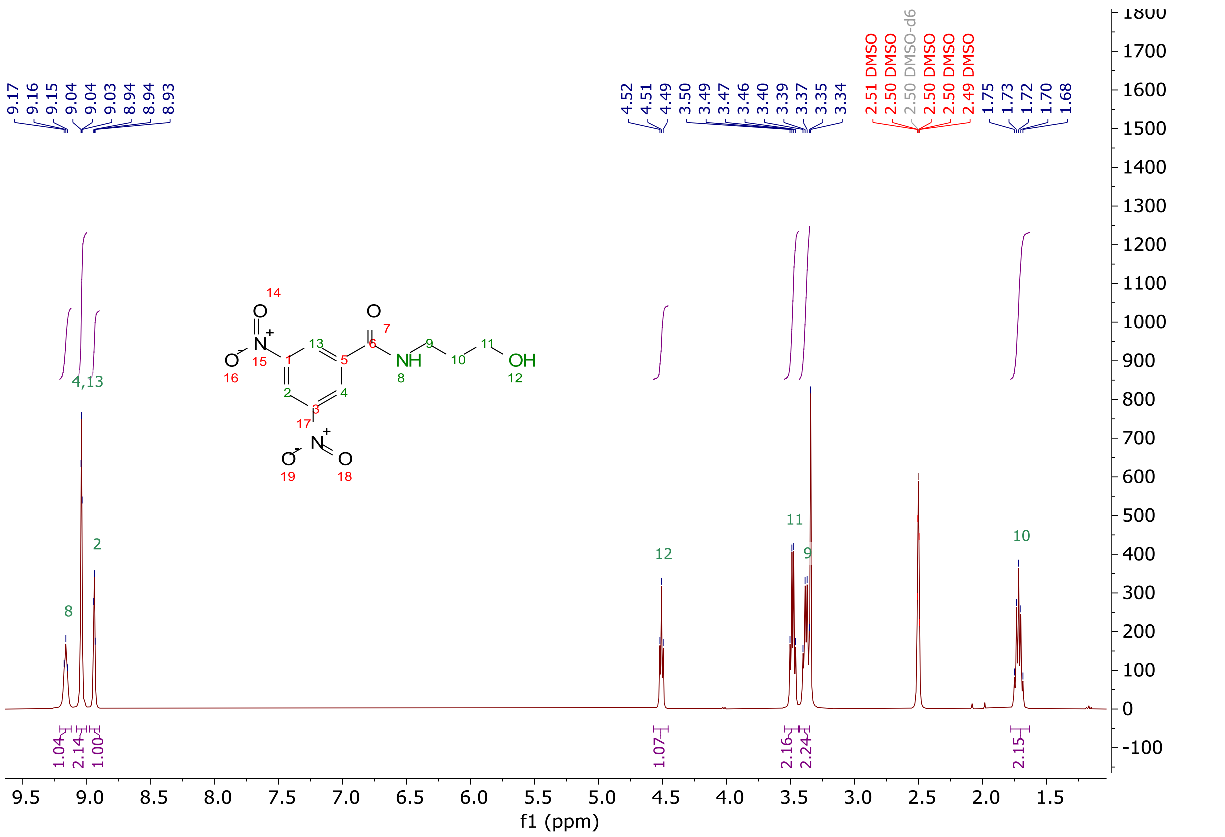

Figure S3.17: ^{1}H NMR (400 MHz, DMSO) spectrum of **b2**

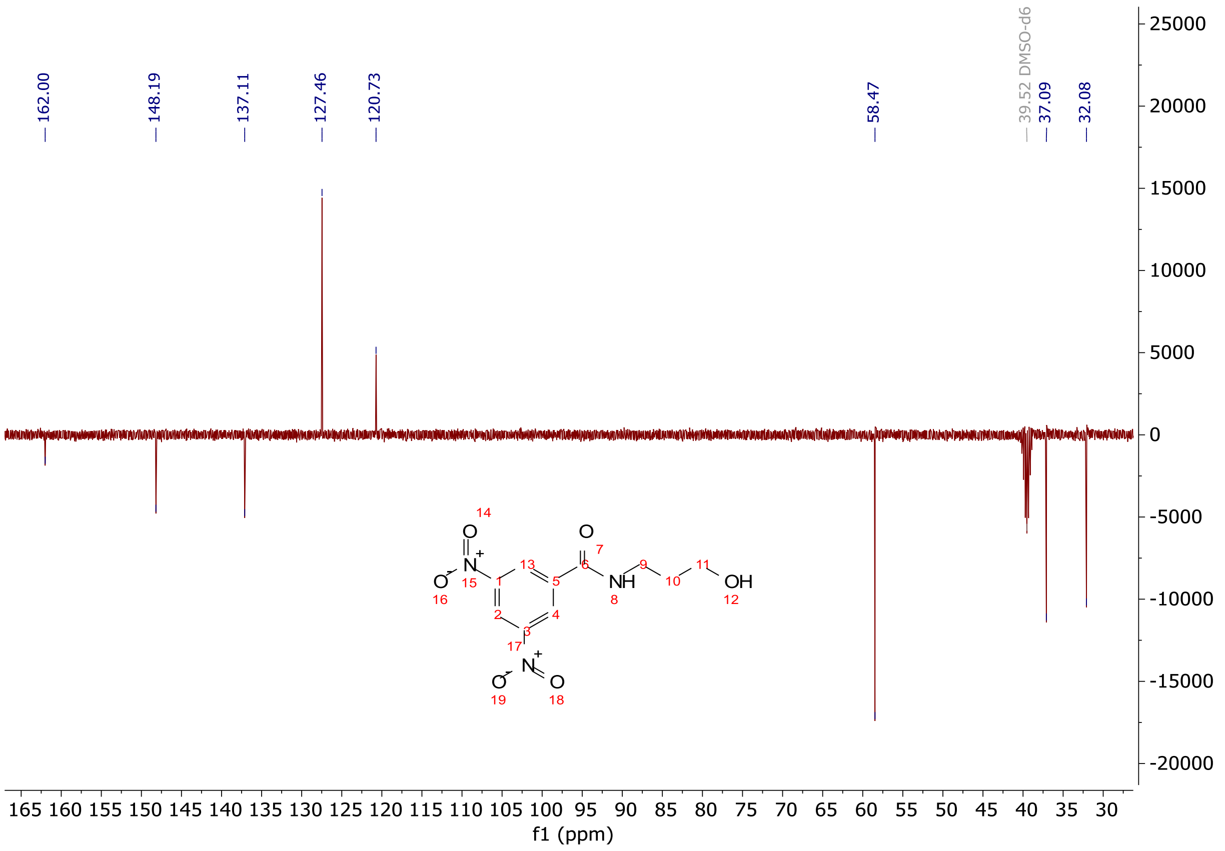

Figure S3.18: ^{13}C-APT NMR (101 MHz, DMSO) spectrum of **b2**

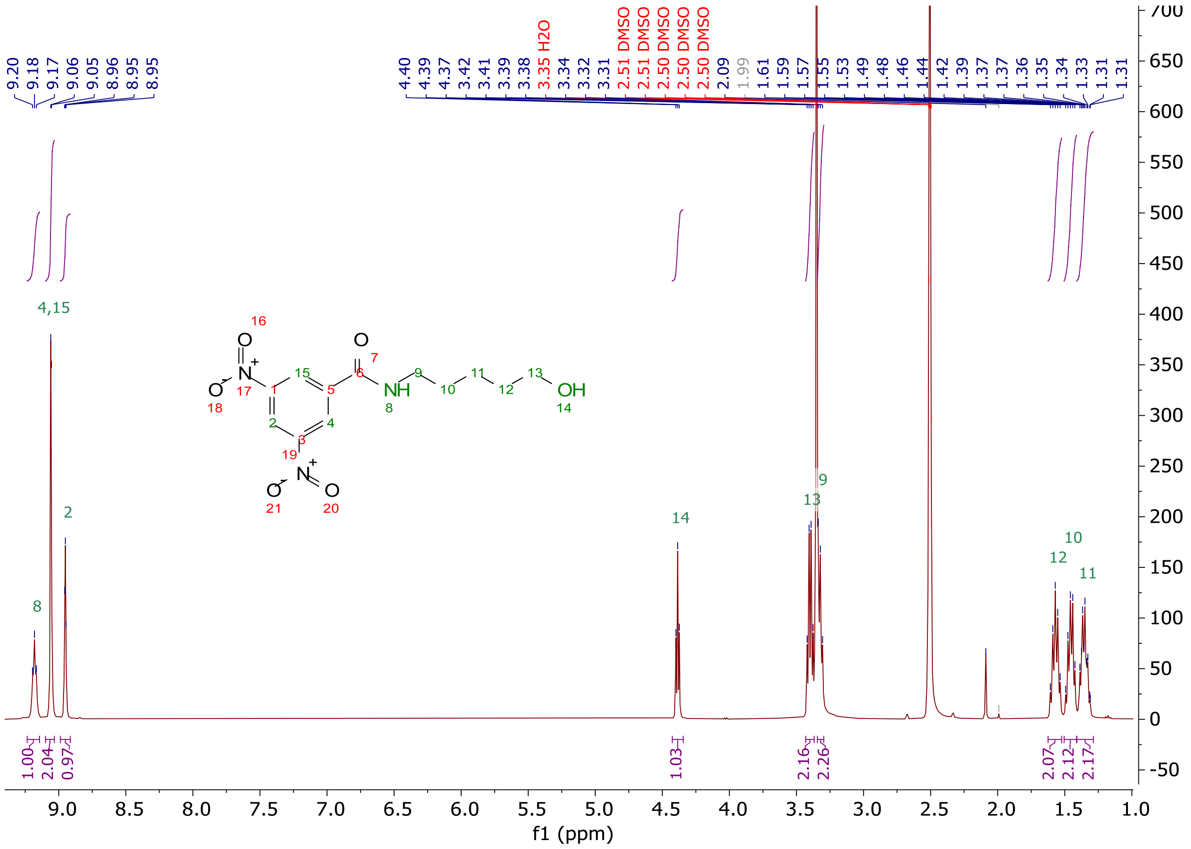

Figure S3.19: ^{1}H NMR (400 MHz, DMSO) spectrum of **b3**

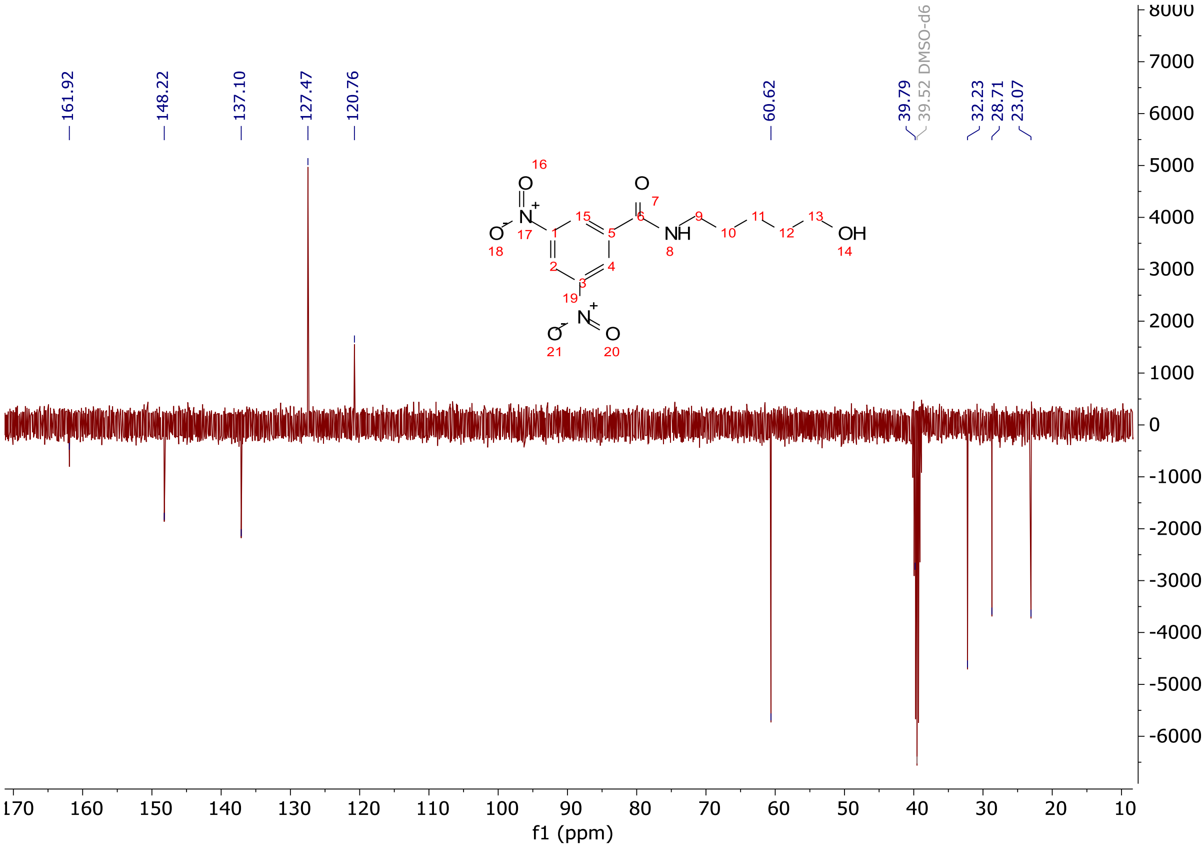

Figure S3.20: ^{13}C-APT NMR (101 MHz, DMSO) spectrum of **b3**

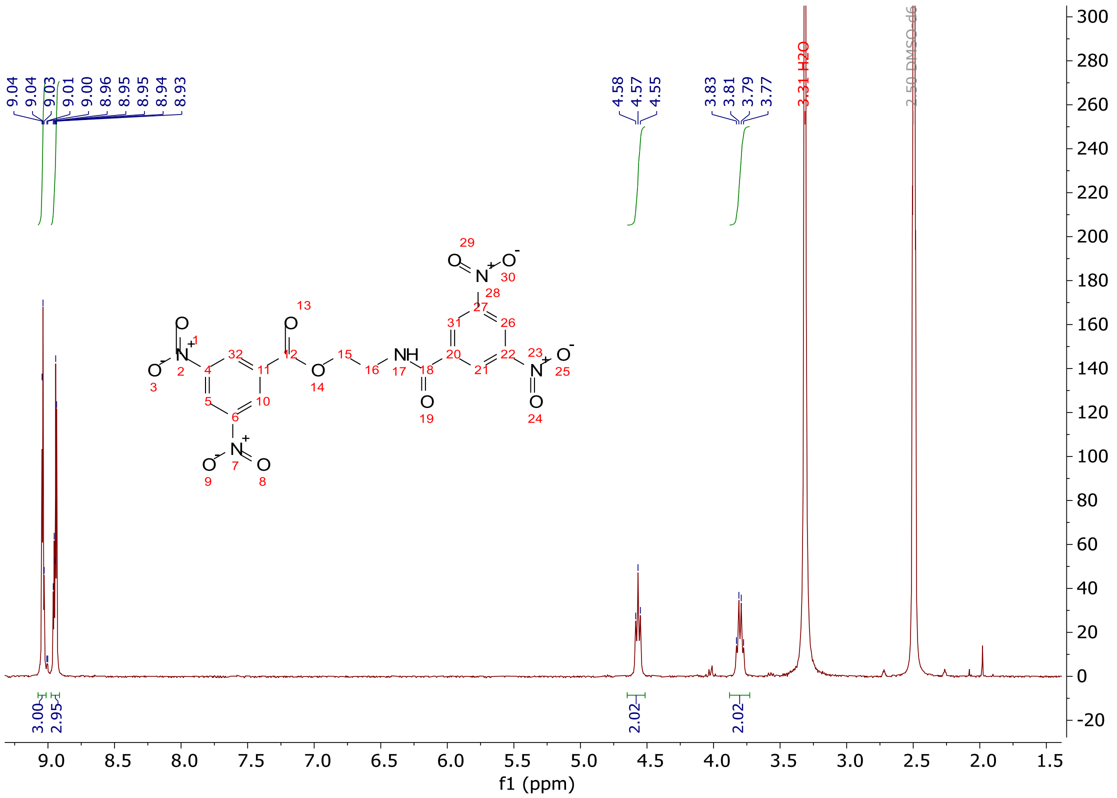

Figure S3.21: ^{1}H NMR (400 MHz, $CDCl_3$) spectrum of **c1**

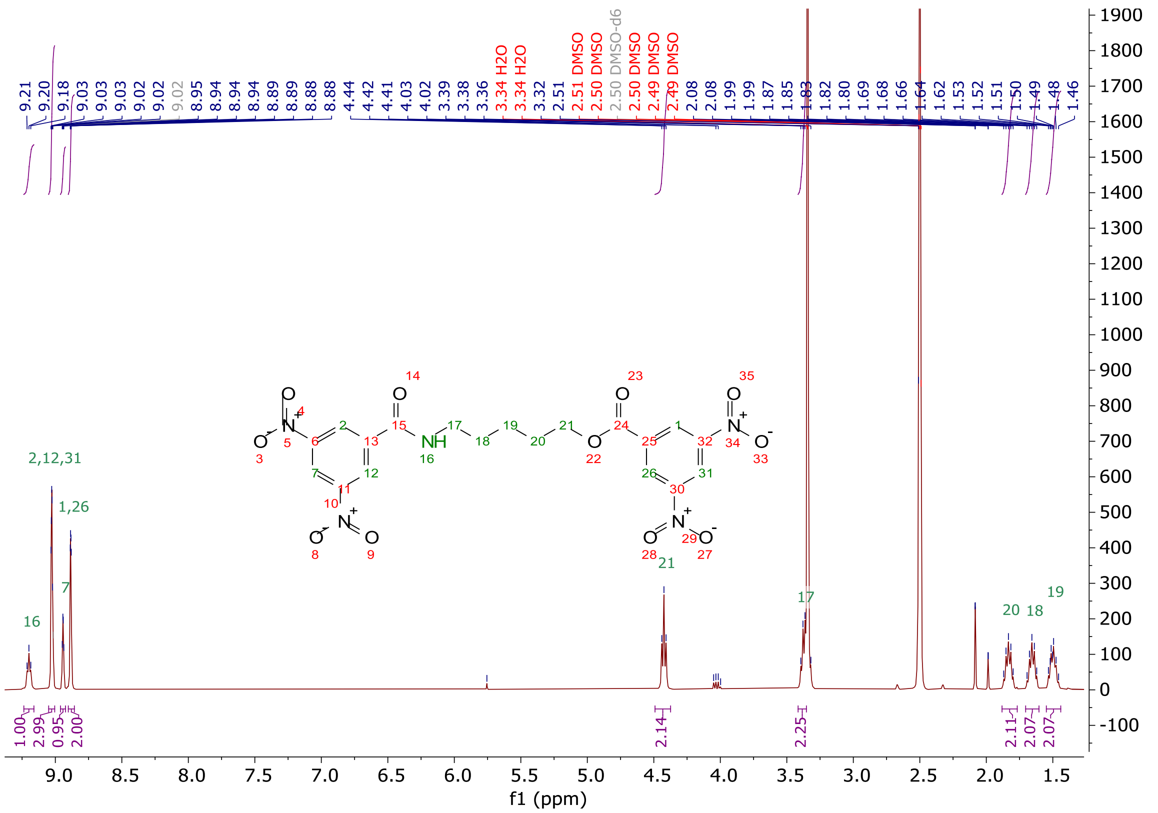

Figure S3.22: ^{1}H NMR (400 MHz, DMSO) spectrum of **c2**

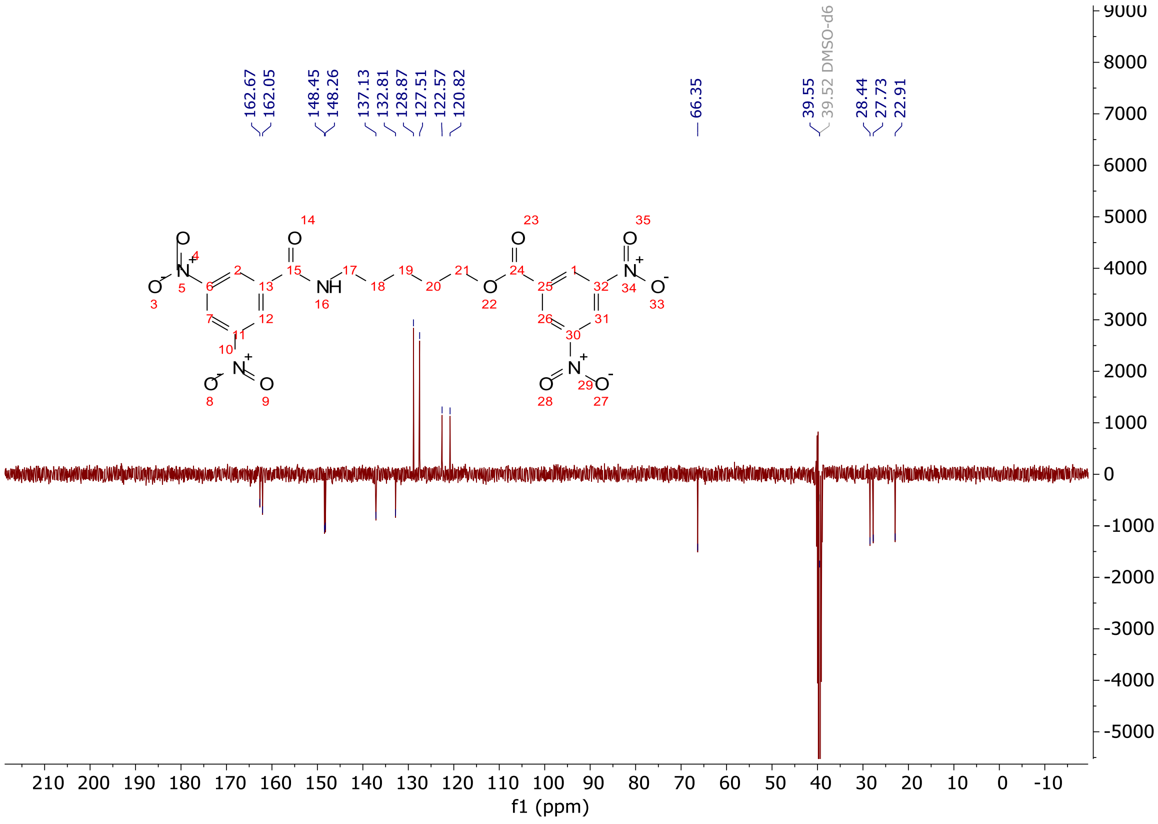

Figure S3.23: ^{13}C-APT NMR (101 MHz, DMSO) spectrum of **c2**

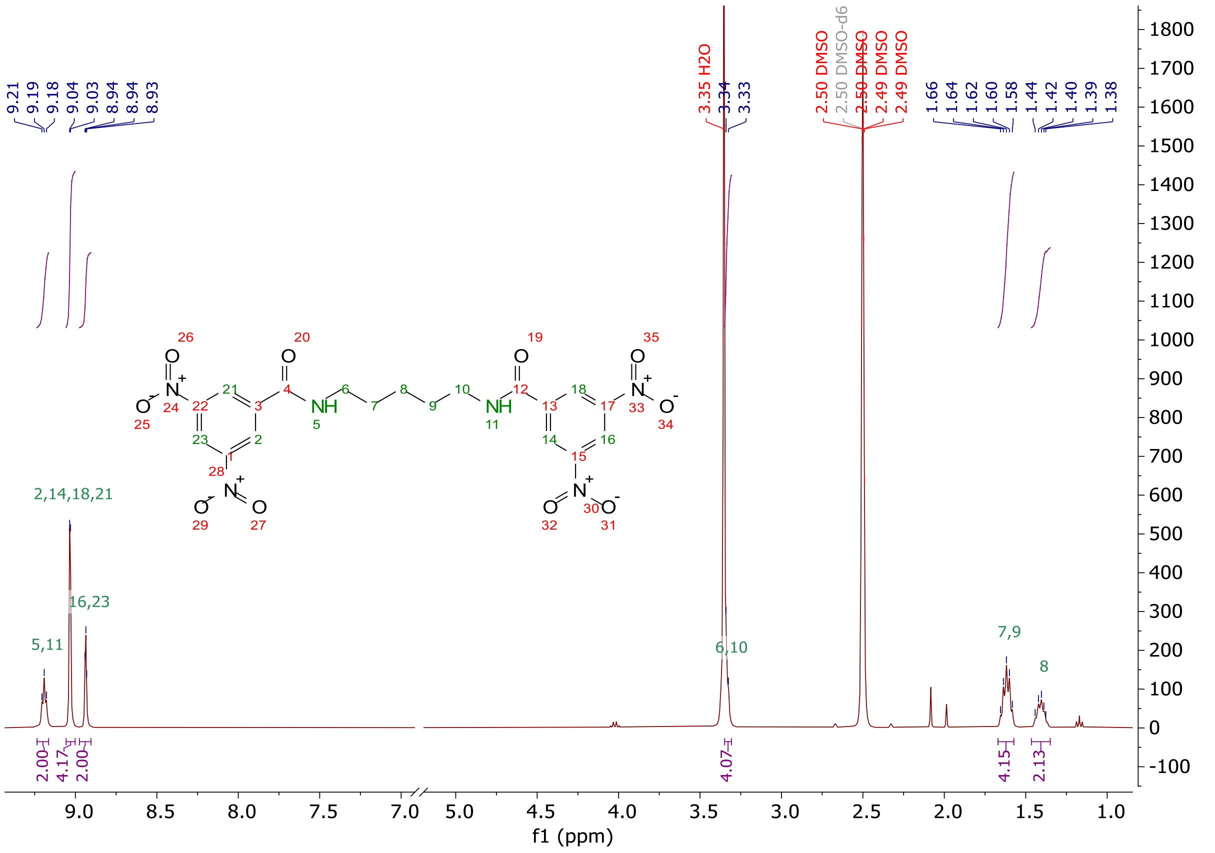

Figure S3.24: ^{1}H NMR (400 MHz, DMSO) spectrum of **c3**

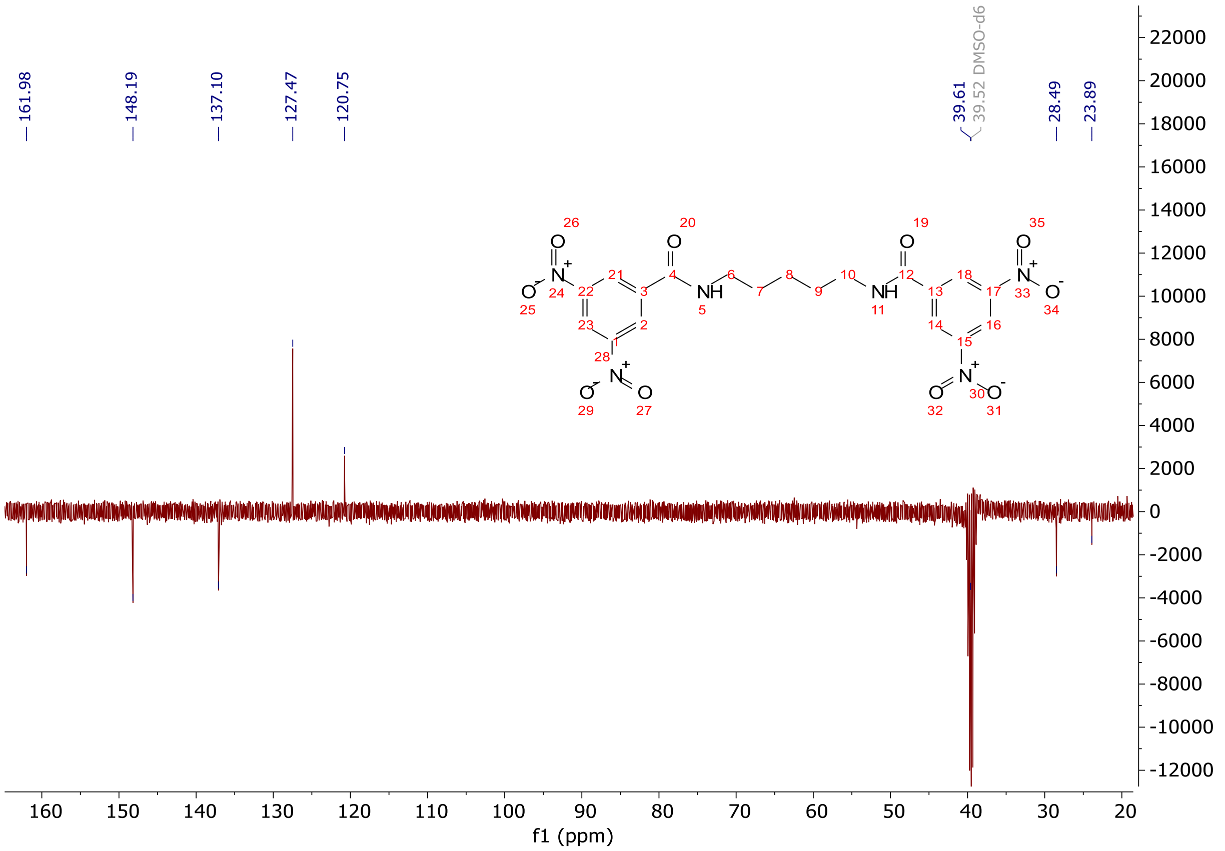

Figure S3.25: ^{13}C-APT NMR (101 MHz, DMSO) spectrum of **c3**

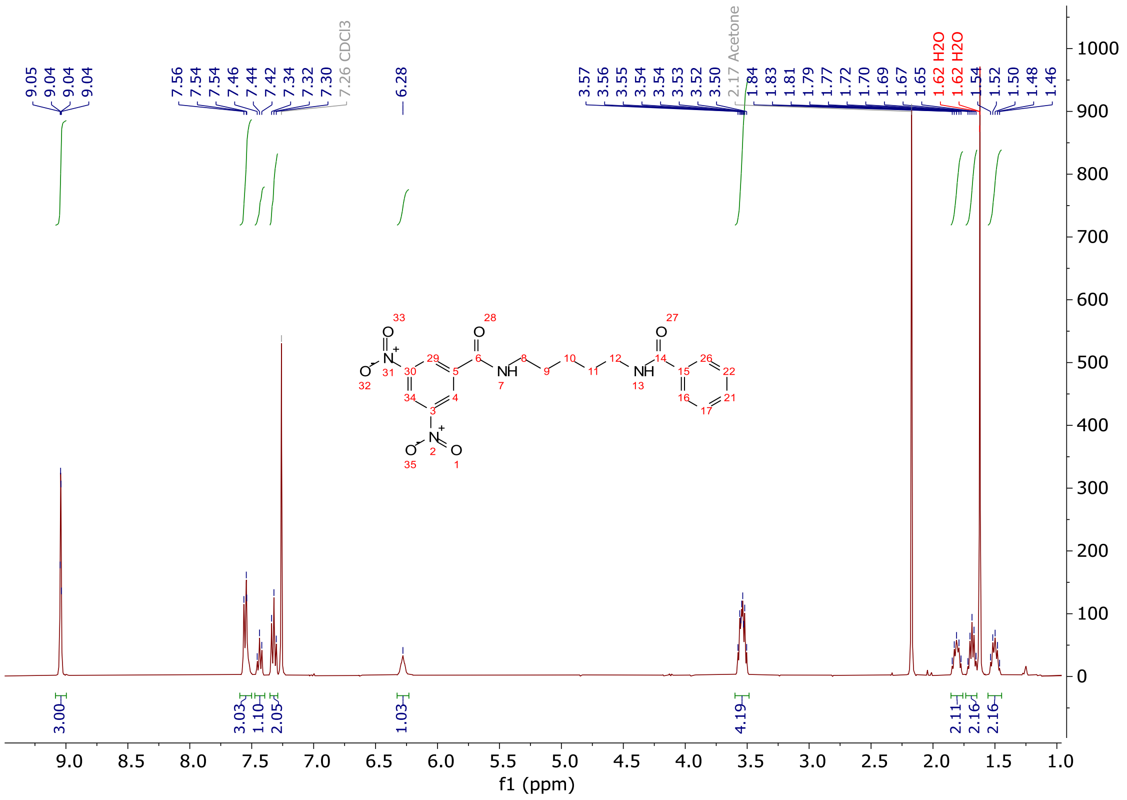

Figure S3.26: ^{1}H NMR (400 MHz, $CDCl_3$) spectrum of **c4**

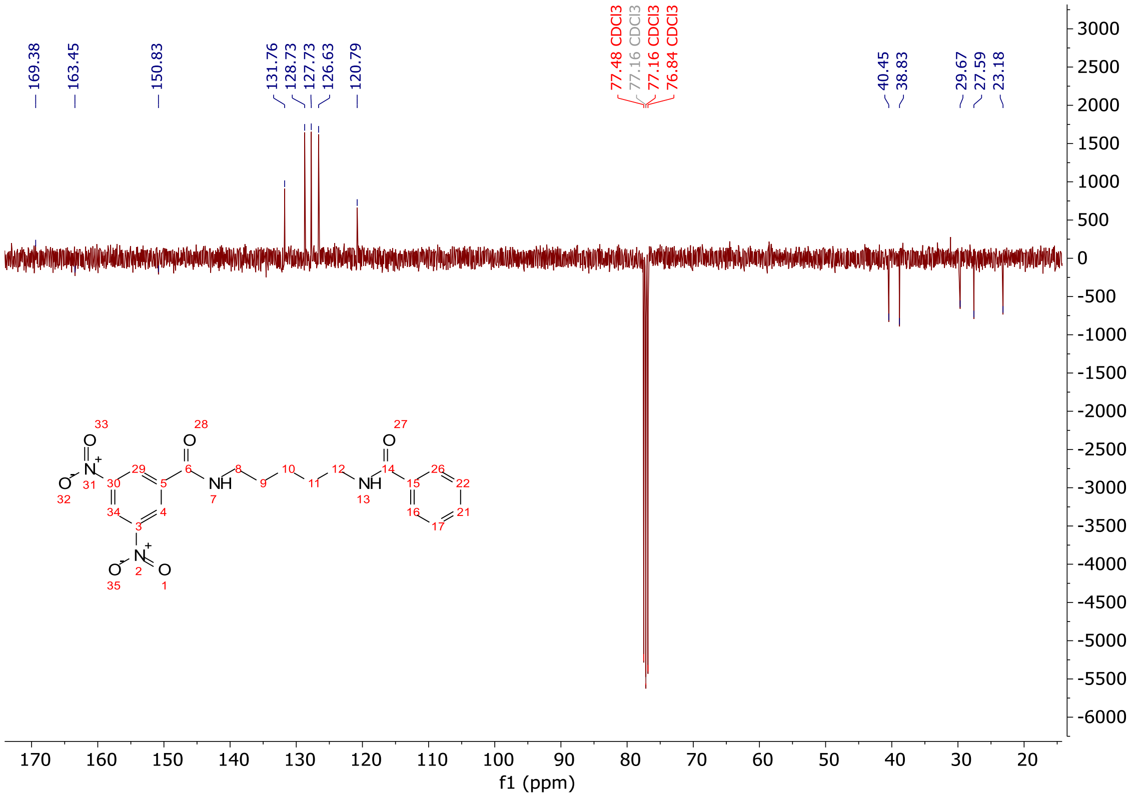

Figure S3.27: ^{13}C-APT NMR (101 MHz, $CDCl_3$) spectrum of **c4**

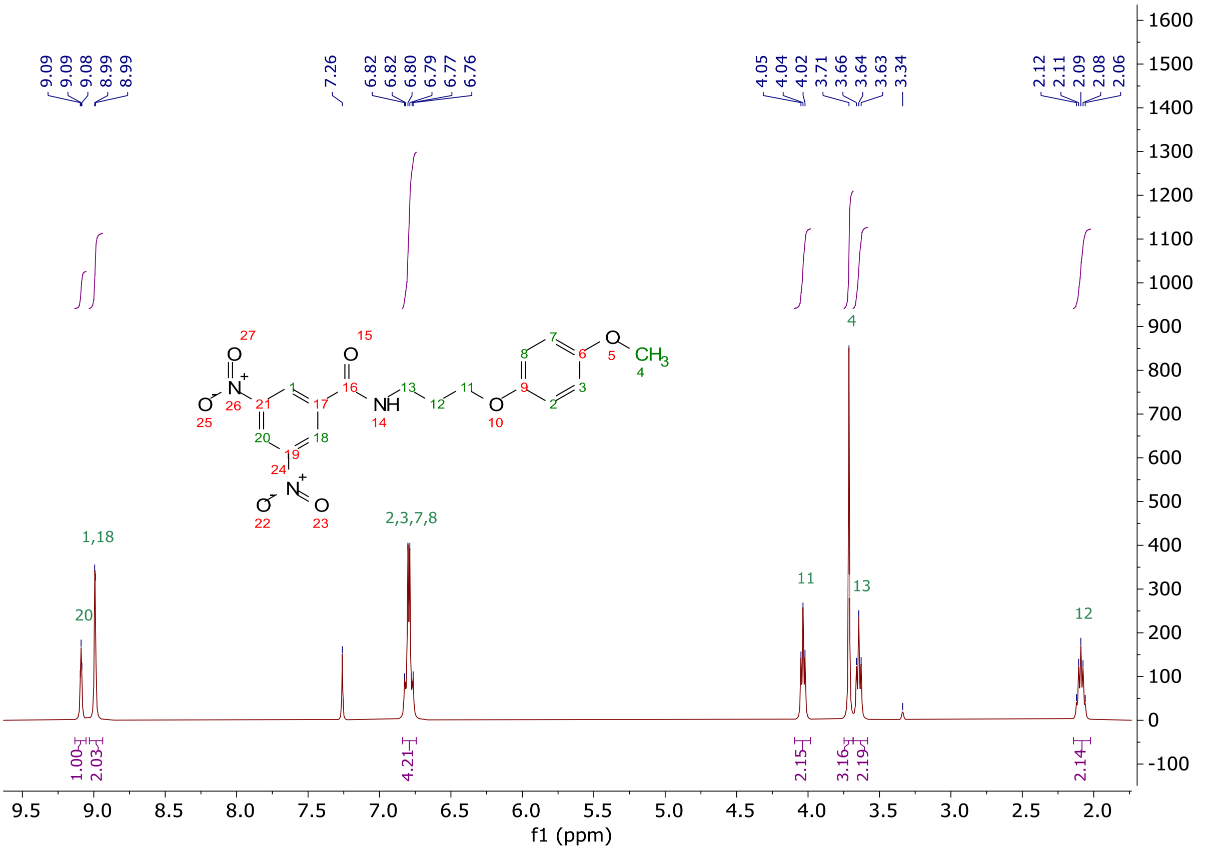

Figure S3.28: ^{1}H NMR (400 MHz, $CDCl_3$) spectrum of **d1**

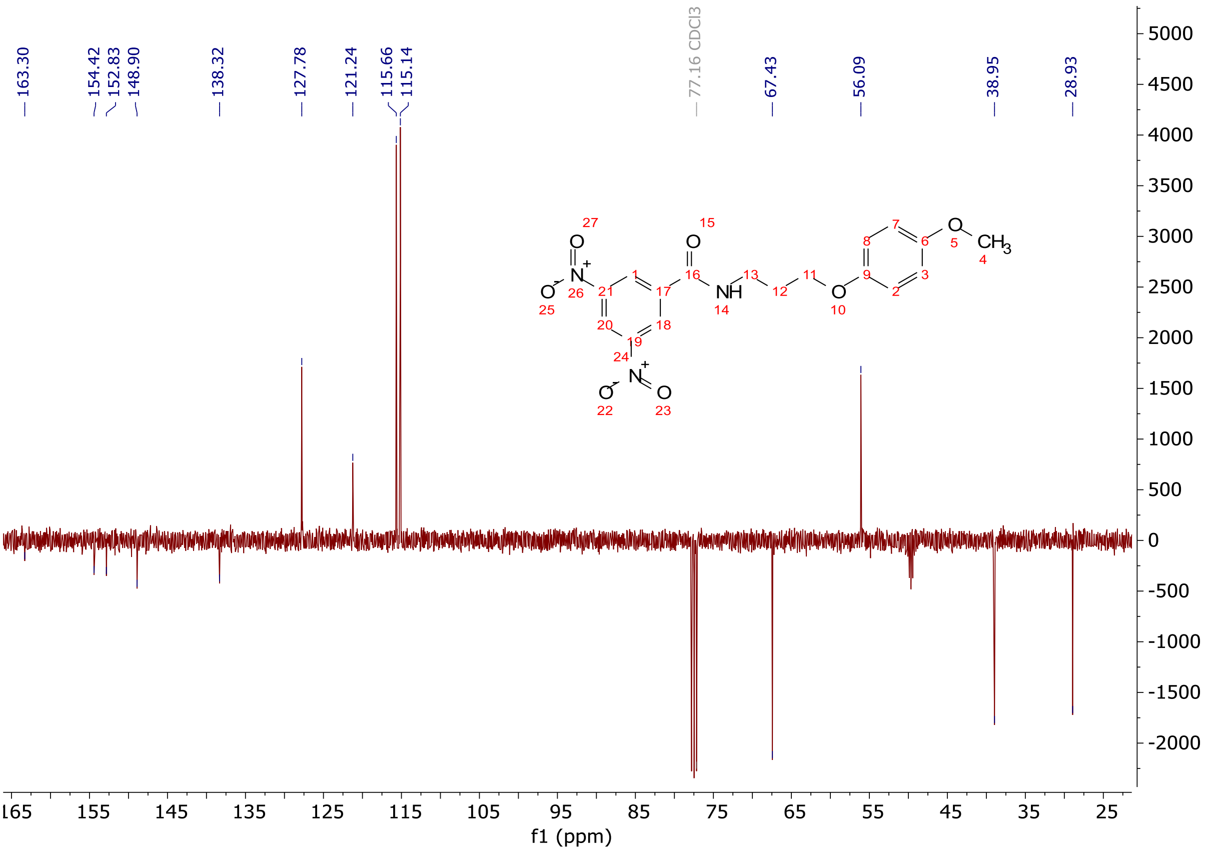

Figure S3.29: ^{13}C-APT NMR (101 MHz, $CDCl_3$) spectrum of **d1**

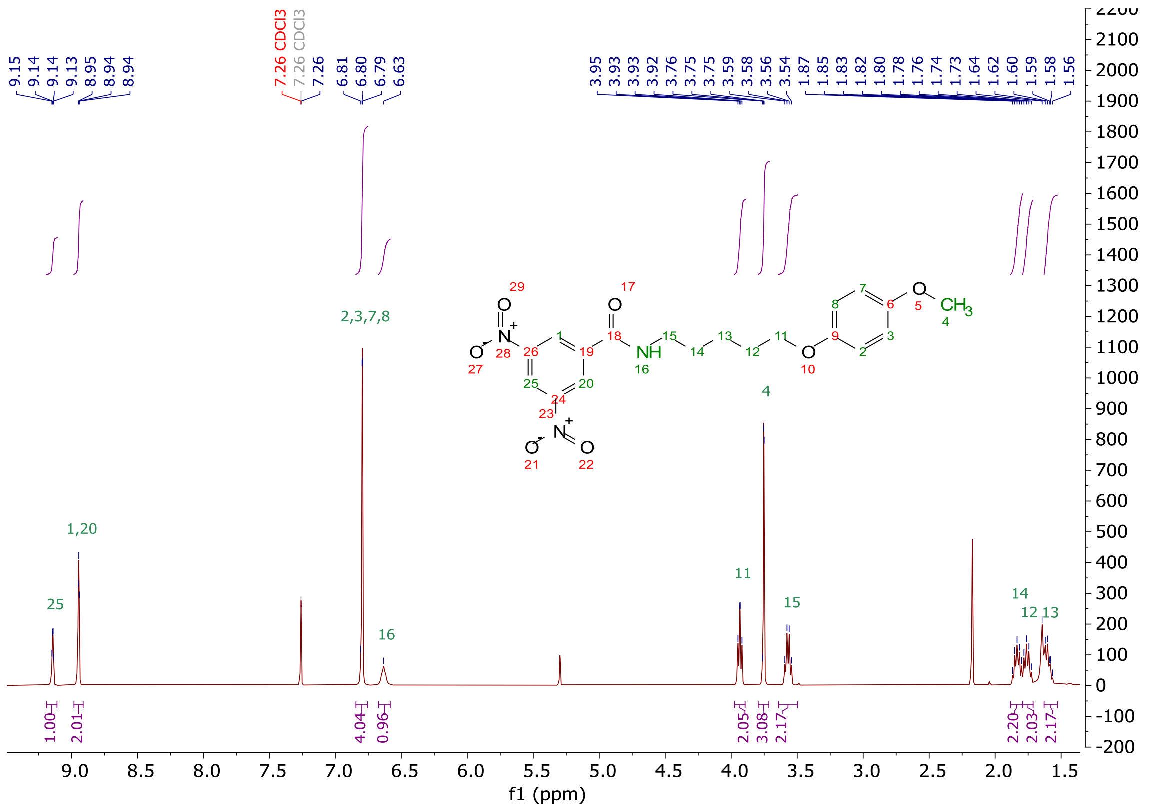

Figure S3.30: ^{1}H NMR (400 MHz, $CDCl_3$) spectrum of **d2**

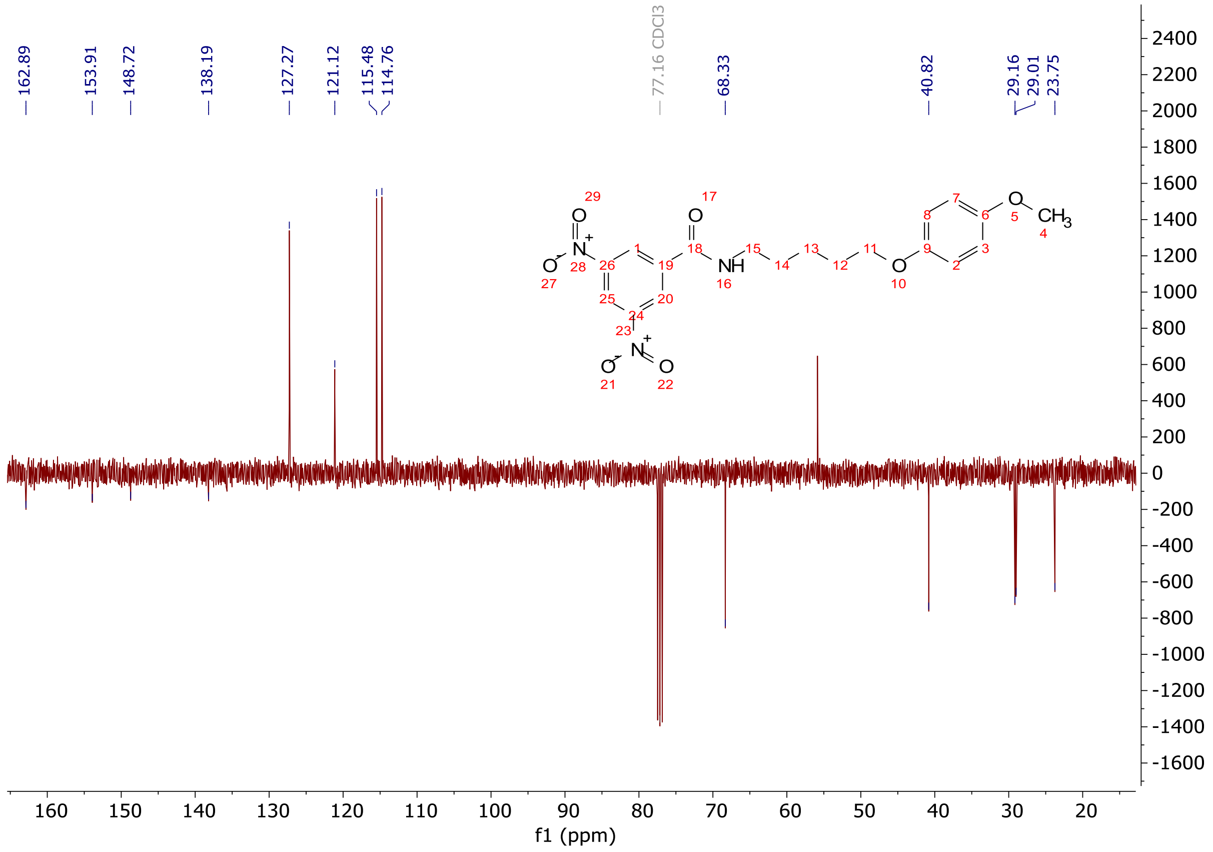

Figure S3.31: ^{13}C-APT NMR (101 MHz, $CDCl_3$) spectrum of **d2**

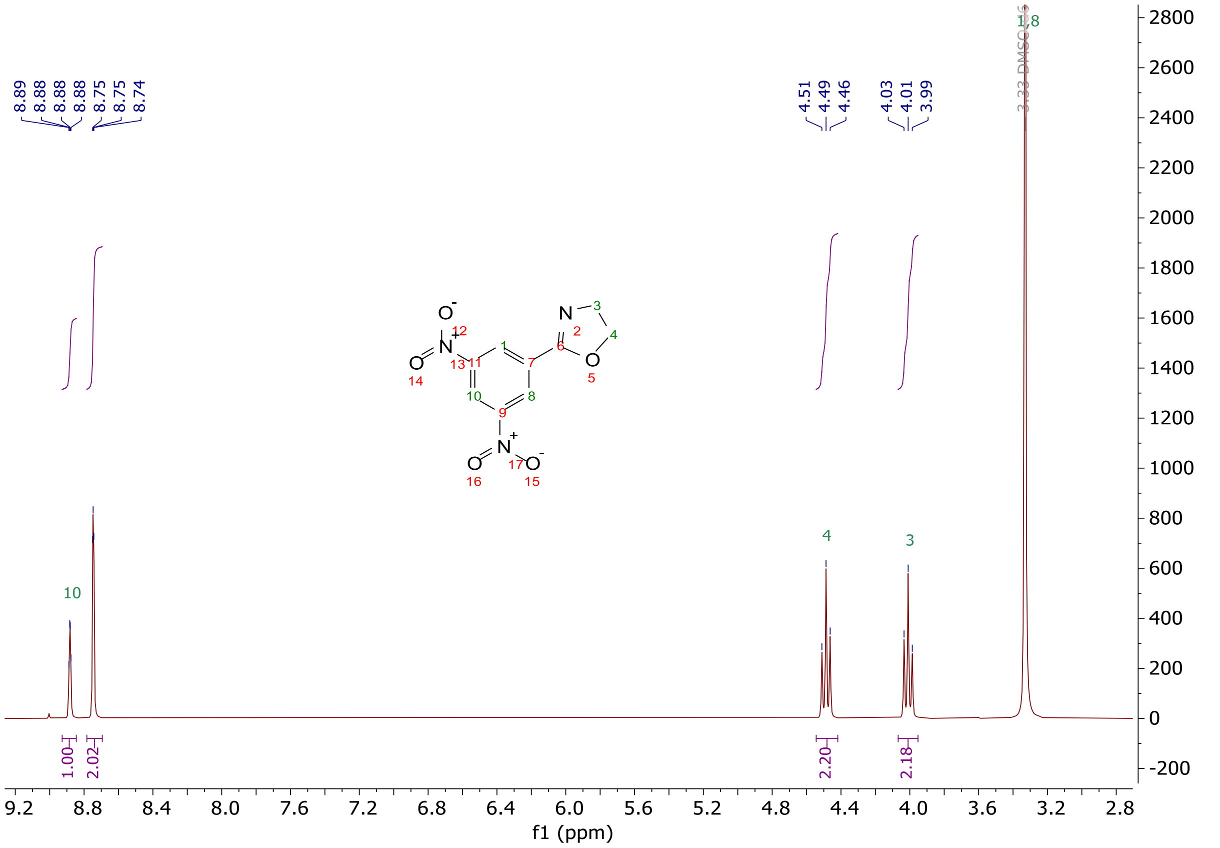

Figure S3.32: ^{1}H NMR (400 MHz, DMSO) spectrum of **e1**

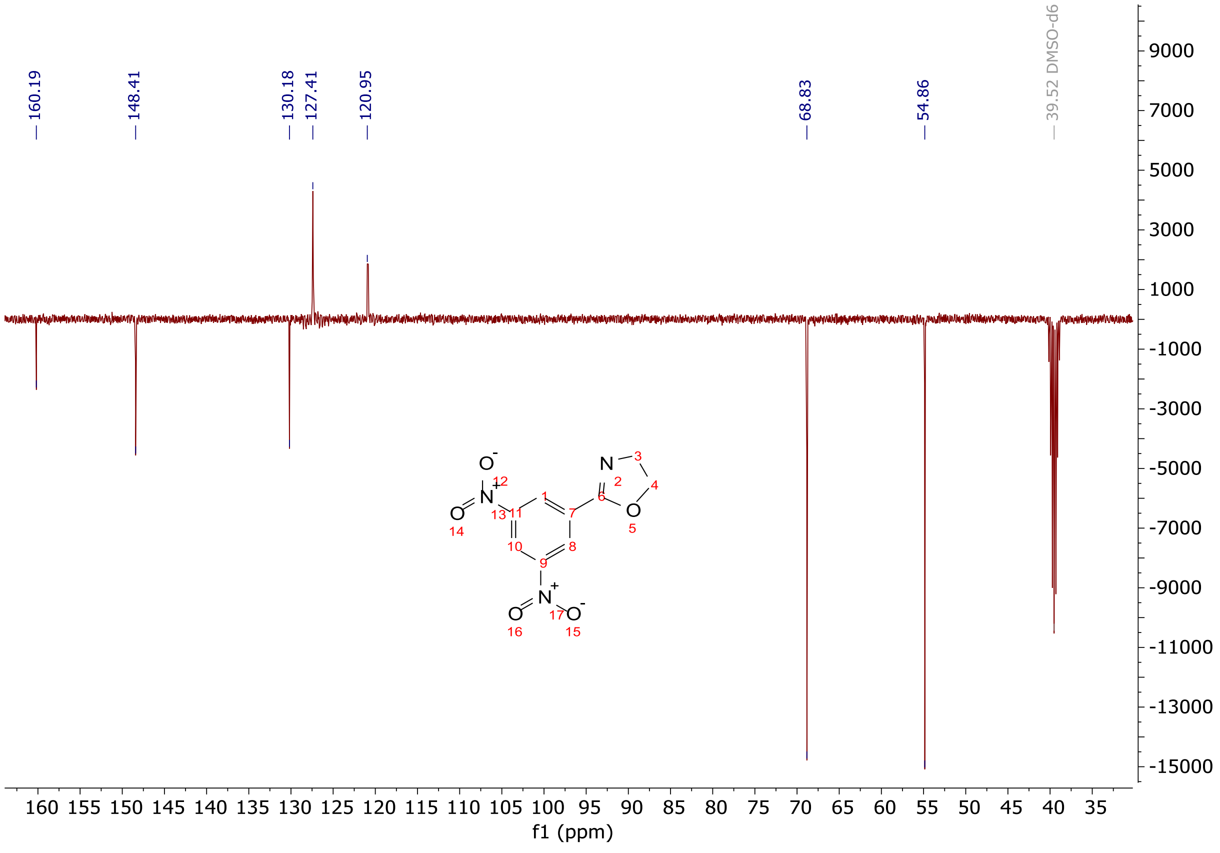

Figure S3.33: ^{13}C-APT NMR (101 MHz, DMSO) spectrum of **e1**

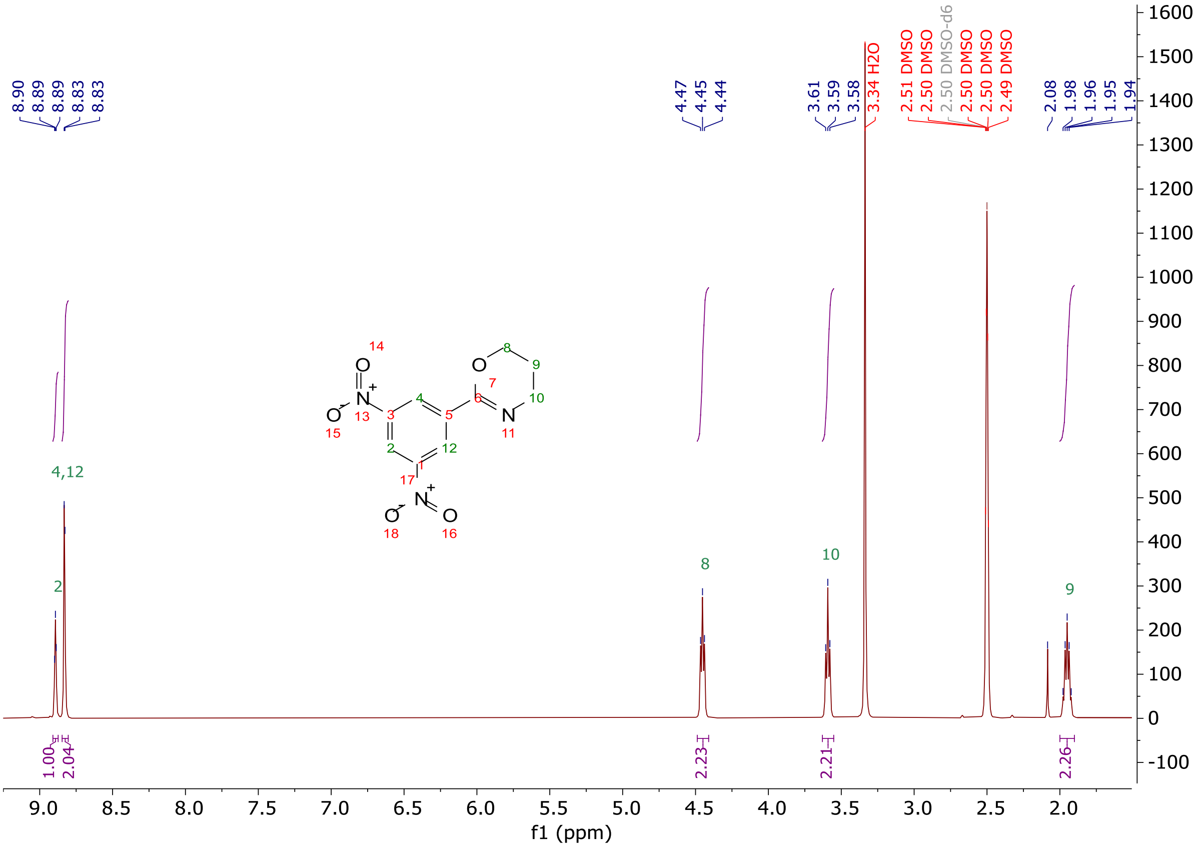

Figure S3.34: ^{1}H NMR (400 MHz, DMSO) spectrum of **e2**

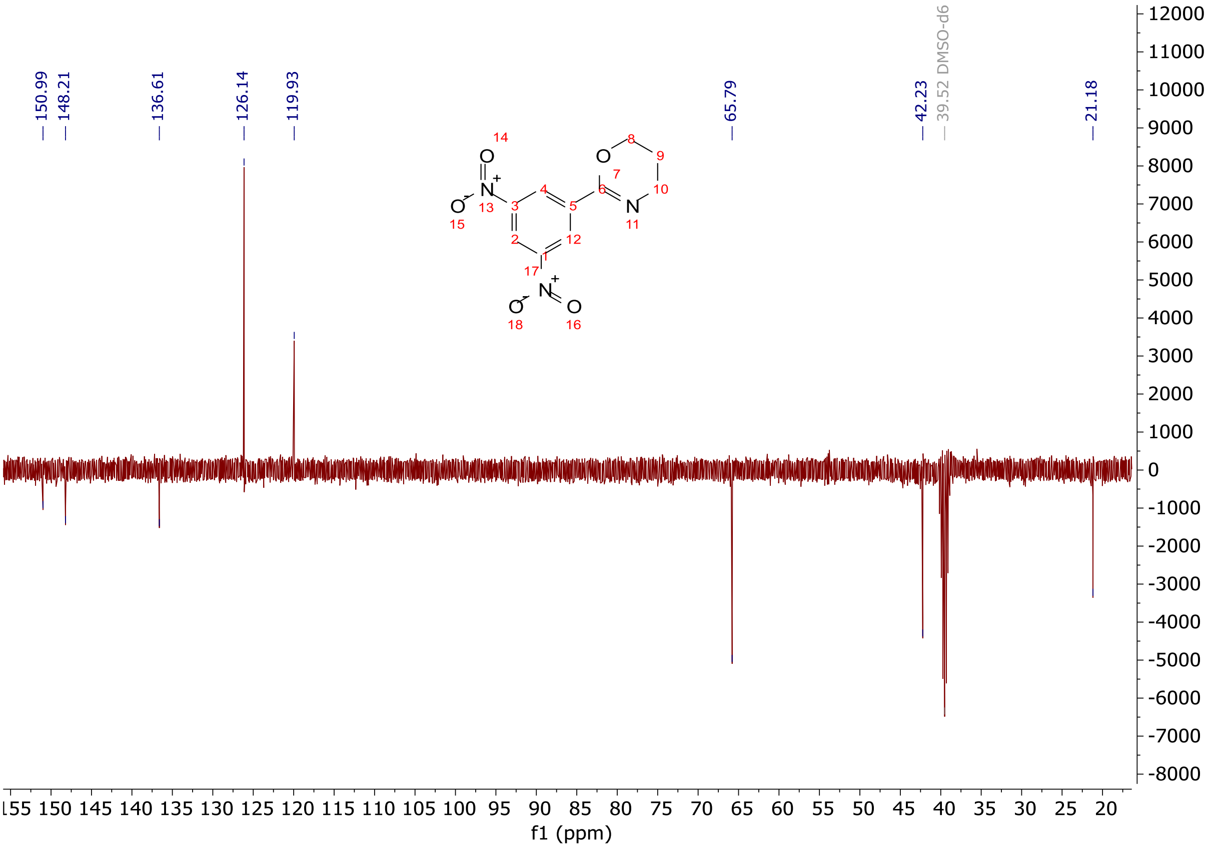

Figure S3.35: ^{13}C-APT NMR (101 MHz, DMSO) spectrum of **e2**

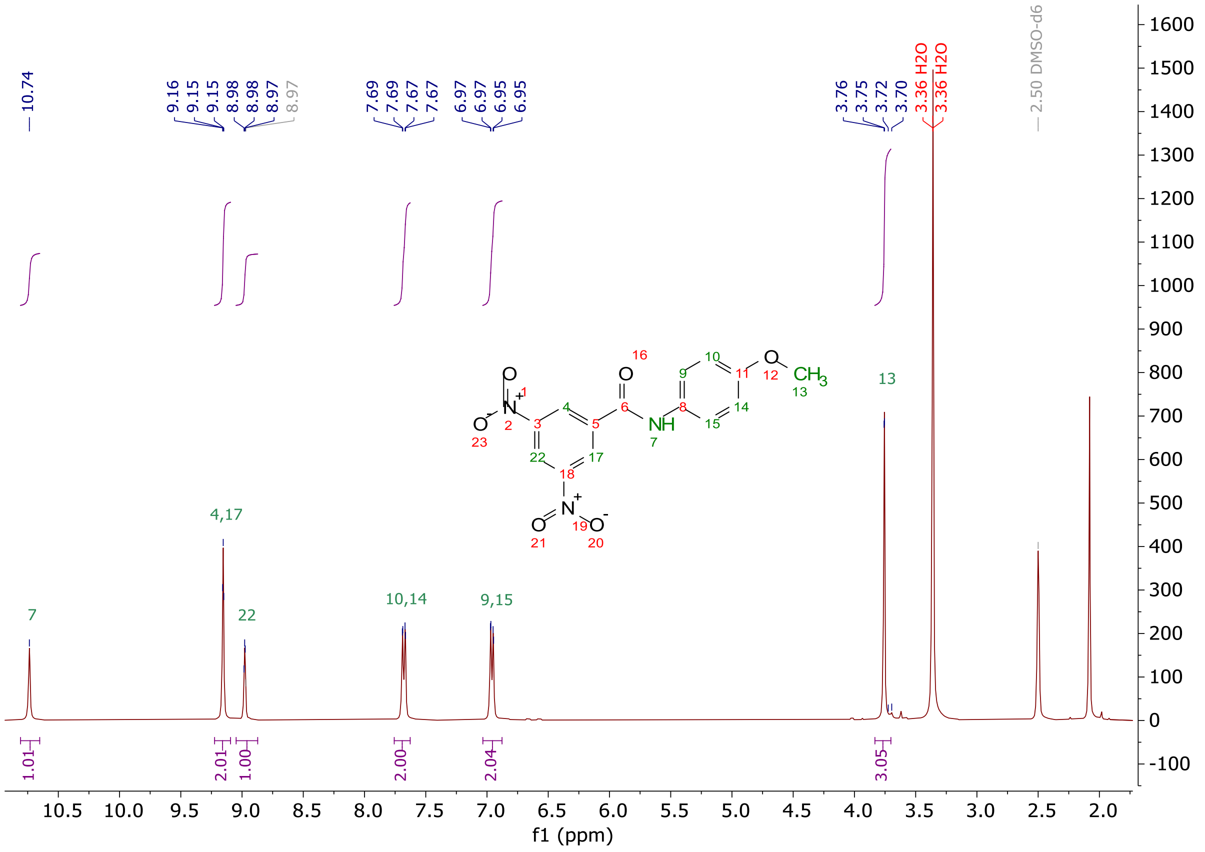

Figure S3.36: ^{1}H NMR (400 MHz, DMSO) spectrum of **f1**

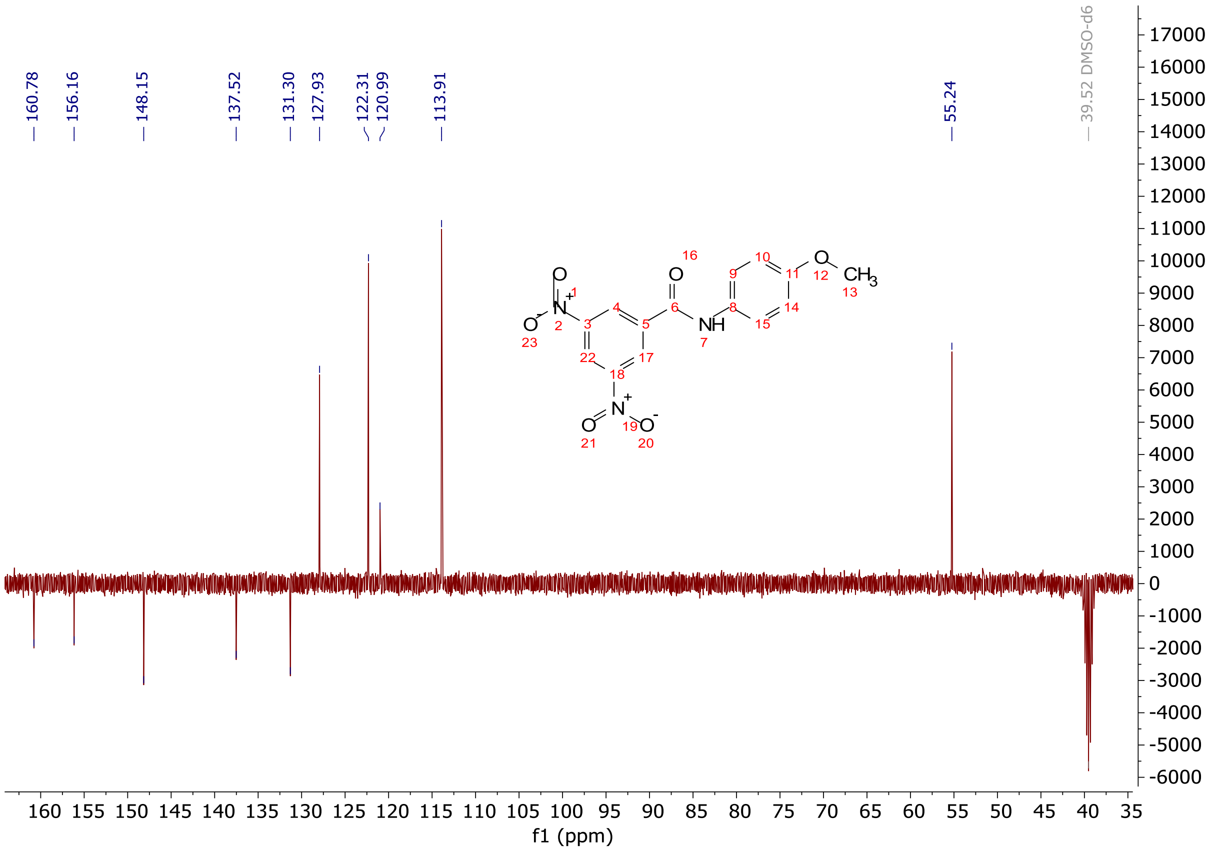

Figure S3.37: ^{13}C-APT NMR (101 MHz, DMSO) spectrum of **f1**

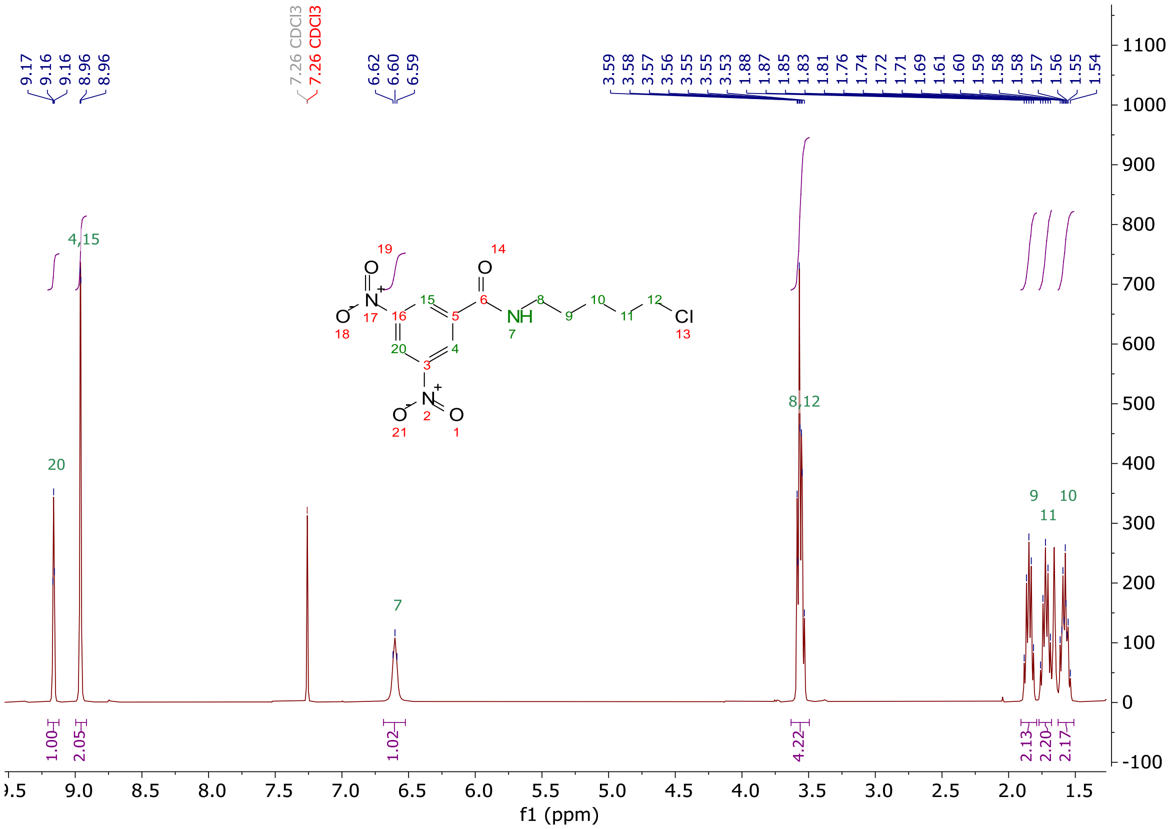

Figure S3.38: ^{1}H NMR (400 MHz, $CDCl_3$) spectrum of **3**

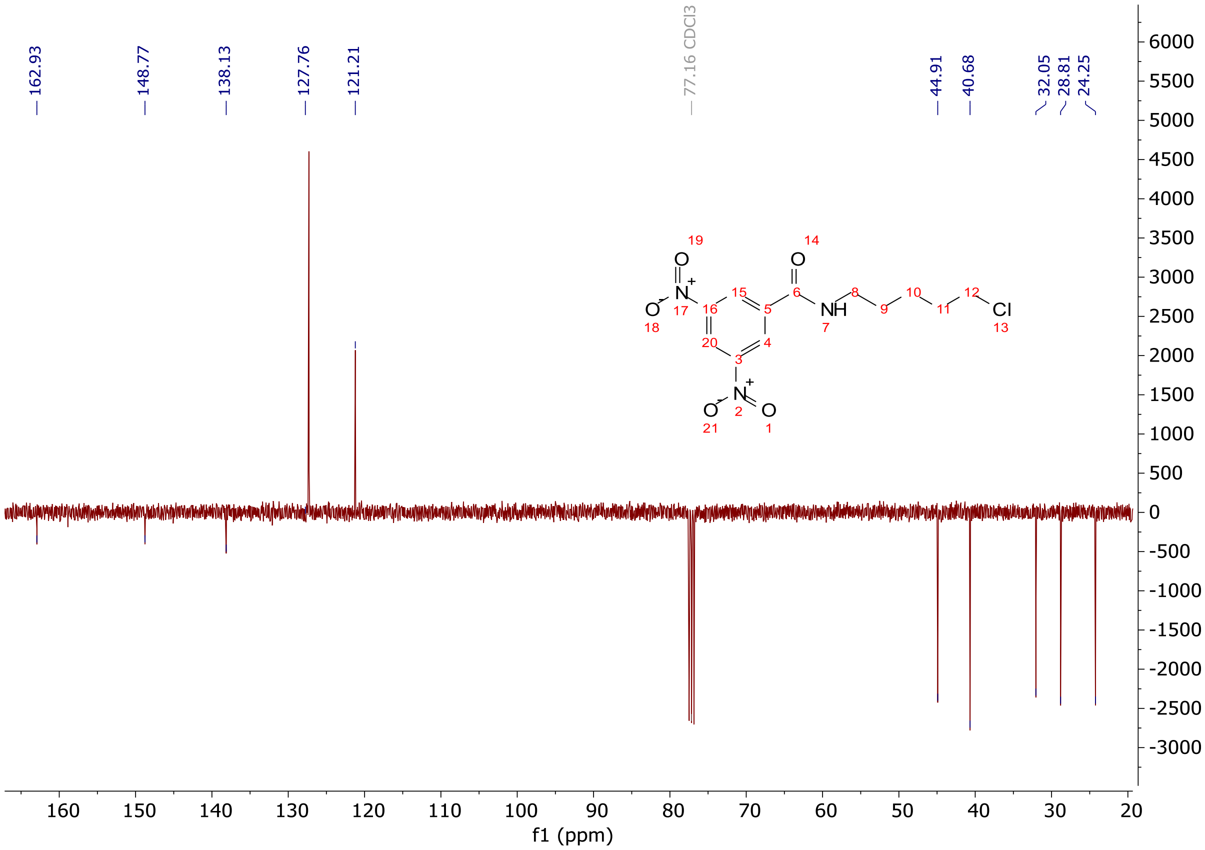

Figure S3.39: ^{13}C-APT NMR (101 MHz, $CDCl_3$) spectrum of **3**

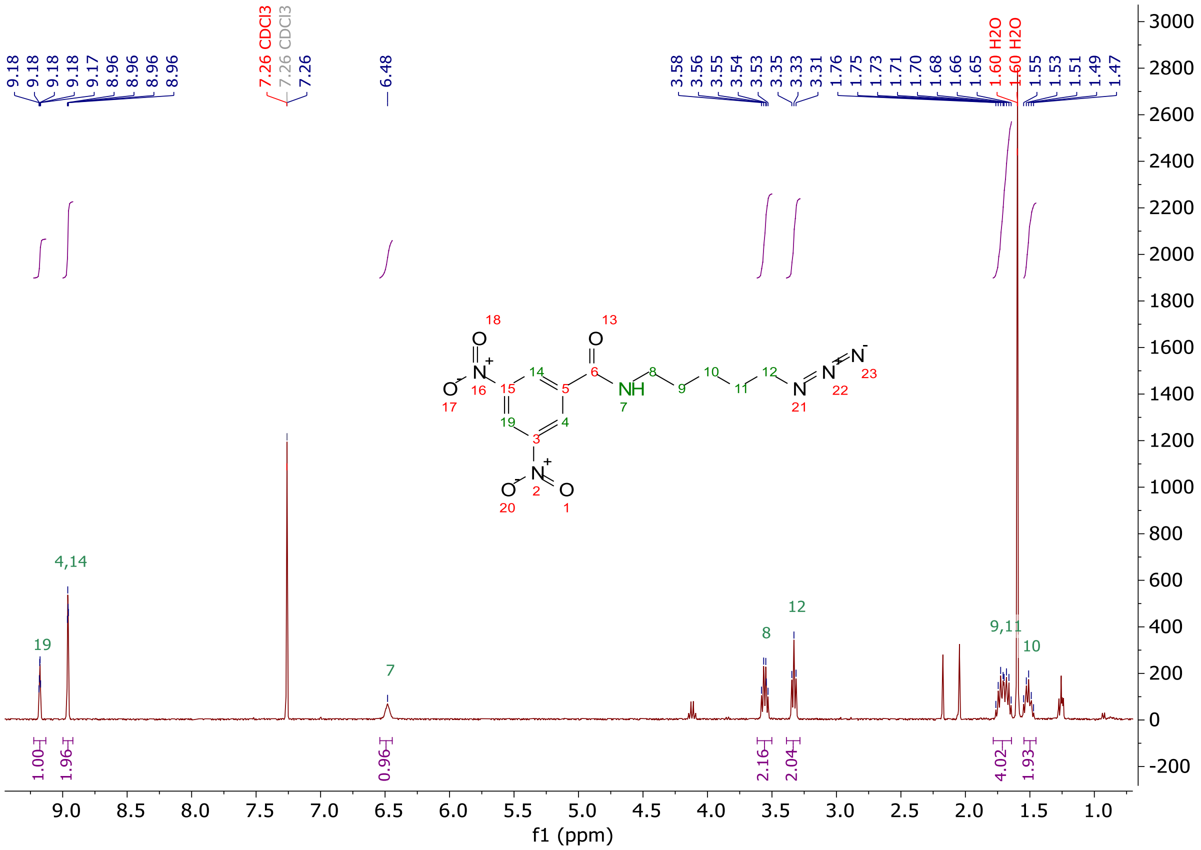

Figure S3.40: ^{1}H NMR (400 MHz, $CDCl_3$) spectrum of **4**

S4. Mass spectra of representative compounds:

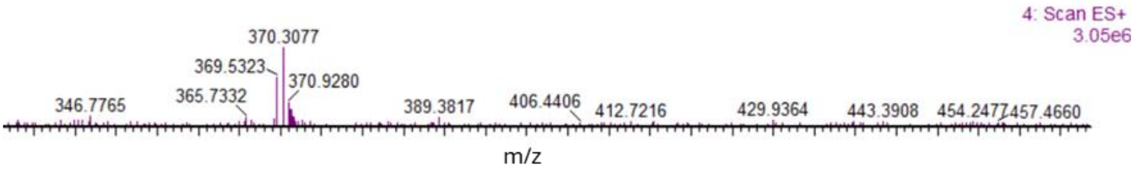

Figure S4.1: MS spectra of **a6**

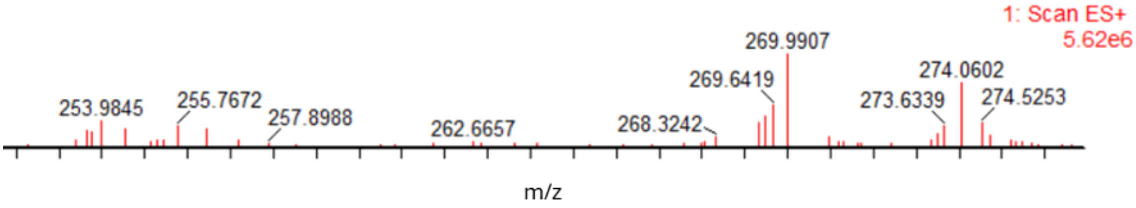

Figure S4.2: MS spectra of **b2**

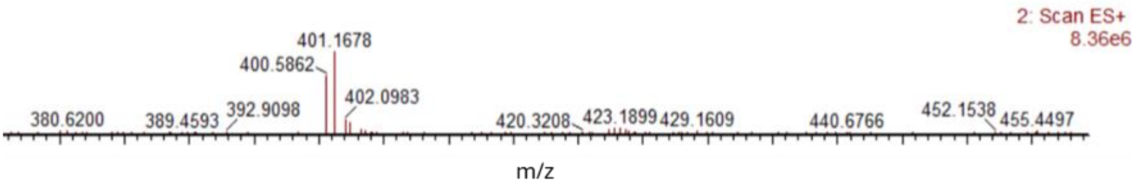

Figure S4.3: MS spectra of **c4**

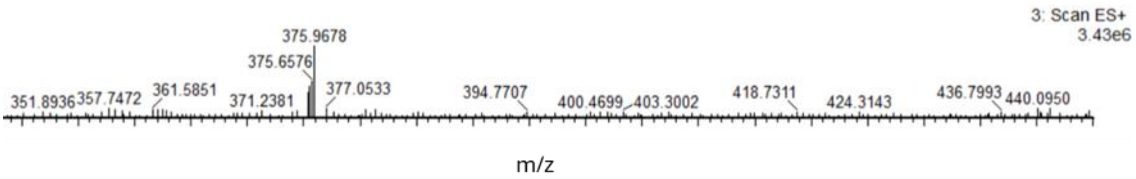

Figure S4.4: MS spectra of **d1**

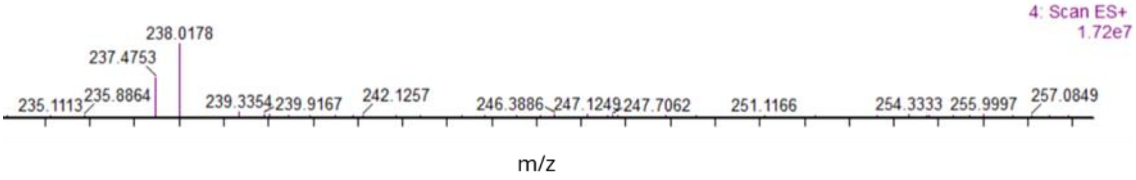

Figure S4.5: MS spectra of **e1**

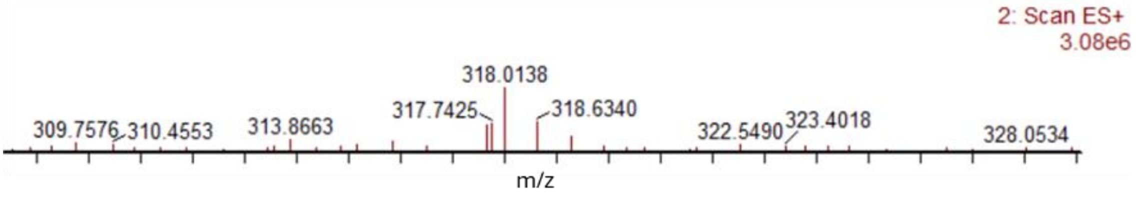

Figure S4.6: MS spectra of **f1**
